# Metal-coordinated polybenzimidazole membranes with preferential K^+^ transport

**DOI:** 10.1038/s41467-023-36711-w

**Published:** 2023-03-01

**Authors:** Jine Wu, Chenyi Liao, Tianyu Li, Jing Zhou, Linjuan Zhang, Jian-Qiang Wang, Guohui Li, Xianfeng Li

**Affiliations:** 1 Division of Energy Storage, Dalian Institute of Chemical Physics, Chinese Academy of Sciences, Dalian, 116023 China; 2 University of Chinese Academy of Sciences, 100049 Beijing, China; 3 Laboratory of Molecular Modeling and Design, State Key Laboratory of Molecular Reaction Dynamics, Dalian Institute of Chemical Physics, Chinese Academy of Sciences, Dalian, 116023 China; 4 Key Laboratory of Interfacial Physics and Technology, Shanghai Institute of Applied Physics, Chinese Academy of Sciences, Shanghai, 201800 China

**Keywords:** Batteries, Polymers, Coordination polymers

## Abstract

Membranes with fast and selective ion transport are essential for separations and electrochemical energy conversion and storage devices. Metal-coordinated polymers are promising for fabricating ion-conducting membranes with molecular channels, however, the structures and ion transport channels remain poorly understood. Here, we reported mechanistic insights into the structures of metal-ion coordinated polybenzimidazole membranes and the preferential K^+^ transport. Molecular dynamics simulations suggested that coordination between metal ions and polybenzimidazole expanded the free volume, forming subnanometre molecular channels. The combined physical confinement in nanosized channels and electrostatic interactions of membranes resulted in a high K^+^ transference number up to 0.9 even in concentrated salt and alkaline solutions. The zinc-coordinated polybenzimidazole membrane enabled fast transport of charge carriers as well as suppressed water migration in an alkaline zinc-iron flow battery, enabling the battery to operate stably for over 340 hours. This study provided an alternative strategy to regulate the ion transport properties of polymer membranes by tuning polymer chain architectures via metal ion coordination.

## Abstract (web summary)

Membranes with fast and selective ion transport are essential for electrochemical processes. Here the authors provide mechanistic insights into the structures of metal-ion coordinated polybenzimidazole membranes and the preferential K^+^ transport, and their application in an alkaline zinc-iron flow battery.

## Introduction

Ion-selective membranes are essential for a wide range of industrial separation and electrochemical processes^1^, such as ion extraction or recovery from wastewater and brine^2^, removal of contaminants such as salts and heavy metals^3^, and flow batteries^4^. These processes usually involve separations of specific ionic species from complex mixtures, which require membranes with enhanced ion selectivity^1^. Commercial ion-exchange membranes, such as perfluorinated membranes (e.g., Nafion®), are expensive and usually exhibit low selectivity due to large water channels^5,6^. A wide range of synthetic polymer ion-exchange membranes have been developed, however most ion-exchange polymers usually have nanophase-separated morphology, leading to compromised selectivity^7,8^.

Designing ion-selective membranes with ordered ion transport channels has attracted significant interests in recent years^9^. Typical strategies include synthetic polymers with precise chain lengths and chain microstructures^10^, metal-organic framework^11^ and covalent-organic frameworks with ordered ion channels^12^, or high-free-volume polymers with intrinsic micropores^13–16^. These synthetic membranes with subnanometer-sized channels facilitate vehicular transport and enhance ionic conductivity, however, the membrane selectivity can be compromised in concentrated electrolyte solutions due to weakened Donnan exclusion^17^. Introducing functional groups that have specific interactions with ions could effectively improve ion selectivity. An alternative strategy to regulation of ion transport channels is development of metal-ion coordination polymers. For example, Cu^2+^ coordination of densely packed cellulose expanded the spacing between polymer chains, forming nanosized ion channels with fast and selective lithium ion transport^18^.

Polybenzimidazole (PBI) are promising materials for use in a variety of electrochemical devices, such as high-temperature proton exchange membrane fuel cells^19^, and flow batteries^20^. Compared to conventional polymers, PBI polymers are chemically stable in acidic and alkaline electrolytes^21^. However, one key scientific challenge of PBI polymers is their low intrinsic ion conductivity, due to dense packing of PBI chains induced by strong hydrogen bonding. One promising approach is introducing coordination bonds between imidazole groups with metal ions (e.g. Co^2+^, Cu^2+^, Zn^2+^) to form coordinated networks^22^. The resulting coordinated polymer membranes exhibited higher fractional free volume and molecular sieving performance^23^. Our recent work also suggested that the Cu^2+^-coordination of PBI introduced crosslinking between polymer chains and facilitated cation transport^24^. However, the structures of metal coordinated PBI and ion transport mechanism remained poorly understood.

**Fig. 1:**
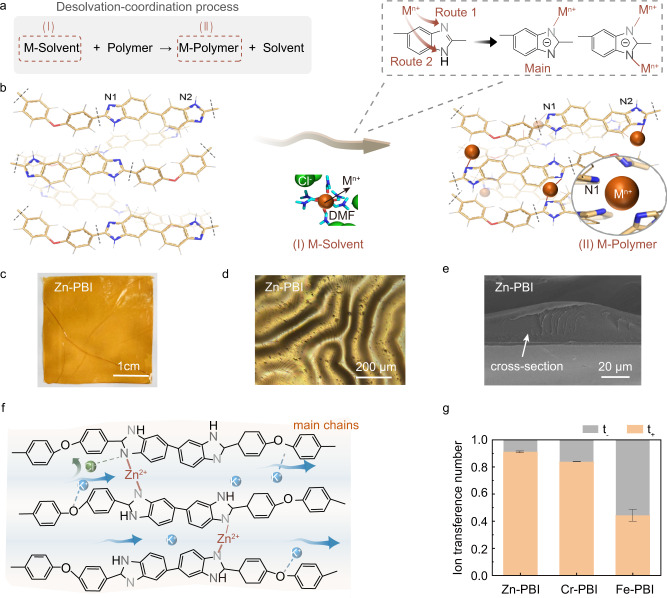
Metal-ion-coordinated polymer membranes with selective transport channels. **a** The desolvation-coordination process involving desolvation of metal ions and coordination with PBI polymer to form metal-polymer coordination networks. **b** Schematic illustration of formation of metal-coordination network. All solvated metal ions (M-Solvent) were expected to undergo (I) desolvation (N,N-dimethylformamide, DMF) first and then (II) coordinate with the imidazole groups on PBI chains to form M-PBI, involving two routes for metal ions (M^n+^) interacting with PBI: Route 1, metal coordination with pyridine nitrogen on imidazole group (N1); Route 2, metal coordination with pyrrole-like nitrogen on imidazole (N2). The morphology of Zn-PBI membrane. **c** photo of one piece of membrane; **d** optical image of surface; **e** cross-sectional SEM image. **f** Schematic diagram showing the rapid selective transport of K^+^ in Zn-PBI coordination network. **g** The K^+^ (t_+_) and Cl^−^(t_-_) ion transference number for Zn-PBI, Cr-PBI, and Fe-PBI membranes. Measurements were tested in the KCl solution with a gradient of 0.033 and 0.33 mol L^−1^. Error bars, mean ± standard deviation (s.d.). Source data are provided as a Source Data file.

Herein, we reported in-depth fundamental understanding of the structures of metal ion coordinated PBI membranes and the selective K^+^ transport via combined experimental characterization and molecular simulations. Metal ions coordinated with the imidazole groups on PBI polymer chains, forming rigid metal-coordination networks (Fig. 1a, b). The mechanical stress induced by the coordination reactions formed crumpled surface structures (Fig. 1c–e) The degree of metal ion coordination can be controlled by the reaction rates between different metal ions (Zn^2+^, Cr^3+^, Fe^3+^) and polymers. By reducing the coordination rate, e.g. Zn^2+^ coordination with PBI, uniform coordination networks could be obtained. The resulting metal-coordinated networks formed continuous water channels, and exposed more polar groups inside the channels (especially oxygen atoms) to assist cation (K^+^) transport (Fig. 1f). The K^+^ transference number in Zn-PBI was as high as 0.91, which was higher than that of polymers free of ion exchange groups^18^, and comparable to cation exchange membranes (nearly 1)^25,26^ (Fig. 1g). Furthermore, the ion transport in Zn-PBI channels was not substantially affected by the solution concentration^27^. The Zn^2+^-coordination domains in Zn-PBI further increased the local surface charge density near the Zn^2+^ sites and promoted Donnan exclusion effect. The preferential K^+^ transport in Zn-PBI was retained even in a concentrated KCl solution up to 3 mol L^−1^ as well as in alkaline KOH solution. The high K^+^ selectivity of Zn-PBI was beneficial for reducing water transmembrane migration in an alkaline zinc-iron flow battery (AZIFB), leading to stable operation of zinc-based flow batteries.

## Results

### Synthesis and characterization of ion-coordinated membranes

Here, metal-ion coordinated PBI membranes were prepared via coordination-induced phase transformation (Methods)^24^. The polymer solution was cast on a substrate and then transferred into an organic solvent solution containing metal ions (Zn^2+^, Cr^3+^, and Fe^3+^). The resulting coordinated membranes were termed as M-PBI (Zn-PBI, Fe-PBI, and Cr-PBI).

To understand the evolution of metal coordination reactions and sites of coordination, we performed more in-depth characterization analyses. The overall reaction was depicted in Fig. 1a. All solvated metal ions were expected to undergo desolvation (*N,N*-dimethylformamide, DMF) first (I) and then coordinate with the imidazole groups on PBI chains (II). The strong DMF solvation of metal ions in Step I was verified by ^1^H nuclear magnetic resonance (NMR) spectroscopy (Supplementary Fig. 1a), Fourier transform infrared (FTIR) spectroscopy (Supplementary Fig. 1b), and Raman spectroscopy (Supplementary Fig. 1c, d). For Step II, the coordination of PBI with the metal ions were confirmed by FTIR spectroscopy (Supplementary Fig. 1e), X-ray photoelectron spectroscopy (XPS) (Supplementary Fig. 2). XPS peak area evolution analysis (Supplementary Fig. 2a) and density functional theory (DFT) calculations (Supplementary Fig. 3a–d) further suggested that pyridine-like nitrogen –N=(N1) was the preferred reaction site for PBI chains. Notably, highly reactive metal ions such as Cr^3+^ and Fe^3+^ also interacted with pyrrole-like nitrogen -NH- (N2) to some extent, while the coordination interactions were not as strong as that of pyridine-like nitrogen, as confirmed by ^1^H NMR (Supplementary Fig. 3e). Therefore, we could conclude that there are two routes in the overall coordination reactions (Fig. 1b). Zn^2+^, Cr^3+^, and Fe^3+^ mainly linked with –N=(N1) of the PBI chains, while more reactive Cr^3+^ and Fe^3+^ ions also coordinated with -NH- (N2). Assuming =N- (N1) was the binding site, the reaction rate followed the order, Cr^3+^ > Fe^3+^ > Zn^2+^, based on the theoretical energy estimated by DFT calculations (Methods). It should be noted that the coordination reactions were generally fast, so it was difficult to quantify the rates. We performed in situ optical microscopy imaging after dropping the ZnCl_2_/DMF solution on the surface of the PBI gel. Crumpled morphology emerged on the membrane surface within 1 second and fully formed in 3 s (Supplementary Movie 1, Supplementary Fig. 4).

The coordination reactions influenced the membrane morphology, as observed by scanning electron microscopy (SEM). When PBI polymer solution was immersed in an organic solvent without metal ions (PBI-D) or directly formed a membrane in water (PBI-water), opaque membranes were formed with macroporous structures (~0.8 μm) (Supplementary Fig. 5)^4^. In contrast, when metal ions participated in membrane formation, the resulting coordinated membranes were visually transparent and cross-sectional SEM images confirmed dense structures, indicating that the coordination reaction occurred throughout the whole PBI layer (Supplementary Fig. 6). As characterized by ultradeep surface morphology determination microscopy, undulating stripes appeared on the surface of Zn-PBI, while Cr-PBI and Fe-PBI appeared flat (Fig. 1c, Supplementary Fig. 6a). Zn^2+^ coordination induced macromigration of the polymer segments, forming macroscopic surface undulation stripes. Furthermore, coordination between metal ions and PBI had similar effect of crosslinking, which efficiently increased the rigidity and stability of the membranes^28^, as shown by the better organic solvent resistance (Supplementary Fig. 7), thermal gravimetric analysis (TGA) (Supplementary Fig. 8), tensile stress tests (Supplementary Table 1) and nanoindentation results (Supplementary Fig. 9, Supplementary Table 2).

Metal ions coordination with polymer chains strongly influenced the size and distribution of free volume elements in the membranes. Zn^2+^ coordinated polymer chains tightly, as reflected by the gas flux through the membranes (Supplementary Table 1). The free volume of M-PBI was probed by positron annihilation lifetime spectroscopy (PALS) (Supplementary Fig. 10). The *o*-Ps lifetime τ_3_ distribution and *o*-Ps intensity I_3_ were used to estimate the average free volume cavity radius. Zn-PBI exhibited the smallest cavity radius (0.332 nm), and the cavity number density in the bulk was significantly higher than those of Cr-PBI and Fe-PBI. The PALS result implied that for Zn-PBI, the resulting coordination network formed more uniform subnanometre channels. Inductively coupled plasma‒mass spectrometry (ICP‒MS) suggested that approximately 5.33 wt% Zn, 0.34 wt% Cr, and 7.14 wt% Fe participated in the formation of Zn-PBI, Cr-PBI, and Fe-PBI, respectively (Supplementary Table 3). Extended X-ray absorption fine structure (EXAFS) and X-ray absorption near edge structure (XANES) further confirmed that these metal ions formed multiple linkages with pyridine nitrogen in the imidazole groups on PBI chains (Supplementary Fig. 11), and the coordination numbers and average bonding distance were listed in Supplementary Table 4. Based on the ICP and elemental analysis, we estimated that on average two PBI segment repeating units coordinated with two Fe^3+^ ions or one Zn^2+^ ion but only 0.08 Cr^3+^ ion, respectively (Supplementary Table 3).

**Fig. 2:**
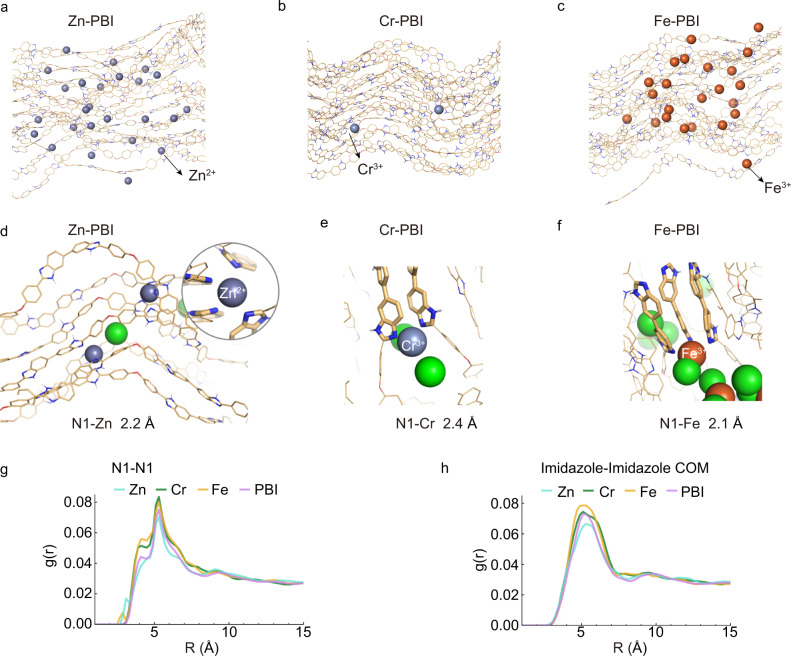
Molecular simulation of metal-coordinated PBI membrane. Illustration of different coordination domains in **a** Zn-PBI, **b** Cr-PBI, **c** and Fe-PBI (right). **e**, **f** Strong metal ion-PBI interactions at N1 with close distances were sampled in molecular dynamics simulations. **d** Zn-PBI, **e** Cr-PBI (middle), and **f** Fe-PBI. The radial distribution function *g*(r) for **g** N1-N1 and **h** the center-of-mass (COM) of imidazole-imidazole in PBI chains based on the results of simulation statistics.

To further understand the coordination interactions between metal ions and polymer chains, we performed molecular dynamics (MD) simulations to study the membrane structures, as depicted in Supplementary Figs. 12–21 and Methods. Figure 2a–c shows different coordination domains in Zn-PBI, Cr-PBI, and Fe-PBI. Figure 2d–f and Supplementary Fig. 13 show the strong metal ion-PBI interactions at close distances (2.0~2.4 Å) sampled in the MD simulations, in agreement with the synchrotron radiation data. The simulations showed that metal ions altered the packing state of chain segments to become contorted to varying degrees.

In terms of the radial distribution function *g*(r) for N1-N1 (Fig. 2g) and interimidazole (Fig. 2h, Supplementary Fig. 16c) and interactions in the PBI chains, Fe^3+^ and Cr^3+^ induced different degrees of local constriction between imidazole units, forming relatively disordered channels (~4 Å for N1-N1 and ~5 Å for inter-imidazoles). In contrast, Zn^2+^ coordination resulted in more uniform distribution of coordination domains, which generated narrowly distributed channels. In this circumstance, Zn-PBI exhibited a relatively higher packing density in the whole three-dimensional space than Cr-PBI and Fe-PBI.

The reaction rates of metal ion coordination played a crucial role on the structural evolution and morphology. From the kinetics perspective, the rates of metal ions coordination led to varied degree of rigidity of polymer chains. Generally, a relatively lower reaction rate was more favorable to generating uniform coordination networks. If the coordination occurred rapidly, such as the case with Cr^3+^, it would be detrimental to the structural integrity of the membrane (Supplementary Fig. 6c). For high-reactivity Fe^3+^, rapid coordination induced excessive segment migration at the coordination domains, leading to uneven crosslinking and formation of larger channels in Fe-PBI. By contrast, the relatively low reaction rate between Zn^2+^ and PBI resulted in more uniformly distributed coordination domains. All these results indicated that the degree of coordination can be regulated by controlling the reaction rate between metal ions and polymers. The simple metal-coordination strategy was expected to adjust and diversify the internal pore architectures of membranes.

### Selective ion transport

**Fig. 3:**
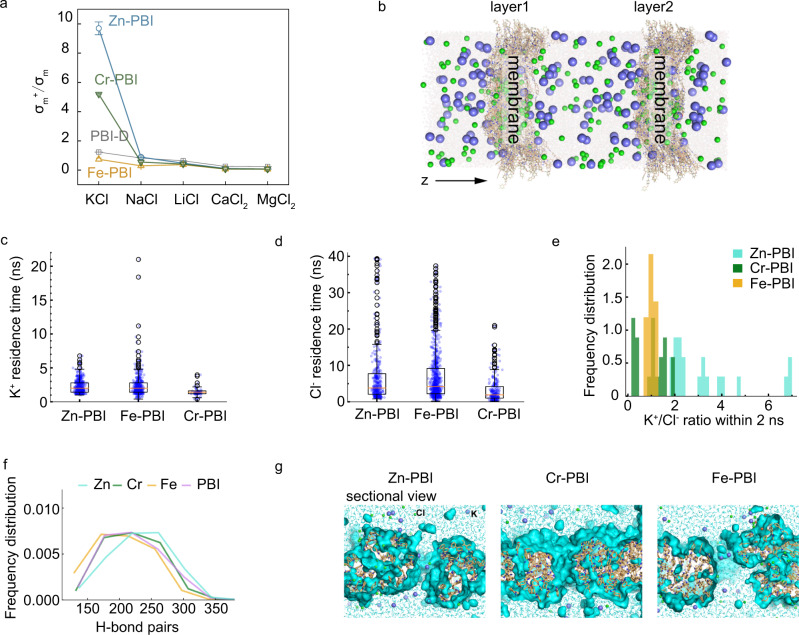
Molecular dynamics simulation of ion transport. **a** Distinct cation selectivity in different membranes, where σ_m_ is the ion conductivity of salt and σ_m_^+^ is the ion conductivity of cation ions. Error bars, mean ± standard deviation (s.d.). Source data are provided as a Source Data file. **b** A double-layer system with two aqueous compartments filled with K^+^ (in blue) and Cl^−^ (in green). Water molecules are not shown. **c**–**e** Box plot of K^+^ or Cl^−^ residence time in the PBI membrane. **c** The total numbers of K^+^ transported through the membranes are 458, 431, and 63 for Zn-, Cr-, and Fe-PBI, respectively; **d** the total numbers of Cl^−^ transported are 347, 761, and 234 for Zn-, Cr-, and Fe-PBI, respectively. **e** Frequency distribution of the K^+^/Cl^−^ ratio within 2 ns as the fast transport period in double-layer Zn-, Cr-, and Fe- PBI systems. **f** Hydrogen bonds formed between PBI (at N1, N2, and O) and water molecules in Zn-, Cr-, Fe- and metal-free PBI systems. Zn-PBI system tends to expose more polar groups to solvent. **g** Sectional views of water accessible surface in double-layer Zn^2+^, Cr^3+^, and Fe^3+^ restrained PBI systems. Cl^−^ were shown in green sphere, K^+^ were shown in blue sphere, and water molecules are shown in cyan surface and thin stick. By comparing the pore width in Zn^2+^, Cr^3+^, and Fe^3+^ restrained PBI membrane, Zn-PBI formed 10~12 Å wide pore; Fe-PBI formed elongate pore with changing width of 11~16 Å; Cr-PBI formed small pore of ~6 Å.

The metal coordinated PBI networks formed nanosized ion channels and the distribution of atoms and surface charge density on the pore walls, which enable fast and selective ion transport. We measured the diffusion of typical salt ions, including K^+^, Na^+^, Li^+^, Ca^2+^, and Mg^2+^, with hydrated diameters in the range of 6.6~8.6 Å^29,30^. Figure 3a showed the cation selectivity of PBI and coordinated PBI membranes. In the KCl solution, Zn-PBI showed ten times higher selectivity for K^+^ over counterion Cl^−^. In contrast, Cr-PBI showed moderate K^+^ selectivity while Fe-PBI exhibited negligible K^+^ selectivity, similar to the control sample PBI-D without metal ion coordination. To quantify the ion transference number, we performed ion transference number tests by measuring the voltage value at the zero apparent current of a concentration cell, following a protocol reported previously^31^. Zn-PBI featured a high K^+^ transference number (t_+_) of 0.91 and Cl^−^ transference number of 0.09 (Fig. 1g). The K^+^ transference number was much higher than that of polymers without ion exchange groups reported recently (0.78 for Cu^2+^-coordinated cellulose)^18^, which was comparable to that of cation exchange membranes (nearly 1)^25,26^. In contrast, the Fe-PBI membrane with disordered coordination network exhibited a t_+_ value of 0.5, which was almost the same as the t_+_ in the bulk solution, confirming the poor selectivity in Fe-PBI.

To further study the K^+^ transport mechanisms in Zn-PBI, molecular dynamics simulation was performed with a double-layer ion transport model constructed. The model included two aqueous compartments in which K^+^ and Cl^−^ were initially kept between two identical membranes, allowing the K^+^ ions to diffuse to either side of the membranes (Fig. 3b and Supplementary Fig. 18). We repeated the diffusion process 20 times and determined the numbers and residence time of K^+^ and Cl^−^ transported through the membrane. The K^+^ residence time for Zn-PBI (1~6.8 ns) was approximately 2 ns on average, while the range widened (0.4~21 ns) for the Fe-PBI membrane. Fewer K^+^ ions transported through Cr-PBI with a residence time in the range of 0.4~4 ns. The Cl^−^ residence time in Zn-PBI (0.8~40 ns) was higher than those observed in the Fe-PBI and Cr-PBI membranes. Here, we selected ion transport within 2 ns as the fast transport period, as indicated in Fig. 3c–d, and quantified the frequency distributions of the K^+^/Cl^−^ transport ratio (Fig. 3e). Zn-PBI exhibited a K^+^/Cl^−^ transport ratio in the range of 2~7, while the K^+^/Cl^−^ transport ratio was close to 1 for Fe-PBI and slightly greater than 1 for Cr-PBI, which were consistent with the experimental trend.

The ion transport within the metal-ion coordinated polymer network was influenced by the complex environment involving physical confinement, ionic and electrostatic interactions. First, the inner structure of the PBI membrane was changed by Zn^2+^. Inside the structures of Zn-PBI, the uniform Zn^2+^ coordination resulted in more dispersion among the imidazole units of the polymer chains, expanding the inner water accessible surface as indicated by the increased hydrogen bonds formed between PBI (by polar groups N1, N2, and O) and water molecules in the Zn-PBI system (Fig. 3f). The cross-sectional view (Fig. 3g) and top views (Supplementary Fig. 19) of water accessible surface showed that inside Zn-PBI, uniform Zn^2+^ coordination resulted in more dispersion of water clusters within the polymer networks, which may form interconnected water channels for ion diffusion. Fe^3+^ caused different degrees of local conformation and aggregation between imidazole units, resulting in uneven water distributions in different regions of the polymer matrix.

**Fig. 4:**
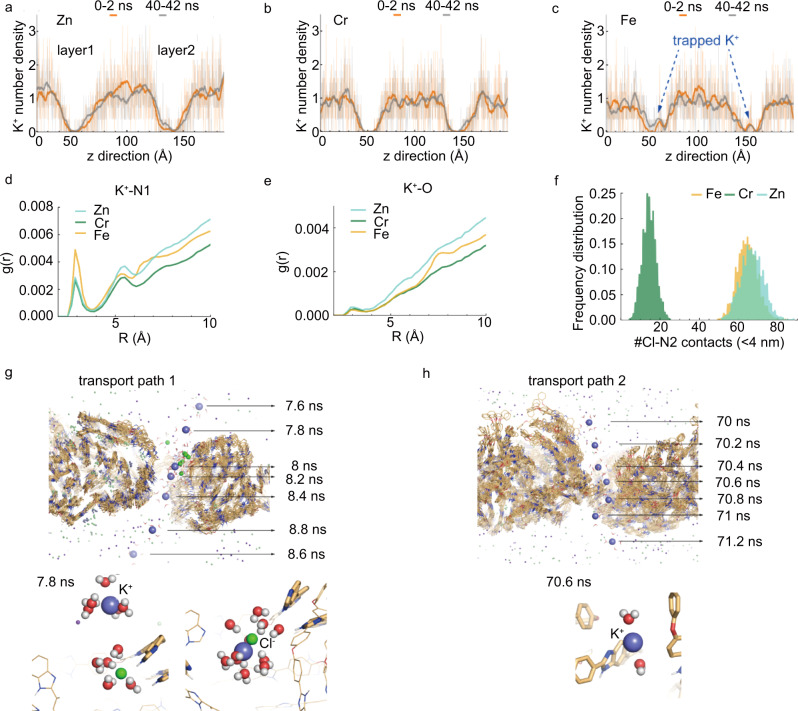
Molecular dynamics simulation of K^+^ transport in metal-coordinated PBI membranes. **a**–**c** K^+^ number density in 0.25 Å-slices along the z-axis in the beginning and after 40 ns in double-layer **a** Zn^2+^ **b** Cr^3+^ and **c** Fe^3+^ coordinated PBI systems. Radial distribution function *g*(r) of K^+^ to each **d** pyridine-like nitrogen –N=(N1) and **e** oxygen of PBI in Zn^2+^, Cr^3+^ and Fe^3+^ coordinated PBI system. **f** Frequency distribution of Cl^−^ within 4 Å of pyrrole-like nitrogen -NH- (N2) of PBI. **g**, **h** Two transport paths for K^+^ from double-layer Zn^2+^ coordinated PBI system. **g** Snapshots of transport path 1 showed one K^+^ (in blue) to pass through Zn-PBI membrane under regulation of Cl^−^ (in green). Bottom: Zoom-in sequent snapshots are displayed for K^+^ with water in the first solvation shell. **h** Snapshots of transport path 2 showed snapshots of K^+^ along to pass through Zn-PBI membrane. bottom: Zoom-in snapshot is displayed for K^+^ with water in the first solvation shell.

Molecular simulations also provided insights into the ionic interactions of cations and anions with the coordinated polymer networks. We calculated the K^+^ number density along the z-direction to membranes in the double-layer ion transport model. As shown in Fig. 4a–c, the membrane region K^+^ peaks in Fe-PBI implied that K^+^ was trapped in Fe-PBI, while K^+^ ions could freely diffuse through Zn-PBI. The apparent K^+^ binding with –N=(N1) in the channels of Fe-PBI (Fig. 4a–d) led to a longer K^+^ residence time (Fig. 3c). The simulation suggested that K^+^ ions interacted with the abundant oxygen–containing flexible ether linkages and diffused between them, similar to the acceleration of K^+^ assisted by oxygen atoms in H_2_O^32^ (Fig. 4e). The molecular dynamics simulation revealed that although K^+^ binding with oxygen in the ether linkages was much weaker than that from pyridine-like nitrogen –N=(N1) at ~4 Å, Zn-PBI attracted more K^+^ around oxygen than either Fe- or Cr- PBI along the radial distribution (Fig. 4e). Additionally, the probability of K^+^ interacting with oxygen (within 4 Å) in Zn-PBI was greater than that in other membranes (Supplementary Fig. 21b), which greatly helped to facilitate K^+^ transport^32^.

The interactions and transport of counter-anions (Cl-) within the Zn-PBI coordinated polymer network also played important roles in regulating the preferential K^+^ transport (Fig. 4f and Supplementary Fig. 21c, d). Molecular dynamics simulation suggested two major transport pathways of K^+^ in Zn-PBI recorded in 0.2 ns intervals. As shown in Fig. 4g, in transport path 1, Cl^−^ was immobilized by -NH- (N2) and modulated K^+^ to pass through rapidly. Specifically, Fig. 4f showed Zn-PBI attracted more Cl^−^ around -NH- (N2) at ~3.5 Å. The effective-stacked chains of Zn-PBI enabled the large unreacted -NH- (N2) to easily capture Cl^−^ to form temporary adsorption, impeding mobility of Cl^−^ and in turn improving the selectivity of K^+^. In transport path 2 (Fig. 4h), K^+^ was directly transported through a pore, during which K^+^ was found to mainly interact with –N=(N1) and near the oxygen-containing ether linkages on polymer chains.

### K^+^-selective Zn-PBI membrane for flow batteries

To demonstrate the utility of the metal-ion coordinated PBI membranes, we integrated as an ion-selective membrane for the alkaline zinc-iron flow battery (AZIFB). Membranes should allow the fast and selective transport of charge carrier K^+^ while restrict the crossover of redox active species, enabling high energy efficiency and stable operation of AZIFB. In addition, water migration associated with crossover of redox species is another critical issue for operation of flow batteries. The water transmembrane migration triggered an electrolyte imbalance after long-term operation of a battery, which may eventually cause the battery to fail^33^. For an AZIFB, water generally moved towards the positive half-cell, increasing the volume on that side (for a detailed discussion, see Supplementary Fig. 22). This may be because negatively charged active species carried water and migrate towards the positive half-cell driven by the electric field^34^. Additionally, [Zn(OH)_4_]^2^ was reduced to Zn when charging, resulting in an imbalance of ionic strength (also concentration gradient) and thus causing water to migrate^35,36^.

**Fig. 5:**
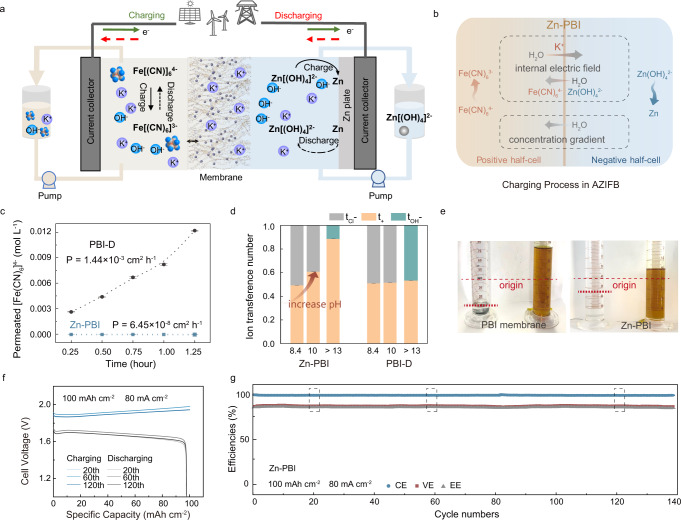
Application of K^+^ selective membrane in alkaline zinc-iron flow battery (AZIFB). **a** The illustration of AZIFB. **b** Water migration during the charging process in an AZIFB. **c** The [Fe(CN)_6_]^4−^ permeability test at 1.25 h. Error bars, mean ± standard deviation (s.d.). **d** The ion transference number was tested in a high concentration KCl solution (1 and 3 mol L^−1^) and KOH solution (0.3 and 3 mol L^−1^). Adjusting PH from 8.4 to 10 of 1 and 3 mol L^−1^ KCl solution with the dilute KOH solution. The average concentration of 0.3 and 3 mol L^−1^ KOH solutions has a PH value greater than 13. Error bars, mean ± standard deviation (s.d.). Source data are provided as a Source Data file. **e** Photos showing the change of electrolyte volume after battery cycling when using the PBI membrane (failed after 30 h of operation) and Zn-PBI (stopped manually after 50 h of operation). Even compared with a denser PBI membrane, Zn-PBI can mitigate water migration. The detailed experiments are described in the Methods and Supplementary Fig. 22. **f** Cell voltage profiles at the 20^th^, 60^th^, and 120^th^ cycles for AIZFB using Zn-PBI. **g** The cycling performance of AIZFB using Zn-PBI at a current density of 80 mA cm^−2^. Current efficiency (CE), voltage efficiency (VE), energy efficiency (EE).

In this work, we show that all issues can be solved by using the selective Zn-PBI membranes which could balance ionic strength during charging and suppress water transmembrane migration during the battery operation (Fig. 5b). Firstly, the selective Zn-PBI membrane mitigated water migration by reducing the crossover of redox species. Zn-PBI possessed a denser interior structure and more efficiently blocked the active species [Fe(CN)_6_]^4−^ and [Zn(OH)_4_]^2−^ than PBI-D. In the permeability test, Zn-PBI demonstrated a low permeation coefficient for [Fe(CN)_6_]^4−^ (6.45 × 10^−8^ cm^2^ h^−1^, compared 1.44 × 10^−3^ cm^2^ h^−1^ for PBI-D) and [Zn(OH)_4_]^2−^ (4.27 × 10^−7^ cm^2^ h^−1^, compared 1.98 × 10^−4^ cm^2^ h^−1^ for PBI-D) (Fig. 5c, Supplementary Fig. 23b, c).

Secondly, Zn-PBI facilitated K^+^ transport during battery operation, which moved in the direction opposite to transmembrane water. In the results presented above, we found that Zn-PBI exhibited high K^+^ transport in KCl aqueous solution. But for KOH, OH^−^ was reported to be more competitive with K^+^ for transmembrane transport^14^. Moreover, the concentration of alkaline solution may also vary the polymer chain packing and ion transport properties. It had been reported that PBI membranes swelled in a high-concentration KOH solution^37^, leading to formation of large ion channels and the transport of OH^−^ may become dominant. More importantly, in a 3 mol L^−1^ high-concentration solution, an electrical double layer (EDL) was inevitably compressed, thus weakening Donnan exclusion and reducing ion selectivity^27^.

Hence, we first verified the Zn-PBI maintains the preferential K^+^ transport in a 3 mol L^−1^ KOH solution at the same concentration as the battery electrolyte. Owing to the confinement in the Zn^2+^-coordinated network, the thickness of EDL and the electrostatic interaction range (Debye length, λ_D_) covered more ions passing through membranes. When the concentration of the KCl solution increased from 0.33 M to 3 M, the λ_D_ was reduced from ~0.529 nm to ~0.176 nm^38^, which was theoretically narrower than the average free volume cavity radius (R) of Zn-PBI (0.332 nm) as measured from PALS. The transference number of K^+^ in Zn-PBI was enhanced by adjusting the pH from 8.4 to 10 (Fig. 5d), while PBI-D did not show such pH dependence. To demonstrate this phenomenon, we quantified the surface charge density of Zn-PBI according to the Gouy-Chapman equation by evaluating the zeta potential (Supplementary Fig. 25a, b)^39^. When the pH increased from 3 to 10, Zn-PBI became more negatively charged due to deprotonation of the imidazole groups at high pH. Therefore, with the pH increased from a neutral to alkaline electrolyte, the electronegativity of the main chains increased, especially for the segments coordinated with Zn^2+^, which expedited K^+^ movement (Supplementary Fig. 24b, Supplementary Fig. 25b). We further tested the K^+^ transference number of Zn-PBI in a 3 mol L^−1^ KOH concentrated solution. The K^+^ transference number could be further improved to 0.886 at a pH over 13 in KOH (Fig. 5d). These results indicated the dependable high K^+^-selective performance of Zn-PBI in a 3 mol L^−1^ KOH solution.

Moreover, Zn-PBI exhibited enhanced hydrophilicity further benefited ion transport at the interface (Fig. 5c). The superior t_+_ (0.886) implied that the current circuit was mainly carried by K^+^ with minimal membrane resistance (0.59 Ω cm^2^) within Zn-PBI compared with PBI-D (t_+_ = 0.531, 1.30 Ω cm^2^) (Supplementary Fig. 25c, Supplementary Table 1). As the water carried by K^+^ can partially counteract the water transfer, therefore, Zn-PBI can reduce the water migration effectively (Fig. 5e).

In addition to the ability to mitigate crossover contamination and imbalance electrolyte, Zn-PBI can further effectively suppress zinc dendrites in AZIFB with high areal capacity by enabling more zinc deposition space provided by the crests and troughs of patterns^24^. Zn-PBI membrane showed high stability towards dendrite growth and could also withstand the strongly alkaline environment and enabled long-term battery operation. In the presence of Zn-PBI, the cycling performance of zinc symmetric flow battery was superior even at a high current density of 80 mA cm^−2^ and an areal capacity of 40 mAh cm^−2^, resulting in the beaded deposition tendency of zinc (Supplementary Fig. 26a). In contrast, an identical zinc symmetric flow battery assembled with PBI-D membrane failed after 65 h, and subsequently, PBI-D membrane was punctured by zinc dendrites, and the battery was short-circuited and eventually failed.

Owing to the combined characteristics of fast and selective K^+^ transport, Zn-PBI enabled an AZIFB to work at current densities varying from 40 to 160 mA cm^−2^ at an areal capacity of 100 mAh cm^−2^ (Supplementary Fig. 26b). As shown in Fig. 5f and Fig. 5g, an AZIFB using Zn-PBI can stably operate for more than 340 hours and approximately 140 cycles, even at a high current density of 80 mA cm^−2^, exhibiting an energy efficiency (EE) of 86.7%. The battery showed a cycling stability with a stable discharge capacity of about 100 mAh cm^−2^ for nearly 140 cycles. This performance even surpassed that of a commercial cation exchange membrane (Nafion 212), which was unable to withstand the zinc dendrite and can only enable the AZIFB to run for less than 50 hours under the same operation conditions (Supplementary Fig. 27).

## Discussion

Overall, we reported mechanistic study of the structures of metal-ion coordinated polybenzimidazole membranes and the mechanism of selective K^+^ transport in membranes. With a relatively lower reaction rate, uniform Zn^2+^ coordination enhanced the rigidity of polymer network and formed continuous water channels and exposed more polar groups. Molecular dynamics simulation and characterization analysis suggested that the fast and selective transport of potassium ions in the metal ion coordinated PBI network could be attributed to combined effects of (1) open nanosized ion channels, (2) coordination of K^+^ with functional groups and facilitated transport, (3) binding and immobilization of counter-anions (Cl^−^, OH^−^), and (4) electrostatic charge induced by metal ion coordination. The resulting Zn-PBI membranes can not only effectively hinder active species but also expedite the transport of K^+^ charge carriers. Such preferential K^+^ transport could effectively balance ionic strength during charging and suppress water transmembrane migration. The K^+^ selective membrane enabled the stable operation of an alkaline zinc-iron flow battery for over 340 h at a high current density of 80 mA cm^−2^. We expected that the fundamental understanding of the structures of metal-coordination polymer membranes as well as the ion transport mechanism would inspire the development of novel ion-selective membranes for application in separation and electrochemical devices.

## Methods

### Materials

Potassium hydroxide (KOH), sodium hydroxide (NaOH), potassium chloride (KCl), sodium chloride (NaCl), lithium chloride (LiCl), calcium chloride (CaCl_2_), and magnesium chloride (MgCl_2_) were purchased from Aladdin (China). Potassium ferrocyanide (K_4_[Fe(CN)_6_·3H_2_O]), potassium sulfate (K_2_SO_4_), Sodium Hexacyanoferrate(II) (Na_4_[Fe(CN)_6_]·12H_2_O), zinc oxide (ZnO) were purchased from Kermel. *N*, *N*-Dimethylformamide (DMF), FeCl_3_ was purchased from Sinopharm Chemical Reagent Co., Ltd (China). CrCl_3_·6H_2_O was purchased from Macklin. ZnCl_2_ was purchased from Ourchem. Polybenzimidazole (PBI) and PBI membrane were used as received.

### Fabrication of M-PBI membranes

First, the 20 wt% PBI casting solution and 2 mol L^−1^ M^n+^-reaction solution were provided, where M^n+^ standards for different transition metal ions including Cr^3+^, Fe^3+^, and Zn^2+^. To ensure the casting solution was homogenous, certain PBI was added in DMF and stirred overnight. Then dissolved casting solution stood for at least 12 h to remove the bubble. The M^n+^-reaction solution was prepared by adding and dissolving certain CrCl_3_·6H_2_O, FeCl_3_, and ZnCl_2_ in DMF.

Subsequently, the casting solution was cast on a clean and dust-free glass substrate with a blade of 200 μm, which was then transferred into the prepared M^n+^-reaction solution. After immersing for 4 min to ensure the coordination reaction was complete, the above glass substrate was soaked in water. The membrane was peeled off the glass substrate and then washed and stored in water, which was named M-PBI (Cr-PBI, Fe-PBI, Zn-PBI).

### Fabrication of PBI-D and PBI-water membranes

The same 20 wt% PBI casting solution was prepared as above described. Subsequently, the polymer solution was cast on a clean and dust-free glass substrate with a blade of 200 μm, and it was then transferred into the pure DMF. After immersing for 4 min, the above glass substrate was soaked in water to obtain PBI-D.

PBI-water was prepared directly formed via immersing coated PBI gel in water, which is the typical nonsolvent induced phase separation (NIPS).

### Membranes characterization

The cross-sectional and surface morphology of membranes were characterized by scanning electron microscopy (SEM, HITACHI SU1510) after fracturing them in liquid nitrogen and coating with gold (MSP-2S Magnetron Ion Sputter Metal Coating Device), operation at 15 kV. The surface morphology of membranes was obtained via an ultradeep surface morphology determination microscope (KEYENCE, VK-8510). The top-surface chemical analysis of membranes was determined by the Fourier transform infrared spectrometer (FTIR, Thermo Fisher Nicolet iS50) with the scan range from 1000 to 4000 cm^−1^, electron paramagnetic resonance (EPR, Bruker A200), X-ray photoelectron spectroscopy (XPS, Escalab 250Xi) with the binding energy range of 0–1400 eV using an Al Kα 1486.6 eV X-ray source, Raman spectrum (Bruker Optics, Senterra). The mechanical stability of the membranes was characterized by a universal testing machine (SANS, Saxes M2). The membranes were dissolved in the DMSO-d6, and the ^1^H nuclear magnetic resonance (NMR) spectroscopy was obtained via AVANCE III HD 700 MHz. The membrane thermal stability was characterized via the thermal gravimetric analyzer (TGA, PerkinElmer, Diamond). The elements analysis of membranes was obtained from the inductively coupled plasma mass spectrometry (ICP-MS) (PerkinElmer, NexION 350X) and ONH elemental analyzer (EMGA-930). The static contact angle on the top surface of the membranes was measured by the contact angle tester (JC-2000D, China). The microregions mechanical properties of membranes were characterized by nanoindentation (Bruker TI 950 TriboIndenter) loading Berkovich tip. The zeta potentials of membranes were measured by Surface Zeta Potential Analyser (Anton Paar, SurPASS3).

The H_2_ flux through membranes was carried out by gas permeation with gas compositions quantified by a gas chromatography (GC-2014, Shimadzu). All membranes were vacuum freeze-dried to maintain inner structure before the test.

Cr, Fe, and Zn K-edge X-ray absorption spectroscopy (XAS) were conducted at beamline 14W1 of the Shanghai Synchrotron Radiation Facility (SSRF). Data were recorded using a Si (111) double crystal monochromator. All XAFS data were analyzed using the program Demeter^40^. For all samples, the EXAFS oscillations were extracted from the normalized XAS spectra by subtracting the atomic background using a cubic spline fit to k^2^-weighted data, where k is the photoelectron wave number. The χ(k) functions were then Fourier transformed into R-space with the window in the k range 2.5–9.5 Å^−1^. The fitting procedure was performed on the k^2^-weighted FT-EXAFS. The amplitude reduction factor S_0_^2^ was obtained from the fitting of the foil reference and fixed in EXAFS fits.

### Area resistance of membranes

The area resistances of the membranes were obtained by electrochemical impedance spectroscopy (EIS, Solartron 1260 + 1287) as we reported before^31^, over a frequency range from 10 kHz to 2 MHz. Membranes were immersed in 3 mol L^−1^ KOH solution overnight in advance. The homemade conductivity cell was filled with 3 mol L^−1^ KOH and separated by a membrane with an effective area of 1 cm^2^. The area resistance was calculated as the resistance difference of a conductive cell with and without a membrane.

### Ion transport properties characterization of membranes

As we reported before^31^, the ion conductivity was carried out by Gamry Reference 3000 and Interface 1000 using the cyclic voltammetry measurement method. KCl solution (0.033 mol L^−1^, 0.33 mol L^−1^, 1 mol L^−1^, 3 mol L^−1^), NaCl solution (0.033 mol L^−1^, 0.33 mol L^−1^), LiCl solution (0.033 mol L^−1^, 0.33 mol L^−1^), CaCl_2_ solution (0.033 mol L^−1^, 0.33 mol L^−1^), MgCl_2_ solution (0.033 mol L^−1^, 0.33 mol L^−1^), and KOH solution (0.05 mol L^−1^ and 0.5 mol L^−1^) were made with ultra-pure water. The custom-made device was assembled as follows: The membrane was soaked into the lower-concentration aqueous solution for at least 6 hours before the membrane was tested.

The left reservoir contained the solution with a lower concentration, while the right reservoir contained the corresponding solution with a higher concentration. A carbon paper electrode and an Ag/AgCl reference electrode were inserted into each reservoir. The electrodes in the left and right reservoir were utilized as the working electrode and the reference electrode respectively. The corresponding CV curves were recorded at various room temperatures.

The calculation of ion transfer numbers in the membrane was described by the Nernst Eq. (1):

1 $${{{{{{\rm{V}}}}}}}_{0}=({{{{{{\rm{t}}}}}}}_{+}-{{{{{{\rm{t}}}}}}}_{-})(k{{{{{\rm{T}}}}}}/e){{{{{\rm{ln}}}}}}({{{{{{\rm{C}}}}}}}_{{{{{{\rm{high}}}}}}}/{{{{{{\rm{C}}}}}}}_{{{{{{\rm{low}}}}}}})$$

Where V_0_ is the voltage value recorded at the zero apparent current (i = 0), the t_+_ and t_-_ are respectively the ion transfer numbers for cations and anions, *k* is the Boltzmann constant, T is the test temperature, *e* is the elementary charge, C_high_/C_low_ here is 10.

The ion selectivity (σ_m_^+^/σ_m_) was calculated referred to the reference our group reported^20^. Where σ_m_ is the ion conductivity of salt and σ_m_^+^ is the ion conductivity of cation ions.

The PH was adjusted from 8.4 to 10 of KCl solution with the dilute KOH solution tested by PH meter (METTLER TOLEDO, S320-K).

### The properties of ion channels

The Debye length (λ_D_), which describes the thickness of EDL, varies inversely with the bulk ion concentration^27^. Debye length (λ_D_) was calculated as Eq. (2)^38^:

2 $${{{{{{\rm{\lambda }}}}}}}_{{{{{{\rm{D}}}}}}}={\kappa }^{-1}={\left(\frac{\varepsilon {RT}}{2{F}^{2}c}\right)}^{1/2}$$

The surface charge density (mC m^−2^) of membrane was calculated according to the Gouy-Chapman equation^38,39^, as Eq. (3):

3 $$|{{{{{\rm{surface}}}}}}\;{{{{{\rm{charge}}}}}}\;{{{{{\rm{density}}}}}} |=\varepsilon \kappa {{{{{\rm{\xi }}}}}}\frac{\sinh \left(\frac{F{\xi }}{2{RT}}\right)}{\frac{F{\xi }}{2{RT}}}$$

where ε is permittivity (6.933 × 10^−10^ F m^−1^), ${\kappa }^{-1}={\left(\frac{\varepsilon {RT}}{2{F}^{2}c}\right)}^{1/2}$ is Debye length (nm), ξ is surface zeta potential (mV), *F* is Faraday constant (96485 C mol^−1^), *R* is gas constant (8.314 J mol^−1^ K^−1^), *T* is the absolute temperature (298 K). *c* is the bulk concentration.

The surface zeta potential of membrane was tested in 1 mM KCl solution.

### Water transfer across membranes

The water transfer behavior across membranes was investigated by valuing the change volumes of negative and positive electrolytes during the battery cycling. The graduated cylinders were utilized as an electrolyte tank for measuring volume change. The initial volume of both sides was 80 mL and the corresponding volumes were recorded at the different cycle numbers.

### The [Fe(CN)_6_]^4-^ permeability test

The permeable K_4_[Fe(CN)_6_] solution consists of 0.4 mol L^−1^ K_4_[Fe(CN)_6_]·3H_2_O and 3 mol L^−1^ NaOH. The K_2_SO_4_ solution on another side consists of 0.4 mol L^−1^ K_2_SO_4_ and 3 mol L^−1^ NaOH. The [Fe(CN)_6_]^4-^ permeability was measured by a customized H-type diffusion cell. The left chamber of the diffusion cell was filled with 80 mL prepared K_4_[Fe(CN)_6_] solution, while the right chamber was filled with 80 mL K_2_SO_4_ solution. The concentration of [Fe(CN)_6_]^4-^ in the right chamber was determined by a UV-vis spectrometer (PERSEE TU-1901) every 0.25 hours.

### Full battery performance

An alkaline zinc-iron flow battery (AZIFB) was assembled with a membrane of a 3 × 3 cm^2^ effective area. The graphite plate and carbon felt were serve as the current collector and the electrode respectively. The battery is assembled in the sequence of graphite plate, carbon felt, membrane, carbon felt and graphite plate, and all the components are close together. The carbon felt was purchased from Liaoyang Jingu Carbide Co., Ltd. The positive electrolyte contains 3 mol L^−1^ KOH and 0.4 mol L^−1^ Na_4_[Fe(CN)_6_]·12H_2_O. The negative electrolyte contains 3.8 mol L^−1^ NaOH and 0.2 mol L^−1^ ZnO. The electrolytes were circulated through the electrodes operated with two magnetic pumps. During the charge-discharge cycling tests, the battery was charged to different charge times according to charge capacity, and the protect charge voltage is 2.3 V. Then it was discharged to a cut-off voltage of 0.1 V with Arbin BT 2000 or Neware battery test system (CT-4008T-5V12A-204n-F, Shenzhen, China).

### Symmetrical flow battery performance

Similar to full battery assembling, an asymmetrical flow battery was assembled with a membrane of 9 (3×3) cm^2^ effective area, excepting a polished Zn foil and a carbon felt serve as the anode. The negative and positive electrolytes were identical containing 3.8 mol L^−1^ NaOH and 0.2 mol L^−1^ ZnO. At the initial test stage, Zn metal lost electrons and dissolved into the positive electrolyte while the Zn(OH)_4_^2-^ was reduced to Zn metal plated on the negative electrode.

### DFT calculation

All calculations were performed using density functional theory (DFT) with Gaussian 16^41^ package. Firstly, the PBE0^42^ hybrid functional at def2-SVP^43^ level of basis set including the atom-pairwise dispersion (DFT-D3) correction^44,45^ with Becke–Johnson (BJ) damping^46^, was applied for geometry optimization. The implicit universal water solvation model based on solute electron density (SMD)^47^ was applied in all calculations. Secondly, the optimized structures were checked by vibrational frequency analysis^48^ at the same calculation level to ensure they were on the local minima of the potential energy surface. Thirdly, a single-point calculation for each structure was carried out at PBE0/def2-TZVP^43^ also including the DFT-D3(BJ) correction. Finally, the Gibbs Free Energy of each component was the sum of single-point energy and thermal correction to Gibbs Free Energy.

### Molecular dynamics (MD) simulations

Regular molecular dynamic (MD) simulations were applied to study the structures of the PBI membranes and PBI-metal ion interactions on the molecular level. Based on results from regular MD simulations, the metal ion-restrained strategy was further used to study the structures of metal-doped PBI membranes. Double-layer systems were constructed to study the K^+^ transport in metal-restrained PBI membranes.

#### Model preparation

A PBI chain was built with five 5,5’-bi[1H-benzimidazole] and six oxybis(4,1-phenylene) groups, then extended to an 8 × 1 × 5 units with an 11-Å intra-chain gap and an initial box of 88 × 85 × 55 Å^3^. The PBI membrane was solvated in TIP3P water molecules in a periodic box using LEaP in AMBER18 package^49^ in which the membrane extends in the XY-plane with water molecules filled above and beneath. For systems #2–4, 0.1 M ZnCl_2_, CrCl_3_, or FeCl_3_ were added to the system. Based on results from regular MD simulations, metal ion-restrained PBI systems were simulated in later systems #5–10. The lower concentration (0.02~0.03 M) of CrCl_3_ was used in later systems like #5 and #8, since nucleation tendency was observed in 0.1 M CrCl_3_. Addition of 0.15 M KCl was added to explore the K^+^ transport in double-layer systems #8–10. The 12-6-4 LJ-type nonbonded model^50^ was applied for the divalent and trivalent metal ions.

#### Double-layer systems

To evaluate the K^+^ transport in metal-restrained PBI membranes, double-layer systems (illustrated in Supplementary Fig. 17a) were constructed from the well-equilibrated single-layer membrane systems. We constructed the double-layer systems (Fig. 3) with two aqueous compartments in which ion concentration difference was generated by swapping K^+^ and/or Cl^−^ in one compartment with water molecules in the other^51^. The coordinates of a single-layer system were translated along the z-axis and then merged with another single-layer system as a double-layer system with two aqueous compartments using the TCL scripts implemented in VMD^52^ and further processed with ParmEd in AMBER18^49^. The double-layer systems were relaxed in ~100 ns MD simulation under NVT ensemble (298 Kelvin, 1 bar, Langevin dynamics thermostat) to reach equilibrated states, then went through a series of ion-water swapping and MD relaxing processes to study the ion transport in Zn-, Cr-, and Fe- PBI systems. In the ion-water swapping protocol^51^, we exchange the coordinates of K^+^ or Cl^−^ in one aqueous compartment with water molecules in the other compartment. We defined the exchange regions as the water regions 5~10 Å away from the PBI membrane. The swapping of ions and water generates ion concentration difference across the membrane. After the swapping, we relaxed the double-layer system in an 80-ns MD simulation with the same NVT ensemble, during which we record the K^+^ or Cl^−^ trajectories across the membrane. We simulated the ion-water swapping and MD relaxing process 20 times. A summary of simulation systems is provided in Supplementary Table 5.

#### Simulation Setup

All simulations were performed with the general AMBER force field (GAFF)^53^. In the polymer preparation, the force field parameters for each monomer inside the polymer system were computed at the HF/6-31 G* level of theory by performing Gaussian g09 calculations. The antechamber was used to assign the GAFF parameters, calculate the restricted electrostatic potential (RESP) under Gaussian g09 and create prepi files for the 5,5’-bi[1H-benzimidazole] and oxybis(4,1-phenylene) groups, respectively. Terminal oxybis(4,1-phenylene) group capped with hydrogen was also prepared. Then, LEaP was used to generate the polymer system by reading the prepi files and PDB files. Each single-layer system went through energy minimization, 2 ns isothermal-isovolumetric (NVT), and isothermal-isobaric ensemble with semi-isotropic pressure coupling (NPγT) equilibration scheme with position restraint on the membrane. Then, the system continued to relax without restraint under NPγT ensemble using the AMBER18 package^49^ with GPU acceleration. The NPγT ensemble was set with 298 Kelvin, 1 bar, Langevin dynamics thermostat, and semi-isotropic Monte Carlo barostat and a time step of 2 fs. All lengths of bonds to hydrogen atoms in the polymer were constrained with SHAKE. The particle mesh Ewald (PME) technique was used for electrostatic calculations. The van der Waals and short-range electrostatics were cut off at 12.0 Å with a switch at 10.0 Å. Data analysis was performed with either “cpptraj”^54^ or TCL scripts implemented in VMD^52^ and further processed and plotted using matplotlib^55^. Structural features were shown by Pymol (Schrödinger, LLC).

### Reporting summary

Further information on research design is available in the Nature Portfolio Reporting Summary linked to this article.

## Supplementary information

Supplementary Information

Peer Review File

Reporting Summary

Description of Additional Supplementary Files

Supplementary Movie 1

## Data Availability

The authors declare that all the relevant data are available from the corresponding author upon request. The source data of Fig. 1g, Fig. 3a, Fig. 5d are provided as a Source Data file. Source data are provided with this paper.

## Source data

Source Data
