# Supplementary material for: Metal-coordinated polybenzimidazole membranes with preferential K+ transport
Source: Nat Commun. 2023 Mar 1;14:1149. doi: 10.1038/s41467-023-36711-w (PMC9975182; doi:10.1038/s41467-023-36711-w)
Supplement: Supplementary file 1 — Supplementary Information [file 41467_2023_36711_MOESM1_ESM.pdf]

Supplementary Information for

Metal-coordinated polybenzimidazole membranes with preferential K^+ transport

by Wu et al.

Supplementary Table 1 The H_2 flux and the physical properties of membranes.

Membranes	H_2 flux (cm^3/min)	Thickness (μm)	Area resistance in 3M KOH (Ω cm^2)	Tensile strength (Mpa)
Zn-PBI	0.0018	35	0.59	52.89
Cr-PBI	0.0193	40	0.78	32.66
Fe-PBI	0.0246	25	0.83	79.25
PBI-D	-	70	1.30	9.57

Supplementary Table 2 The reduced modulus (Er) and hardness (H) data of PBI-D and M-PBI in nanoindentation.

Membranes	Test position	Reduced modulus Er (GPa)	Hardness H (GPa)
PBI-D	Top surface	0.21	0.01
Fe-PBI	Top surface	4.77	0.33
Zn-PBI	Crest of the top surface	4.79	0.32
	Trough of the top surface	5.75	0.42
	Bottom surface	5.46	0.40
Cr-PBI	Top surface	2.60	0.07
	Bottom surface	5.16	0.36

Relatively speaking, the Er of the bottom was higher than that of the upper of Zn-PBI and Cr-PBI. This difference in Er may be linked to the shorter contact time of the bottom layer with DMF during membrane preparation.

Supplementary Table 3 The bulk chemical compositions of M-PBI according to ICP-MS and ONH elemental analyzer.

Membranes	Molality of Metal (M) (%)	Molality of Nitrogen (N) (%)	Molar ratio of M: N (Rm)	The approximate number of metal ions attached to 2 PBI segment repetitive units
Fe-PBI	0.1274	0.6006	1:4.71	2
Zn-PBI	0.0819	0.5504	1:6.72	1
Cr-PBI	0.0066	0.6525	1:99.48	0.08

Please note the molality of metal elements was obtained from ICP while the molality of N was obtained from ONH elemental analyzer.

Supplementary Table 4 Structural parameters of Fe-PBI, Zn-PBI, Cr-PBI and their corresponding foil references extracted from the EXAFS fitting.

Membranes	Bond type	CN*	R(Å)	σ^2 (10^{-3}Å^2)**	R factor
Zn foil	Zn-Zn	6	2.65±0.02	5.6±1.7	0.002
		6	2.76±0.02	8.9±1.8	
Zn-PBI	Zn-N	3.7±0.4	2.03±0.02	2.7±1.6	0.020
Cr foil	Cr-Cr	8	2.49±0.01	7.1±2.4	0.012
		6	2.86±0.01	4.8±2.5	
Cr-PBI	Cr-N	6.6±0.5	2.01±0.01	3.3±0.8	0.013
Fe foil	Fe-Fe	8	2.46±0.02	5.5±0.7	0.008
		6	2.85±0.02	4.5±1.2	
Fe-PBI	Fe-N	6.7±0.5	2.02±0.02	7.9±1.3	0.013

* CN: coordination number; S_0^2 was fixed to be 0.74 for Fe, 0.84 for Zn, 0.84 for Cr

** σ^2: Debye–Waller factors

Supplementary Table 5 A summary of major MD simulations.

	system	Total atom/ H_2O No.	initial box	chain No.	N No.	Metal No.	MD time (ns)
			MD				
1	PBI	33147/7609	88×85×70	40	800	0	10423, 4918
2	PBI+Cr	33247/7609	88×85×71	40	800	25	5856
3	PBI+Fe	33247/7609	88×85×72	40	800	25	5775
4	PBI+Zn	33222/7609	88×85×73	40	800	25	5841
			metal-restrained MD				
5	PBI+Cr [a]	33163/11200	88×85×85	40	800	10	1000×3
6	PBI+Fe	33193/11068	88×85×85	40	800	25	1000×3
7	PBI+Zn	33168/11133	88×85×85	40	800	25	1000×3
double-layer systems for # 5-7							
8	PBI+Cr	102408/27164	70×85×194	80	1600	4	80×20
9	PBI+Fe	109402/29434	70×89×200	80	1600	50	80×20
10	PBI+Zn	94664/24538	70×83×184	80	1600	50	80×20

[a] only 2 Cr^{3+} were restrained to N1, and the rest moved freely.

Supplementary Table 6 The absolute energies of all substances involved in the article were sorted out in the following table. Here, the unit of energy Hartree is equal to 627.51 kcal mol^{-1}.

Substance	Absolute energy (Hartree)
Zn^{2+}	-1778.19
Cr^{3+}	-1042.11
Fe^{3+}	-1261.44
PBI	-1294.92
PBI- Zn^{2+} (pyridine N)	-3073.9867
PBI- Cr^{3+} (pyridine N)	-2338.9034
PBI- Fe^{3+} (pyridine N)	-2558.137
DMF	-248.34
DMF- Zn^{2+}	-2026.81
DMF- Cr^{3+}	-1291.23
DMF- Fe^{3+}	-1510.50

Supplementary Table 7 The interaction energies of all reactions involved in the article were calculated according to the difference between reactants and products and sorted out in the following Table.

Reaction	Interaction energy (kcal mol^{-1})
Zn^{2+} + DMF = DMF-Zn^{2+}	-173.90
Cr^{3+} + DMF = DMF-Cr^{3+}	-484.64
Fe^{3+} + DMF = DMF-Fe^{3+}	-453.75
Zn^{2+} + PBI = $PBI_{pyridineN}$-Zn^{2+}	-543.43
Cr^{3+} + PBI = $PBI_{pyridineN}$-Cr^{3+}	-1166.51
Fe^{3+} + PBI = $PBI_{pyridineN}$-Fe^{3+}	-1109.35
Zn^{2+}-DMF + PBI = $PBI_{pyridineN}$-Zn^{2+} + DMF	-369.52
Cr^{3+}-DMF + PBI = $PBI_{pyridineN}$-Cr^{3+} + DMF	-681.87
Fe^{3+}-DMF + PBI = $PBI_{pyridineN}$-Fe^{3+} + DMF	-655.60

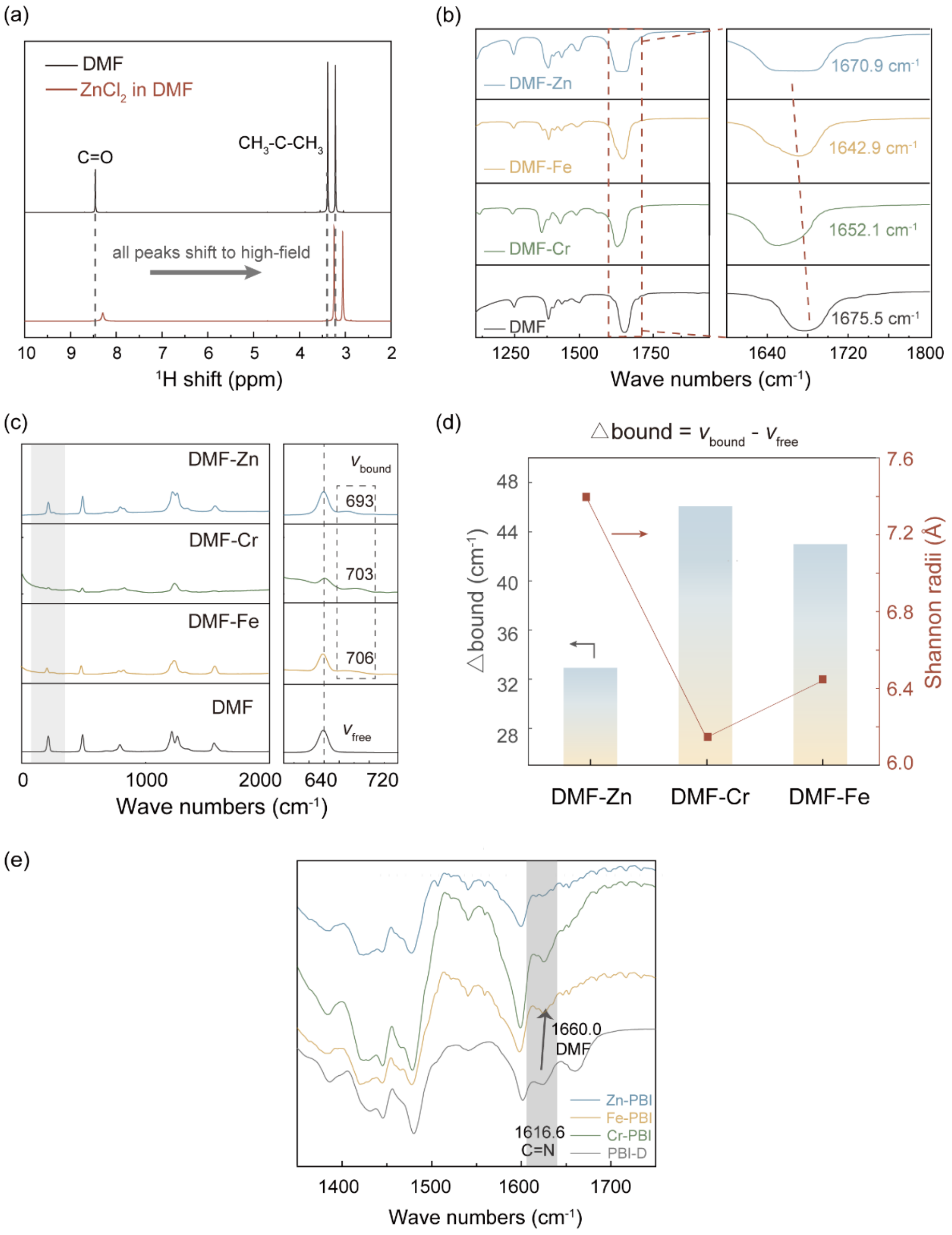

Supplementary Fig. 1 (a)-(d) The DMF solvation behavior of metal ions in Step I and (e) the metal ions coordination of PBI in Step II. The (a) ^{1}H NMR of 2 mol L^{-1} $ZnCl_2$/DMF solution and (b) FTIR of 2 mol L^{-1} MCl_x/DMF solutions. (c) The Raman characterization of 2 mol L^{-1} MCl_x-DMF solutions. (d) Raman characterization of MCl_x-DMF solutions results showed the vibration difference △bound was inversely proportional to the Shannon effective ionic radius, which indicated the weak solvation ability of Zn^{2+} by DMF in Step I. (e) The FTIR results of PBI-D and M-PBI.

When MCl_x dissolved in DMF, in Supplementary Fig. 1a, all peaks shifted to a higher field as a whole, indicating the electron density of the H nucleus increases, which may result from the

solvation behavior of metal ions. After Zn^{2+} was coordinated with C=O of DMF, the ability of O in C=O to attract electrons outside the H nucleus decreases.

Besides, this interaction between metal ions and C=O of DMF (M···O=C) was so strong that cannot be ignored, which was proved by the peak splitting[1]. As manifested in Supplementary Fig. 1b, after adding metal chloride salts (MCl_x) in DMF, the C=O stretching vibration of DMF shifted to lower wavenumbers, and resultant peak splitting leaded to peak broadening. Derived from Hooke's law, when the polarity of C=O was increased, that was the electron cloud deviated from the geometric center of the bond, and the double bond became weaker and moved towards the lower wavenumber. Peak splitting resulted from the strong coupling. When two identical C=O were connected to the metal ions, stretching vibration coupling was produced, and the vibration absorption peak thus split, forming double peaks. The splitting between the two peaks indicated strong coupling[2].

Raman was performed to further explain the solvation ability. As shown in Supplementary Fig. 1c, In MCl_x-DMF solutions, the formation of M···O=C gave rise to a free solvent band ($C=O_{free}$) shift together with a new-emergence bound solvent band ($C=O_{bound}$)[3]. The frequency of $C=O_{bound}$ had closely linked to metal ions that the vibration difference △bound (= $\nu_{bond} - \nu_{free}$) was inversely proportional to the Shannon effective ionic radius[4]. More importantly, the greater gap in △bound standed for the stronger binding of M···O=C. The greater gap in △bound (= $\nu_{bond} - \nu_{free}$) standed for the stronger binding of M···O=C (Supplementary Fig. 1d). Raman further explained that Zn^{2+} was more capable of freeing from DMF than Fe^{3+} and Cr^{3+}, which was consistent with theoretical calculation results.

Via FTIR spectra (Supplementary Fig. 1e), we demonstrated the priority of –N= as a binding site. Compared with PBI-D, the C=N stretching vibration of M-PBI was blue-shifted, which indicated the coordination between metal ions and –N= of PBI chains.

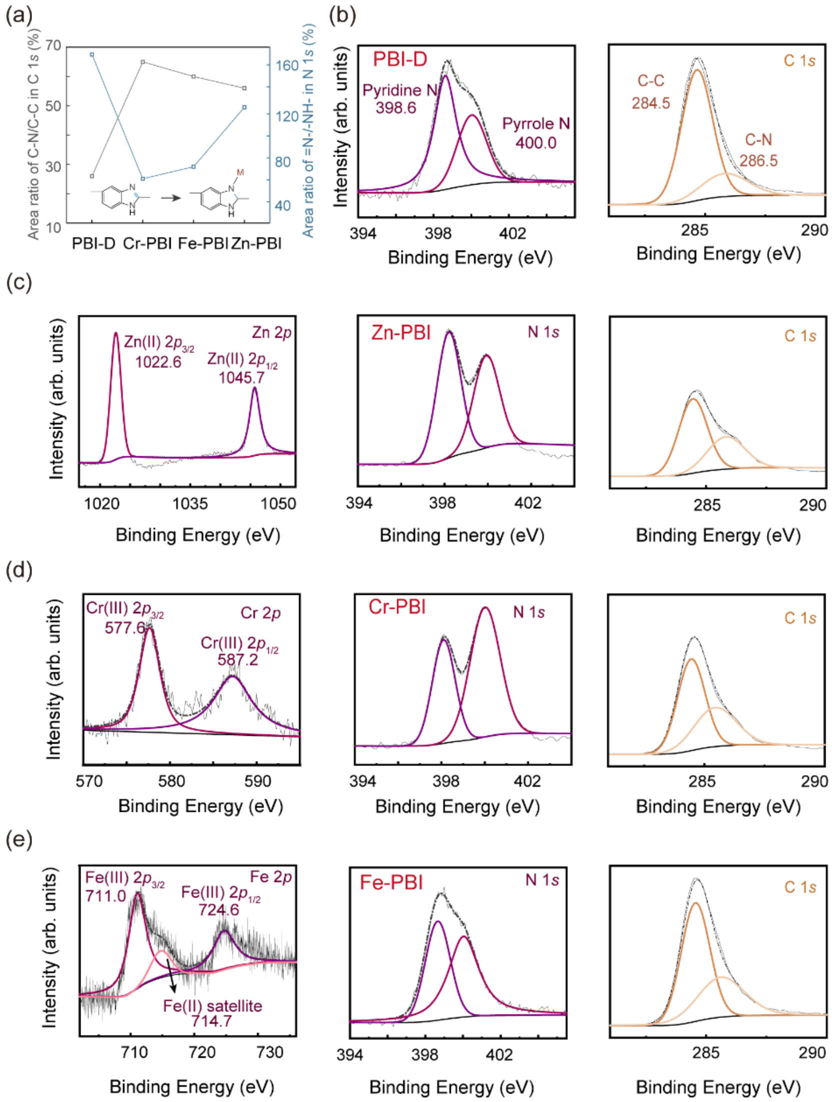

Supplementary Fig. 2 The results of XPS characterization on PBI-D and M-PBI. (a) The XPS peak area ratio of C-N/C-C in C 1*s* spectra and =N-/-NH- in N 1*s* spectra in different membranes. (b) N 1*s* spectrum, C 1*s* spectrum of PBI-D. (c) N 1*s* spectrum, C 1*s* spectrum, and Zn 2*p* spectrum of Zn-PBI (d) N 1*s* spectrum, C 1*s* spectrum, and Cr 2*p* spectrum of Cr-PBI. (e) N 1*s* spectrum, C 1*s* spectrum, and Fe 2*p* spectrum of Fe-PBI.

According to the C 1*s* spectra and N 1*s* spectra, the two peaks at 284.5 eV and 285.6 eV are indexed to C-C and C-N respectively, and two peaks around 398.6 eV and 400.0 eV are indexed to –N= and -NH- respectively[5]. The risen peak area ratio of C-N/C-C after coordination revealed that part of C=N converts to C-N in the imidazole ring. Moreover, the –N= peak area decreased compared with that of -NH- in the N 1*s* spectra, likewise suggesting the transformation of C=N to C-N[6].

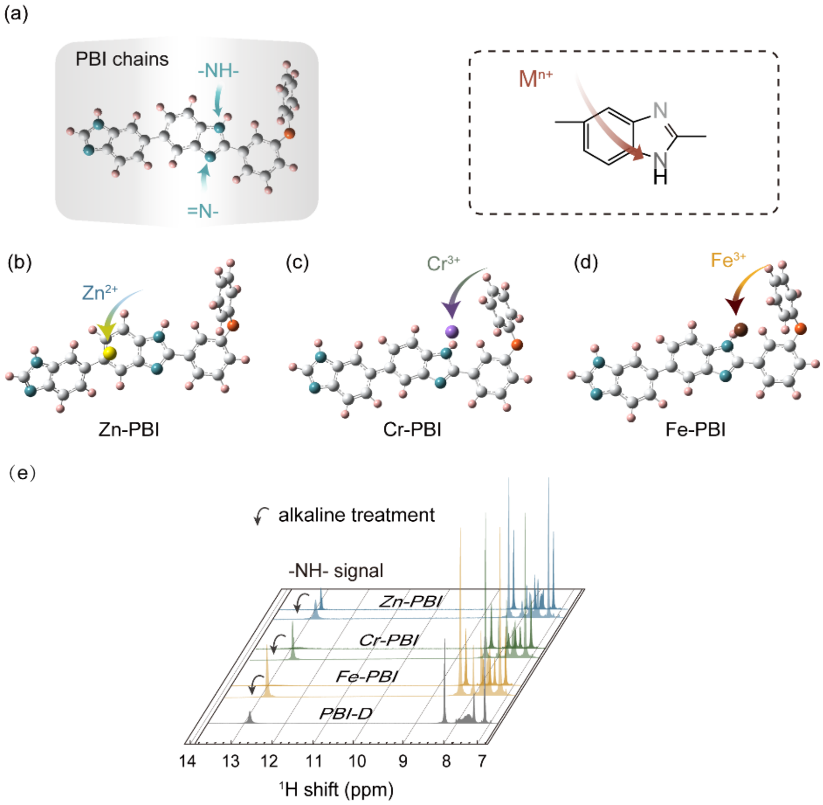

Supplementary Fig. 3 Demonstration of the –N= in chains of PBI as prevailed coordination site with metal ions. (a) The optimized coordination structures that various metal ions were controlled close to -NH- of PBI chains deliberately at the start of the structure simulation for (b) Zn-PBI, (c) Cr-PBI, and (d) Fe-PBI. (e) The ^{1}H NMR results before and after treating membranes with 3 mol L^{-1} KOH.

In Supplementary Fig. 3b-d, all metal ions were finally far away from –NH- especially Zn^{2+}. Note that Cr^{3+} and Fe^{3+} can somehow interacted with –NH- to some extent, crediting to their greater ability to donate electrons.Whether pyrrole N (-NH-) or pyridine N (–N=) of the imidazole ring was involved in coordination is controversial so far[7,8,9]. The view of –N= as the ligand site prevailed, since metal ions generally belonged to acids and tended to interact with base sites (that is –N=), according to Pearson's hard-soft acids-bases (HSAB) principle[10,11]. When metal ions were deliberately controlled close to -NH- of PBI chains at the start of the simulation. The optimized coordination structure intuitively showed that all metal ions were finally far away from -NH- especially Zn^{2+}.

In Supplementary Fig. 3e,The signal of -NH- in Cr-PBI and Fe-PBI decreased significantly. It indicated that highly reactive metal ions such as Cr^{3+} and Fe^{3+} can also interact with -NH- to some extent. However, the coordination interaction was not strong, because the signal of -NH- was redetected after treating membranes with strong alkaline.

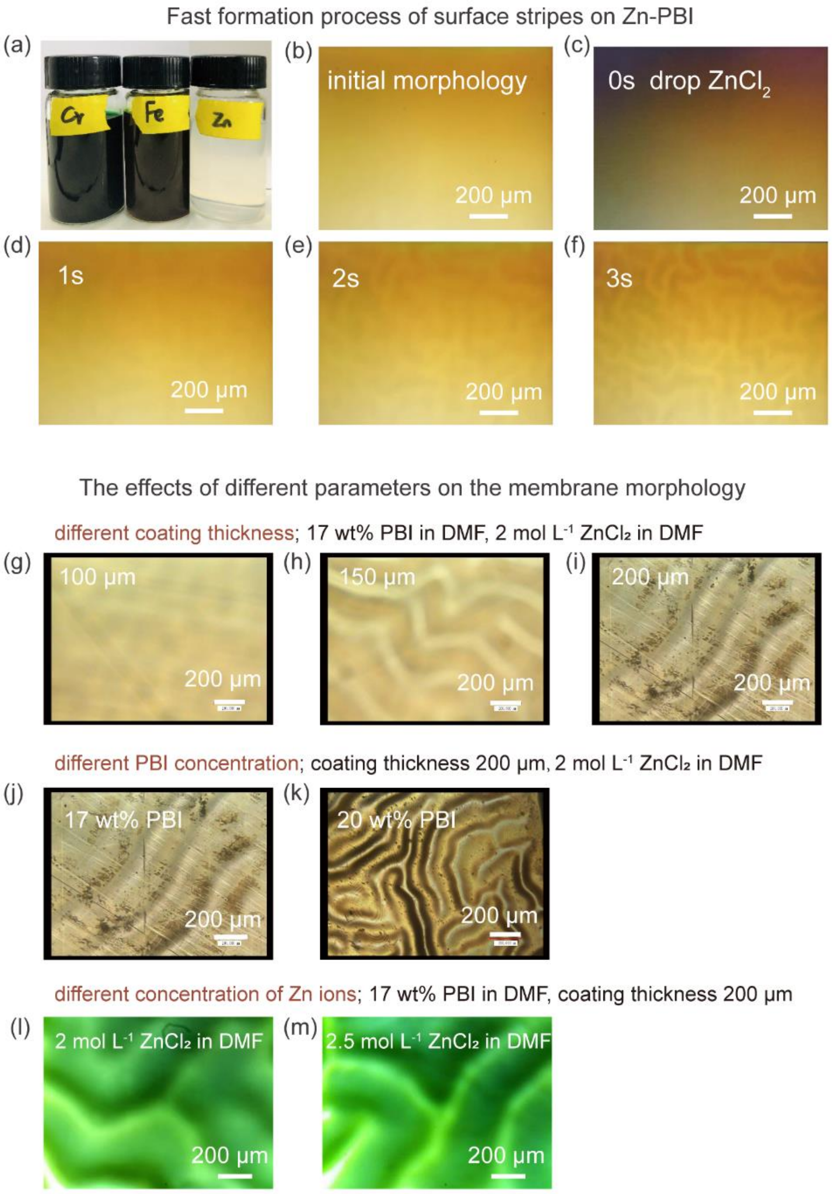

Supplementary Fig. 4 (a)-(f) The fast formation process of surface stripes on Zn-PBI. The effects of (g)-(i) coating thickness, (j)-(k) the concentration of polymer, and (l)-(m) the concentration of Zn ions on the membrane morphology.

It is crucial to study the processing-morphological characteristics of the membranes. We have carried out a series of experiments to research the membrane-forming parameters and we found this is a complex process. Several parameters were proved to affect the membrane morphology, including the types and concentration of metal ions, the polymer concentration, and coating thickness. Since the mentioned parameters affected the overall coordination reaction. We showed the effects of coating thickness, the concentration of polymer, and the concentration of Zn ions on the membrane morphology. In a certain reaction time, increase the concentration of metal ions and polymer, or reduce the coating thickness, the surface patterns space of membranes becomes narrower.

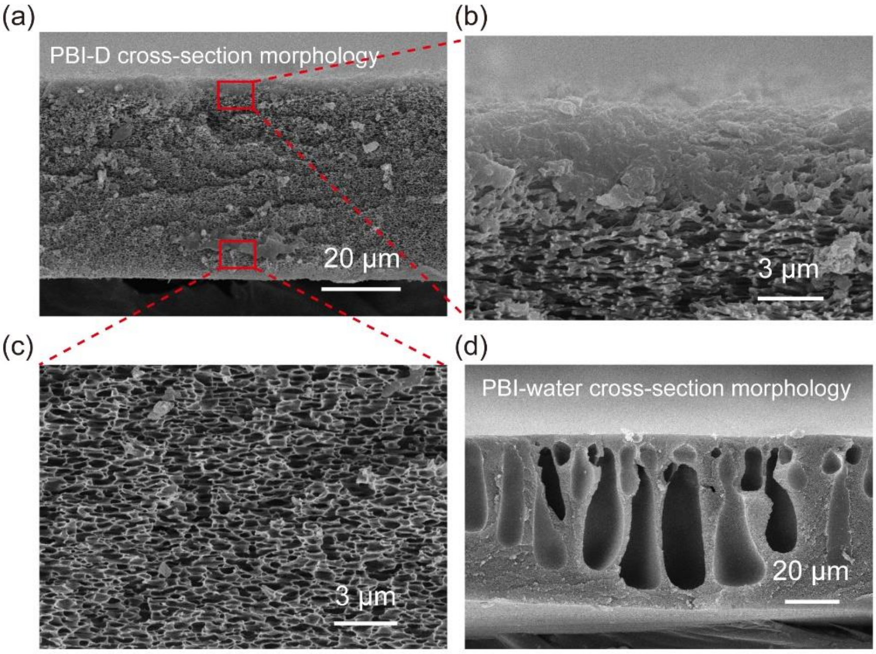

Supplementary Fig. 5 The cross-section morphology of (a)-(c) PBI-D and (d) PBI-water.

The formation mechanism is speculated that the coated PBI gel was soaked in DMF and a hydrophobic DMF layer formed. When PBI gel is reimmersed in water, it will undergo comparably slow solvent–nonsolvent exchange[12], as a result generating a relatively uniform spongy cross-section, which is different from the morphology of PBI-water formed via directly immersing PBI gel in water.

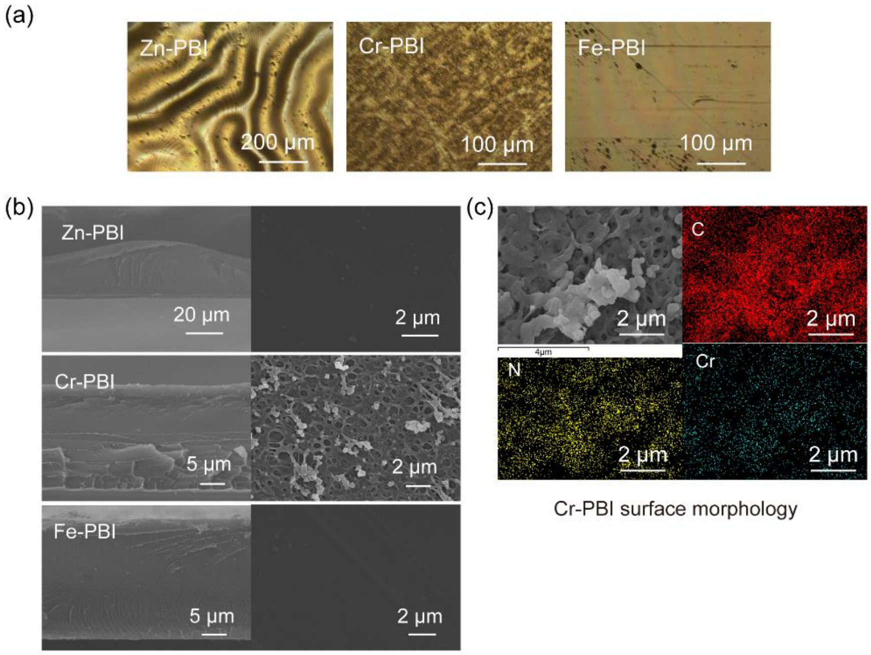

Supplementary Fig. 6 (a) The surface morphology of Zn-PBI, Cr-PBI, and Fe-PBI was observed by the ultradeep surface morphology determination microscope. (b) The cross-section and surface morphologies of Zn-PBI, Cr-PBI, and Fe-PBI. (c) The elements mapping of Cr-PBI surface.

When magnificate Cr-PBI surface by SEM, it discovered abundant protrusions and holes, which were different from dense surfaces of other counterparts. As shown in Supplementary Fig. 6c, at these prominences on the Cr-PBI surface, the C, N, and Cr elements were enriched, which may result from the top layers' aggregation during the process of membrane formation. Combined with surface holes, it was speculated that coordination reaction was intense when Cr-PBI was formed, causing a series of reaction sites to aggregate into protrusions, while the surrounding area was destroyed owing to stress.

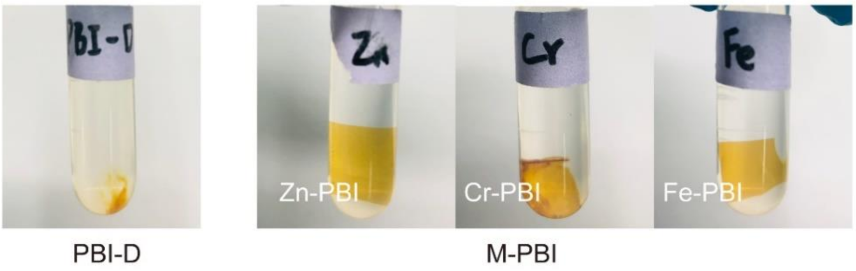

Supplementary Fig. 7 Swelling resistance test. After immersion in DMF for 2 min, PBI-D dissolved while Zn-PBI and Fe-PBI were resistant to swelling, and Cr-PBI slightly swelled. Zn-PBI exhibited better organic solvent resistance than PBI-D which was rapidly dissolved when immersed in DMF.

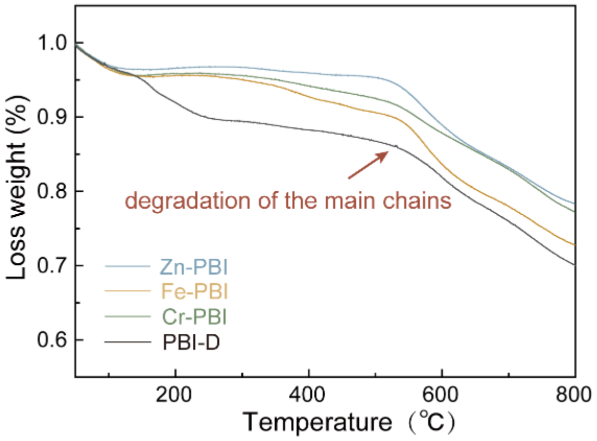

Supplementary Fig. 8 The thermal gravimetric analyzer (TGA) characterization of M-PBI and PBI-D. It can be concluded that after the PBI chain coordinated with different metal ions, M-PBI exhibited better thermal stability than PBI-D. PBI backbone degradation was observed at around 520°C[13].

Overall, after Zn^{2+} coordination, the thermal stability of Zn-PBI was superior to PBI-D, and its tensile strength was improved by 5.5 times as well.

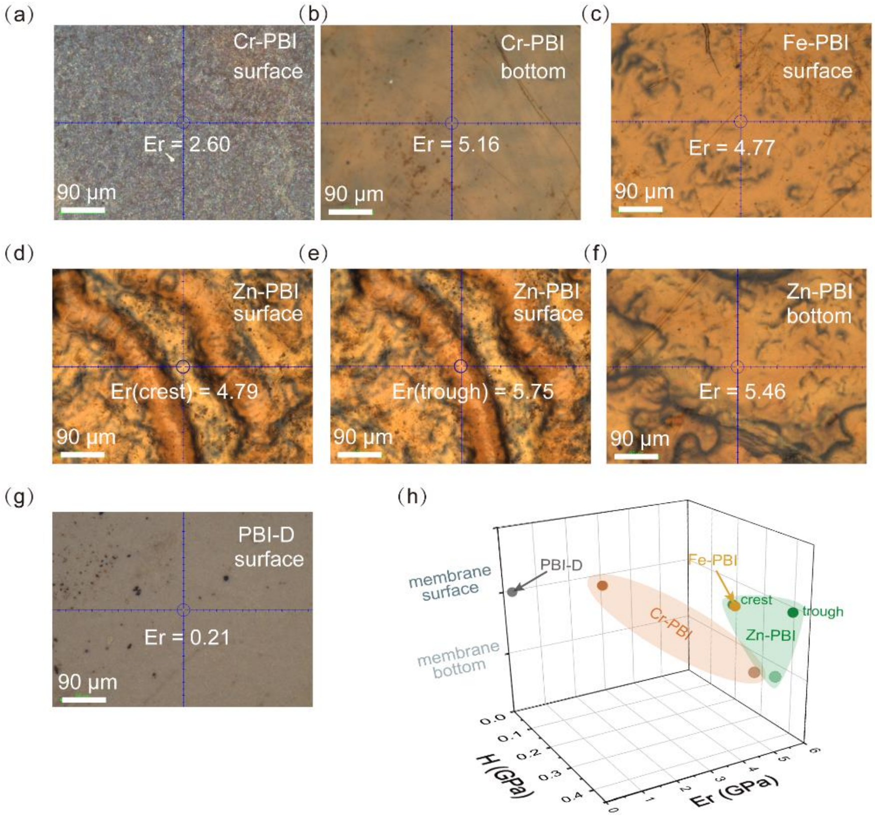

Supplementary Fig. 9 The nanoindentation was exerted to analyze the discrepancy in mechanical properties of microregions in membranes. The (a) upper surface and (b) bottom surface for Cr-PBI. (c) The upper surface for Fe-PBI. The (d)-(e) upper surface and (f) bottom surface for Zn-PBI. (g) The upper surface for PBI-D. The obtained reduced modulus (Er) and hardness (H) data were summarized in Supplementary Table 2 and compared in (h).

The coordination boosts microregional mechanical properties, that the reduced modulus (Er) and hardness (H) of Zn-PBI were over the PBI-D by nearly an order of magnitude, according to the nanoindentation. Worth noting was that stripes of Zn-PBI showed discrepant microregional mechanical properties, for surface stripes in Zn-PBI, Er increased by 20%, and H increased by 31% at trough compared with crest. It may result from the coordination-induced regional polymer segment migrations.

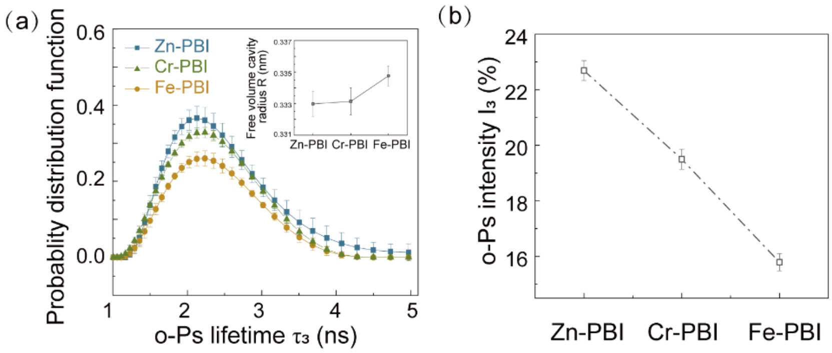

Supplementary Fig. 10 Positron annihilation lifetime (PAL) spectra of M-PBI. (a) The *o*-Ps lifetime τ_3 distribution and (b) *o*-Ps intensity I_3 were calculated. The inset showed the average free volume cavity radius (R) calculated from the *o*-Ps lifetime τ_3.

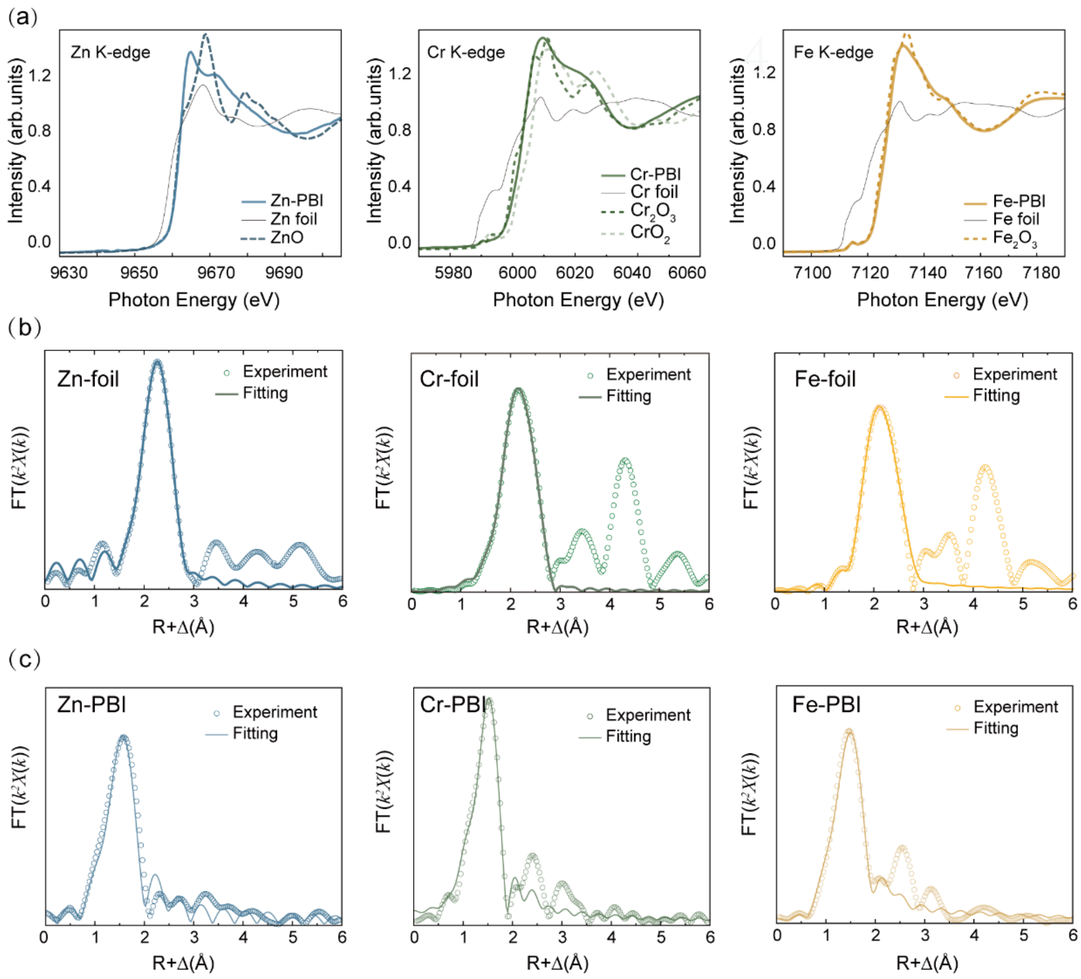

Supplementary Fig. 11 (a) Fe K-edge, Zn K-edge, and Cr K-edge XANES data of Zn-PBI, Cr-PBI, and Fe-PBI and the corresponding reference samples. Fourier transform of Fe K-edge, Zn K-edge, and Cr K-edge EXAFS data of (b) Fe-foil, Zn-foil, and Cr-foil and (c) Zn-PBI, Cr-PBI, and Fe-PBI respectively as well as the corresponding fits.

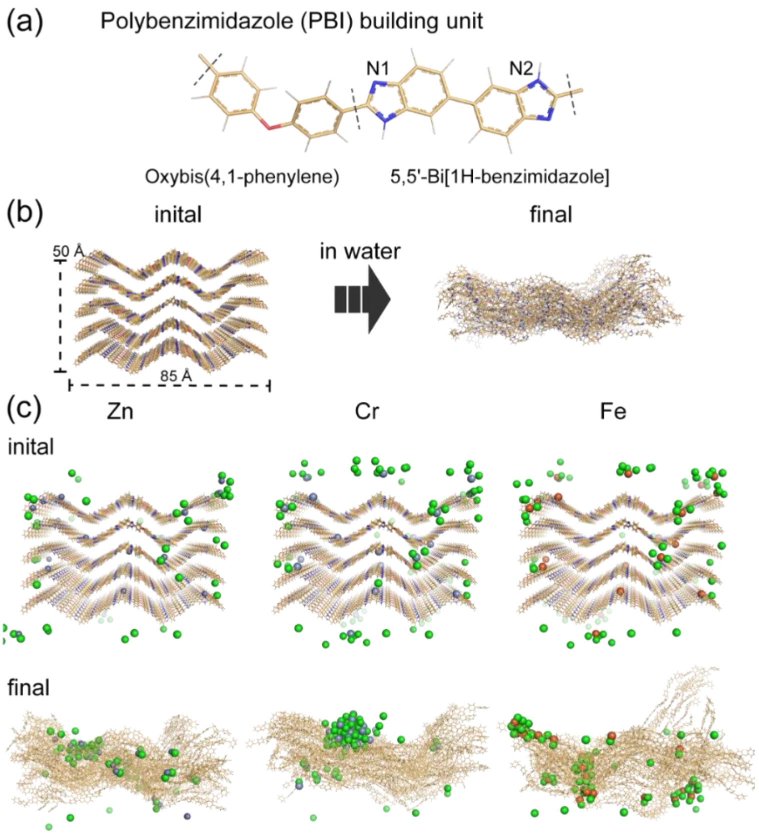

Supplementary Fig. 12 (a) The unit to build a linear PBI chain. The N without H (-N=) was labeled as N1, and the other N with H (-NH-) attached was labeled as N2. (b) The initial and final structures of PBI in microsecond MD simulations in aqueous condition (as #1 system in Supplementary Table 5). (c) The initial and final structures of PBI in 0.1 M $ZnCl_2$, $CrCl_3$, $FeCl_3$ aqueous solution in microsecond MD simulations (as #2-4 systems in Supplementary Table 5). Cl^- were shown in green sphere, Zn^{2+} and Cr^{3+} ions were shown in gray sphere, and Fe^{3+} were shown in orange sphere. The same color scheme is used in the following figures.

Microsecond MD simulations showed that the conformation of aligned PBI was similar to the nanosecond simulation study of PBI.[14] The structures and PBI-metal ion interactions reached relatively stable states (did not change much) after a few hundred nanoseconds MD simulations. Nucleation tendency was observed in $CrCl_3$ system, thus less Cr^{3+} was added in the following systems.

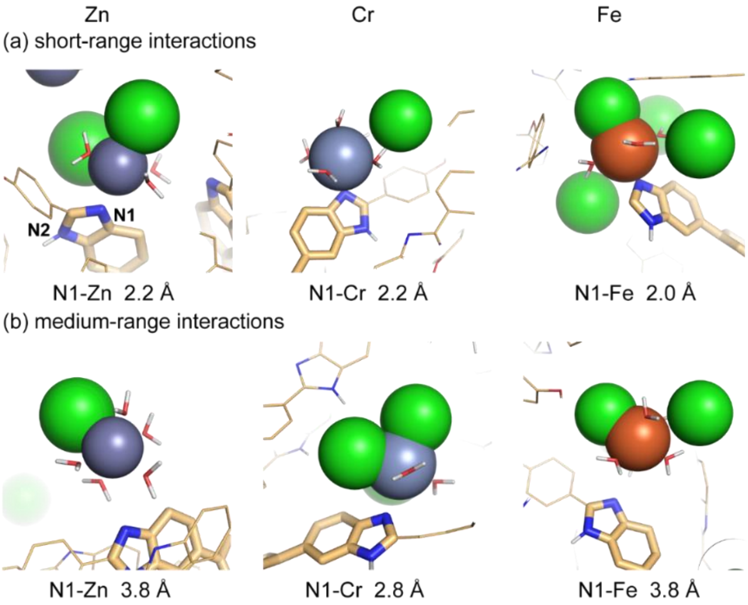

Supplementary Fig. 13 Snapshots of metal ion and PBI interactions in (a) short-range distance (~2.2 Å) and (b) medium-range distance (2.8~3.8 Å) sampled in MD simulations of systems in Supplementary Fig. 12c. Water molecules within the first solvation shell of metal ions are shown in a thin stick. The short-range distances (2.0~2.4 Å, also see Fig. 2b) for metal-PBI interactions are close to the synchrotron radiation data (Supplementary Table 4).

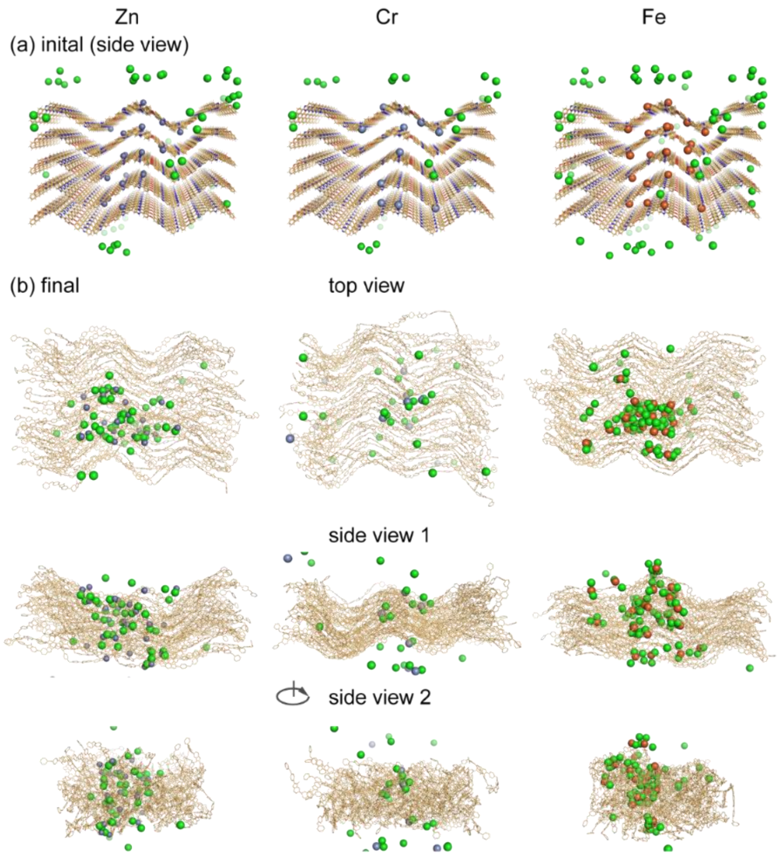

Supplementary Fig. 14 The (a) initial and (b) final structures of metal ion-restrained PBI systems in microsecond MD simulations (as #5-7 systems in Supplementary Table 5). Cl^- were shown in green sphere, Zn^{2+} and Cr^{3+} ions were shown in gray sphere, and Fe^{3+} were shown in orange sphere. The initial ion coordination for Zn^{2+} and Fe^{3+} were the same, for Cr^{3+} a few ions were removed from the configuration of Zn^{2+} and Fe^{3+} systems to generate the Cr^{3+} system.

Although we have sampled a few short-range (2.0~2.4 Å) metal-PBI interactions in regular MD simulations, the coordination number remains as one metal ion with one N1 during microsecond MD simulations. Also, the distribution of such strong interactions was relatively dispersed. To study the metal-doped PBI structure, we randomly restrained a few metal ions (as 25 Zn^{2+}, 4 Cr^{3+}, 25 Fe^{3+}, respectively) to a certain region of N1 atoms in a PBI membrane with 800 N atoms to locally mimic the metal/N ratio in PBI membrane from the ICP data (Supplementary Table 3). With the metal-N1 distance restrained at ~2.2 Å with an initial coordination number of one, the metal-restrained PBI was relaxed in water in MD simulations. During the simulation, we examined the distances between any metal ion and neighboring N1 atoms, and restrained new pairs that were within 4.5 Å. Finally, the maximum coordination number in Zn, Cr, and Fe systems increased to 4, 2, and 3, respectively (Supplementary Fig. 15).

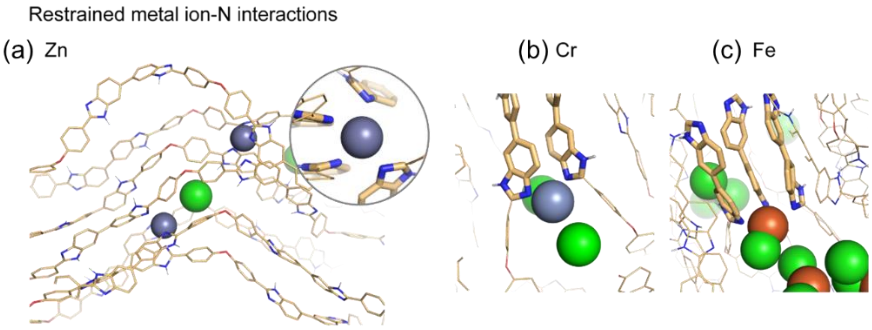

Supplementary Fig. 15 Coordinated N1 around (a) Zn, (b) Cr, (c) Fe in metal ion-restrained PBI systems in microsecond MD simulations.

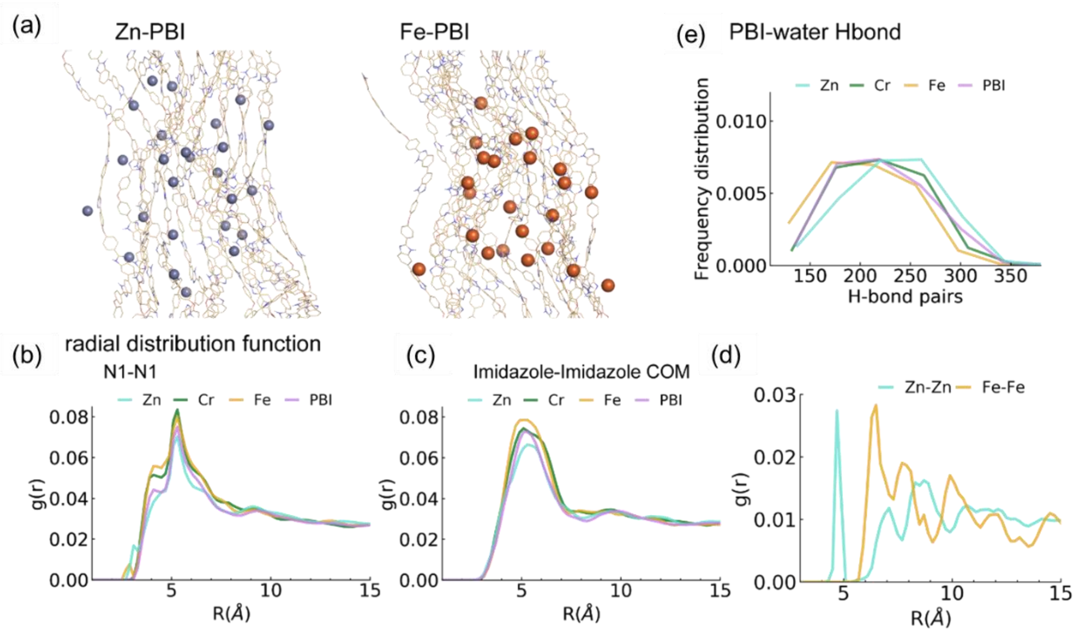

Supplementary Fig. 16 Results from single-layer metal ion-restrained PBI systems. (a) Snapshots of coordination domains from Zn-PBI and Fe-PBI simulations. The initial ion coordination setup for Zn^{2+} and Fe^{3+} were the same as shown in Supplementary Fig. 14a. Cl^{-} were not shown. Radial distribution function *g*(r) to describe the density of one group as a function of distance from another group for (b) N1-N1, (c) the center-of-mass (COM) of two imidazole, and metal ions, respectively. (d) The bulk water *g*(r) as 1 is used as reference. Metal-free system is labeled as PBI. (e) Hydrogen bonds formed between PBI (at N1, N2, and O) and water molecules in Zn-, Cr-, Fe- and metal-free PBI systems. Zn-PBI system tends to expose more polar groups to solvent.

Snapshots of coordination domains from Zn-PBI and Fe-PBI simulations in Supplementary Fig. 16a illustrate that the Zn^{2+} coordination results in more dispersed coordination domains in comparison with Fe^{3+} coordination domains.

The *g*(r) for N1-N1 and inter- imidazoles in Supplementary Fig. 16b-c show that Zn (in cyan) tends to disperse inter- imidazoles with decreased density, while Fe and Cr (orange and green lines) tends to attract inter- imidazoles with increased density at close distance (~4 Å for N1-N1 and ~5 Å for inter- imidazoles). The *g*(r) of metal ions in Zn-PBI and Fe-PBI further shows significant Fe density peaks and valley in range of 6~11 Å, which attenuates fast along increasing distance (in orange), while the Zn density fluctuates in a mild manner along increasing distance (in cyan). Thus, the Zn-doped PBI tends to expand the local conformation, while Fe- and Cr- doped contract local regions.

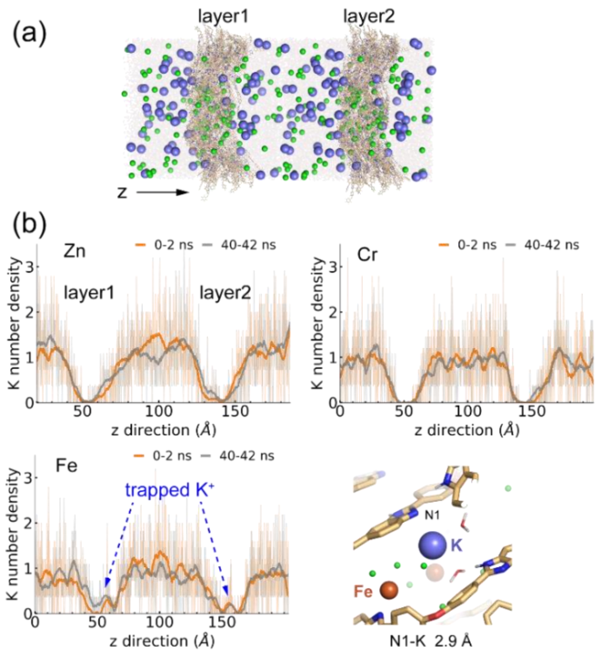

Supplementary Fig. 17 (a) The double-layer system with two aqueous compartments filled with K^+ (in blue) and Cl^- (in green). Water molecules were not shown. Ion concentration difference was generated by swapping K^+ and/or Cl^- in one compartment with water molecules in the other. (b) K^+ number density in 0.25-Å slices along the z-axis in the beginning and after 40 ns in double-layer Zn^{2+}, Cr^{3+}, and Fe^{3+} restrained PBI systems. Snapshot of trapped K^+ in Fe-PBI is displayed on the bottom right.

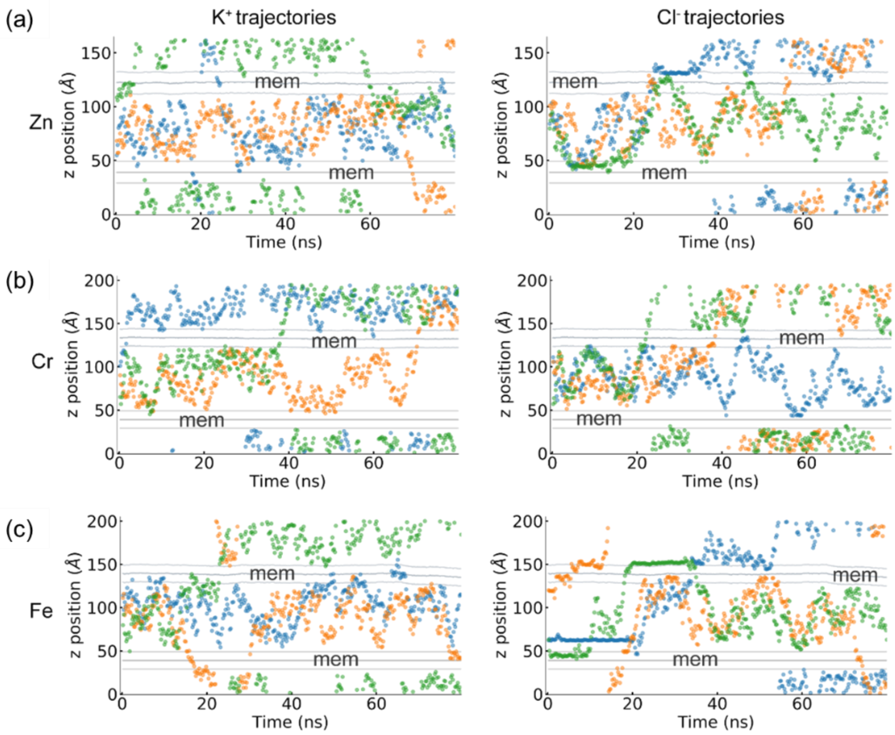

Supplementary Fig. 18 Ion transport trajectories across PBI membranes in double-layer (a) Zn^{2+}, (b) Cr^{3+}, and (c) Fe^{3+} restrained PBI systems. The z-coordination trajectories of three K^+ or Cl^- are displayed for each system with different colors. The regions of two membrane layers are labeled and marked by gray lines, the upper and lower gray lines do not strictly represent the upper surface or lower surface since the PBI membrane adopts wavy conformation.

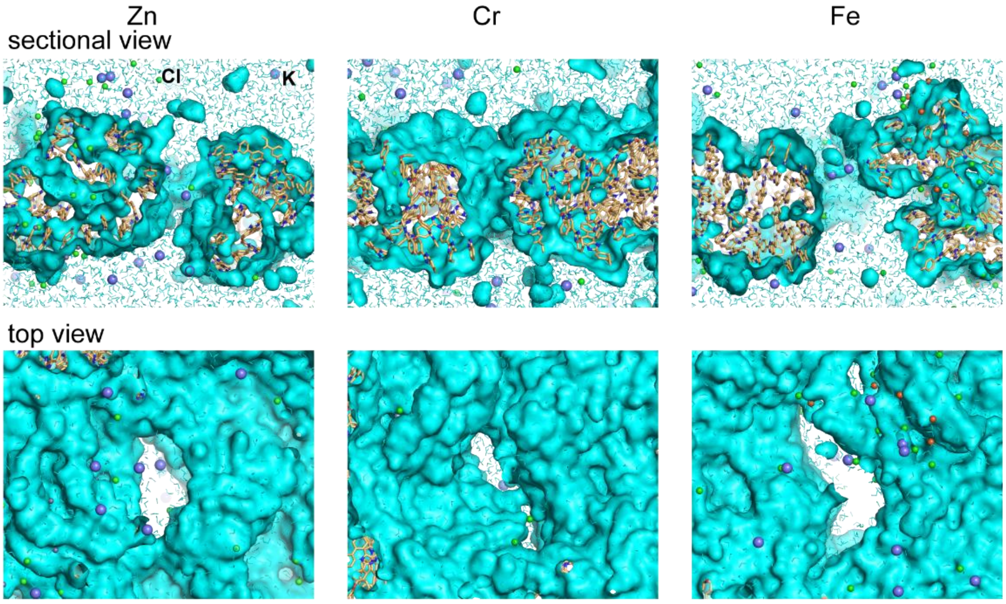

Supplementary Fig. 19 Sectional and top views of water accessible surface in double-layer Zn^{2+}, Cr^{3+}, and Fe^{3+} restrained PBI systems. Cl^- were shown in green sphere, K^+ were shown in blue sphere, and water molecules are shown in cyan surface and thin stick. By comparing the pore width in Zn^{2+}, Cr^{3+}, and Fe^{3+} restrained PBI membrane, Zn-PBI formed 10~12 Å wide pore; Fe-PBI formed elongate pore with changing width of 11~16 Å; Cr-PBI formed small pore of ~6 Å.

The sectional view shows that inside the Zn-PBI, the uniform Zn^{2+} coordination results in more dispersion of water among polymer chains, and increased hydrogen bonds formed between PBI (by polar groups N1, N2, and O) and water molecules in Zn-PBI system. While the Fe^{3+} cause different degrees of local constriction between imidazole units, which displays uneven water distribution in different region of polymer chains and makes chains over-aggregate to form larger channels.

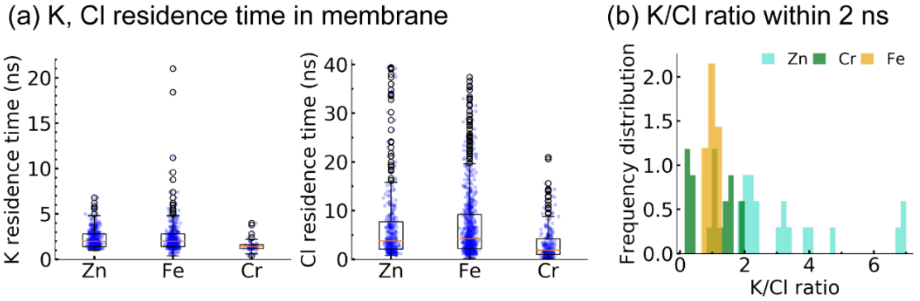

Supplementary Fig. 20 Results from double-layer metal ion-restrained PBI systems. (a) Box plot of K^+ or Cl^- residence time in Zn-, Cr-, and Fe- PBI membranes. Total K^+ transport number are 458, 431, 63 for Zn-, Cr-, and Fe- PBI, respectively; total Cl^- transport number are 347, 761, 234 for Zn-, Cr-, and Fe- PBI, respectively. (b) Frequency distribution of K/Cl ratio within 2 ns as the fast transport period in Zn-, Cr-, and Fe- PBI membranes.

The ion residence time in PBI membranes is displayed in Supplementary Fig. 21a. The K^+ residence time for Zn-PBI is in the range of 1~6.8 ns with average around 2 ns, while it becomes in wider range (0.4~21 ns) in Fe-PBI membrane. Fewer K^+ transport through Cr-PBI with residence time of 0.4~4 ns. The Cl^- residence time in Zn-PBI is in the range of 0.8~40 ns, higher than both Fe- and Cr- PBI membranes. We select the ion transport within 2 ns as the fast transport period as indicated in Supplementary Fig. 21b. K/Cl transport ratio is close to 1 for Fe-PBI system, and displays wide range of 0.14~2 for Cr-PBI system, while Zn-PBI system exhibit K/Cl transport ratio in range of 2~7.

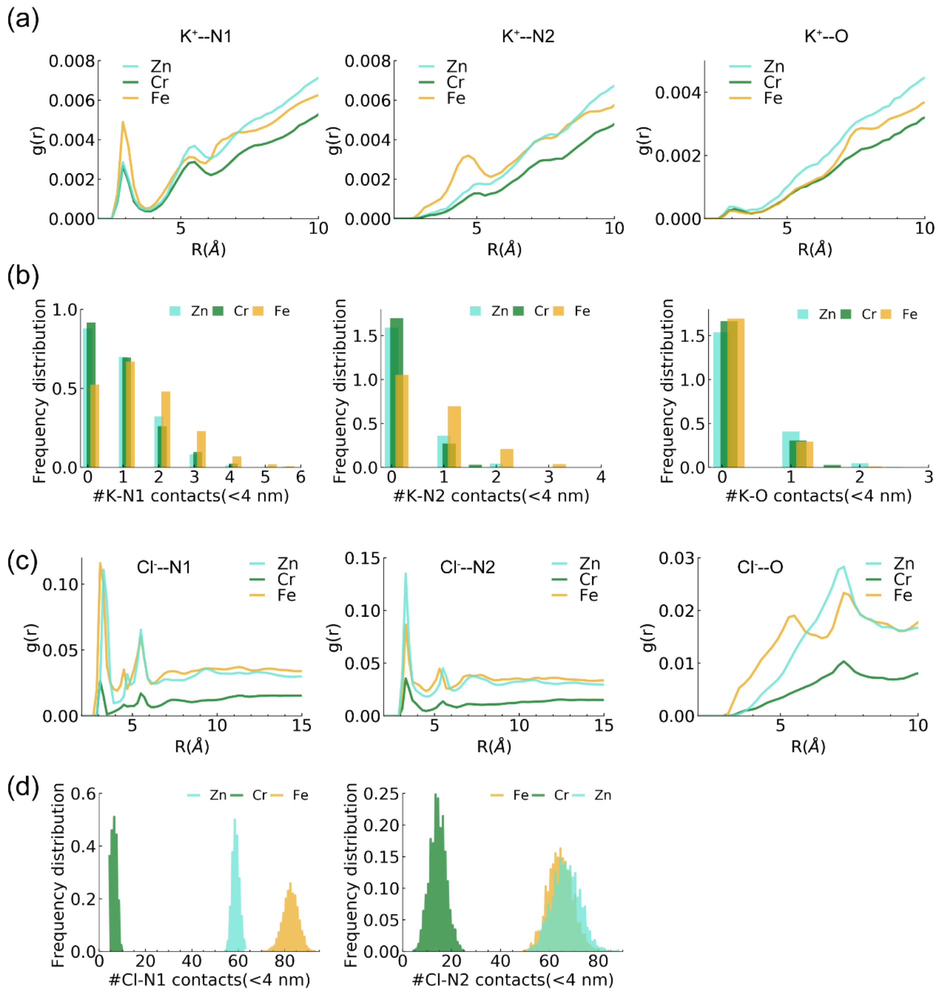

Supplementary Fig. 21 Results from double-layer Zn^{2+}, Cr^{3+}, and Fe^{3+} restrained PBI systems. (a) Radial distribution function *g*(r) of K^+ to each N1, N2, and O of PBI. (b) Frequency distribution of K^+ within 4 Å of N1, N2, and O of PBI. (c) *g*(r) of Cl^- to each N1, N2, and O of PBI. (d) Frequency distribution of Cl^- within 4 Å of N1, N2 of PBI.

Further, we observe different preference for K^+ and Cl^- in Zn-, Cr-, and Fe- PBI. The radial distribution function *g*(r) of K^+ to N1, N2, and O of PBI in Supplementary Fig. 21a show that Fe-PBI (in orange) attracts more K^+ around N1 at ~4 Å, N2 at ~4.5 Å, while Zn-PBI (in cyan) attracts more K^+ around O than neither Fe- or Cr- PBI along the radial distribution. Consistently, more K^+-N1, K^+-N2 contacts (within 4 Å) were found for Fe-PBI, and slightly more K^+-O contacts for Zn-PBI (Supplementary Fig. 21b). The *g*(r) of Cl^- to N1, N2, and O of PBI in Supplementary Fig. 21c also show that while both Zn-PBI and Fe-PBI show similar *g*(r) for Cl^- to N1 at 3.5 Å, the *g*(r) for Cl^- to N2 at 3.5 Å increases from Cr-, Fe-, to Zn- PBI, indicating higher Cl^- density around N2 in Zn-PBI. Supplementary Fig. 21d further shows that more Cl^- appear within 4 Å of N1 in Fe-PBI, while Zn-PBI attracts more Cl^- around N2 at ~3.5 Å (through -H). The difference preference for K^+ and Cl^- in Zn-, Cr-, and Fe- PBI may contribute to different K/Cl transport ratio.

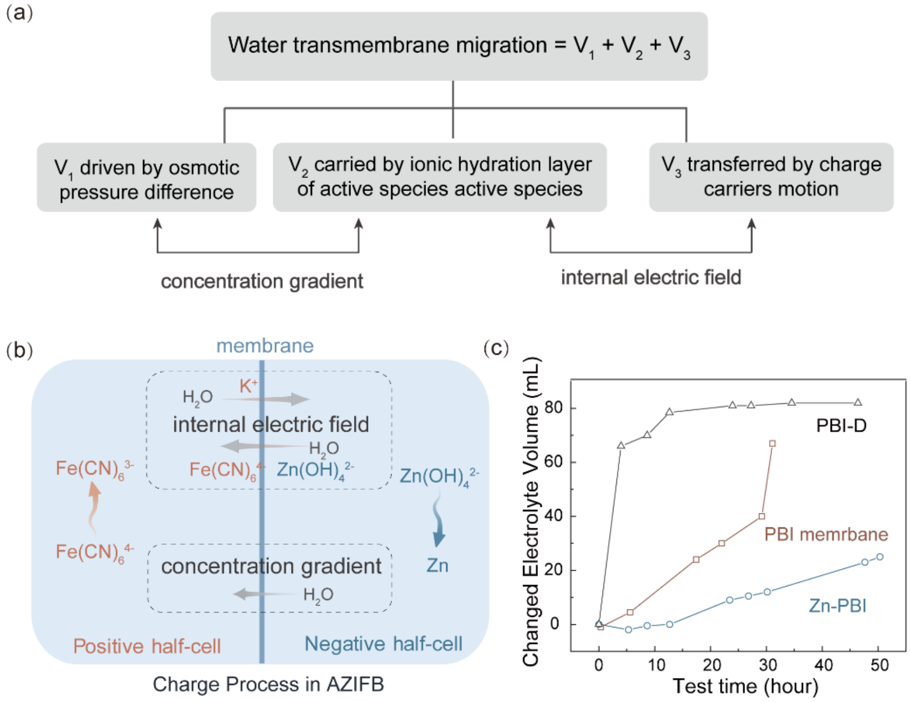

Supplementary Fig. 22 (a) Illustration of water transmembrane migration in a battery. (b) Water migration during the charge process in an AZIFB. (c) The change volumes of negative and positive electrolytes during the battery cycling when using PBI-D, PBI membrane, and Zn-PBI.

As shown in the above illustration Supplementary Fig. 22a, the direction and amount of water migration are commonly jointly driven by three processes, including osmotic pressure difference in two half-cells (V_1), water carried by ionic hydration layer of active species (V_2) in tandem with water transferred by charge carriers motion (V_3). The fundamental driving force can be considered as concentration gradient and internal electric field.

In Supplementary Fig. 22b, when an AZIFB is assembled with a cationic conduction membrane, during the charging process, charge carriers transport from the positive half-cell (PHC) to the negative half-cell (NHC) depends on the direction of the internal electric field. Likewise driven by the electric field, negative-charged active species in both PHC and NHC, that is $[Fe(CN)_6]^{4-}$ and $Zn(OH)_4^{2-}$, migrate toward PHC. Notably, $Zn(OH)_4^{2-}$ further moves to PHC impelled by the concentration gradient between the two half-cells. The motion of charge carriers and active species will transport water around them. As a result, water migrates toward the PHC and thus increases the volume of PHC.

Notably, water migration worsens battery polarization as well. It is linked to the state of charge (SOC) during the charge and discharge process. When the battery charges, the capacity of NHC is reduced. Because driven by the electric field and concentration gradient, a fraction of $Zn(OH)_4^{2-}$ mitigates to PHC and is consumed by $[Fe(CN)_6]^{4-}$. In other words, if take NHC as the standard, the SOC of PHC is lower than that of NHC. Then when the battery discharges,

there will be some deposited zinc that is unable to completely return to $Zn(OH)_4^{2-}$ and is still residual. That is, when diffusion and migration proceed in the same direction for certain active species, the impact on discharge depth will be more striking[15]. After a period of cycling, zinc cannot be discharged and thus accumulated, which will lead to insufficient discharge depth. Finally, the concentration of spendable active species in NHC decreases, hence it deteriorates the polarization.

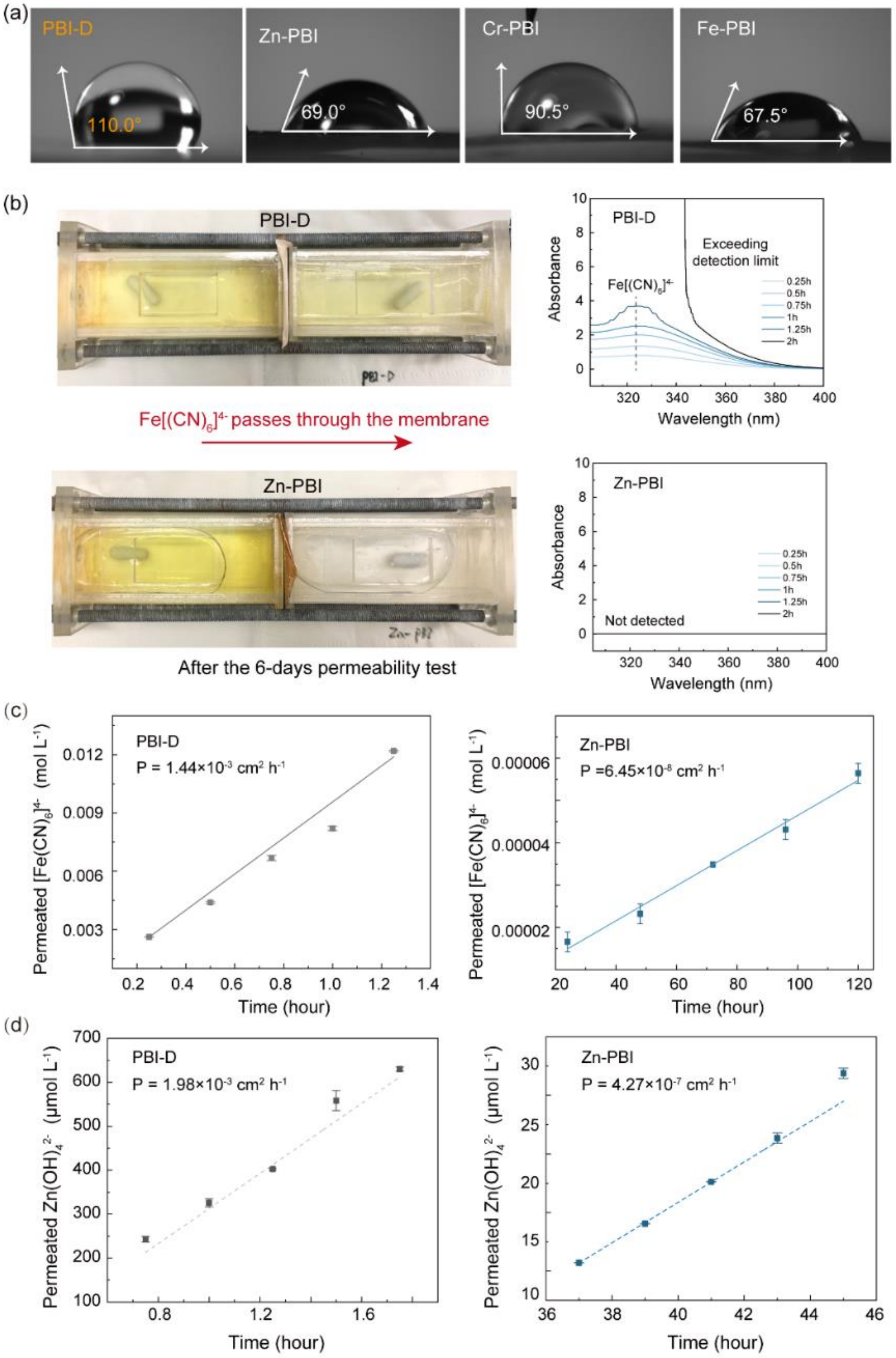

Supplementary Fig. 23 (a) The static contact angle of PBI-D and M-PBI. (b) The $[Fe(CN)_6]^{4-}$ permeability test in 6 days for PBI-D and M-PBI. (c) The $[Fe(CN)_6]^{4-}$ permeability test. (d) The $[Zn(OH)_4]^{2-}$ permeability test.

When the penetration time exceeded 2h, the absorbance on the penetration side of PBI-D would exceed the detection limit of UV-vis spectrometer. Thus we calculated the permeation concentration in 1.25h to compare Zn-PBI and PBI-D (Fig. 5c). The permeability coefficient (P) of PBI-D to iron species was 1.44×10^{-3} cm^2/h, and that of Zn-PBI was 6.45×10^{-8} cm^2/h (which was calculated from the permeability test during 120 hours).

The P of zinc species $Zn(OH)_4^{2-}$ was calculated by measuring the concentration of zinc species through the membrane with inductively coupled plasma mass spectrometry (ICP-MS).

According to the experimental results, compared with PBI-D, Zn-PBI can effectively block active species impelled by the concentration gradient.

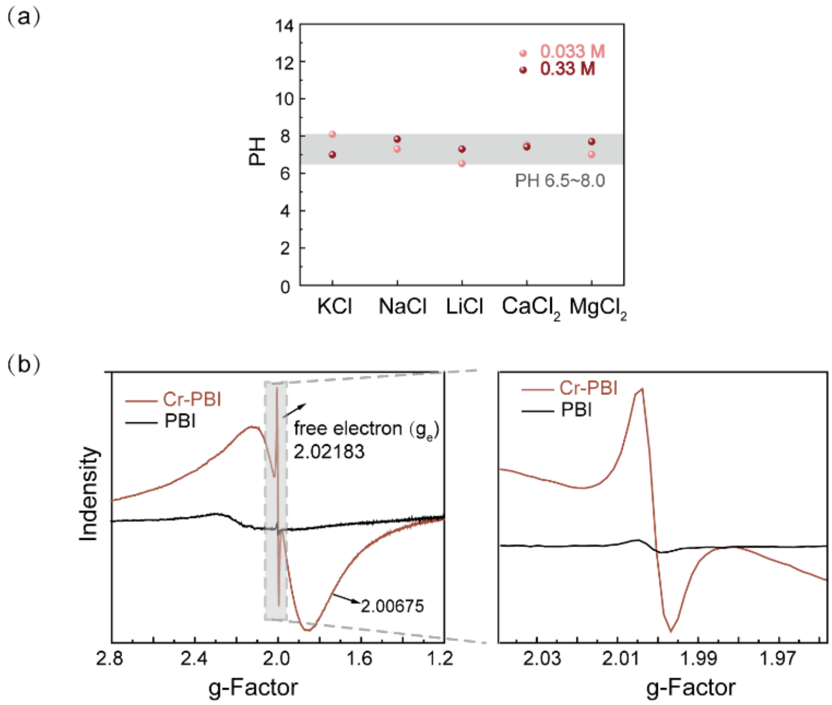

Supplementary Fig. 24 (a) The PH of different metal salt solutions. (b) The EPR results of PBI before and after Cr^{3+} coordination.

g_e = 2.02183 was credited to the free electron. PBI itself has a very weak free-electron peak. The metal coordination leads to the increase of free electron signal on PBI, which affects the charge of the PBI main chain.

The electron paramagnetic resonance (EPR) spectroscopy also implied that the electrons of metal ions delocalized over the whole PBI segments, and can counteract the negative effect of the EDL being compressed in a high-concentration solution(Supplementary Fig. 24b).

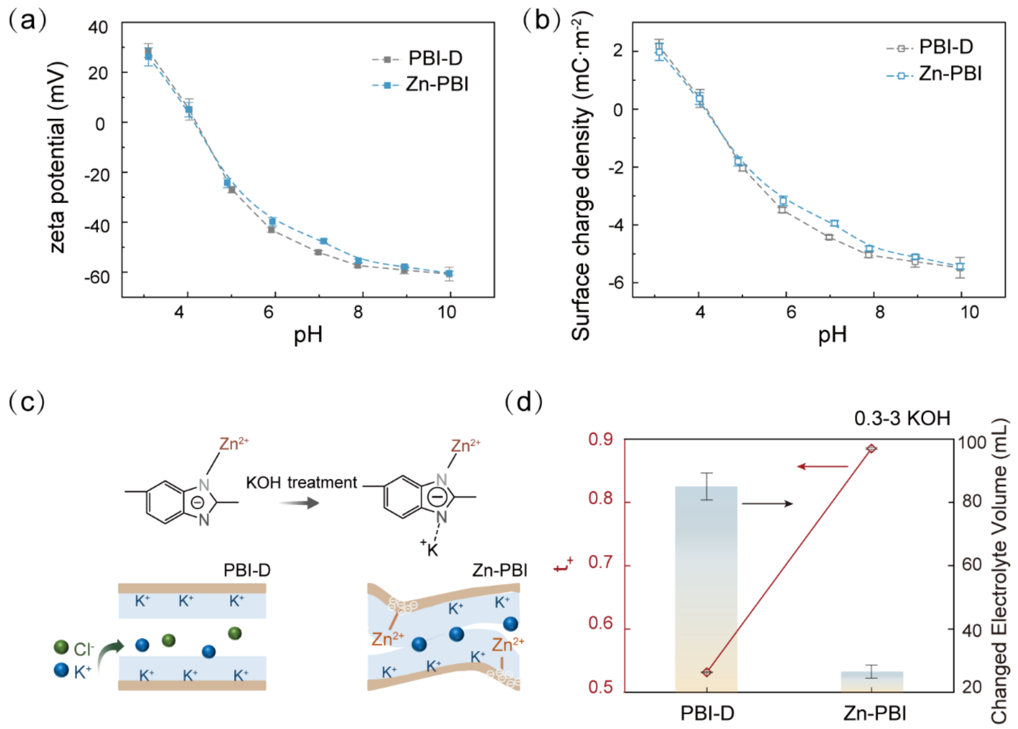

Supplementary Fig. 25 (a) The pH-dependent zeta potential and (b) surface charge density of membranes in 1 mM KCl solution. (b) Illustration of the charge change for the Zn-PBI chains when the pH changed from neutral to alkaline. Effects on ion transport through membranes when the thickness of EDL is compressed in a concentrated solution. The Debye length (λ_D), which describes the thickness of the EDL, varies inversely with the bulk ion concentration[16]. (c) The negative correlation between water migration volume and K^+ transport number (t_+). The water migration volume was recorded for PBI-D (failed after 46 hours of operation) and Zn-PBI (stop manually after 50 hours of operation). The ion transport number was tested in a KOH solution with a gradient of 0.3 and 3 mol L^{-1}.

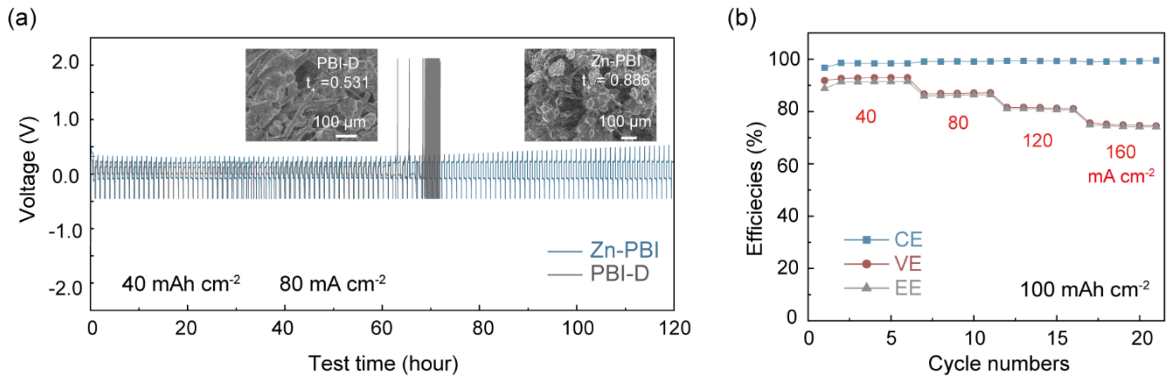

Supplementary Fig. 26 (a) The cycling performance of ZSFBs with Zn-PBI and PBI-D. (b) The performance of AZIFB assembled with Zn-PBI at the different current densities at an areal capacity of 100 mAh cm^{-2}.

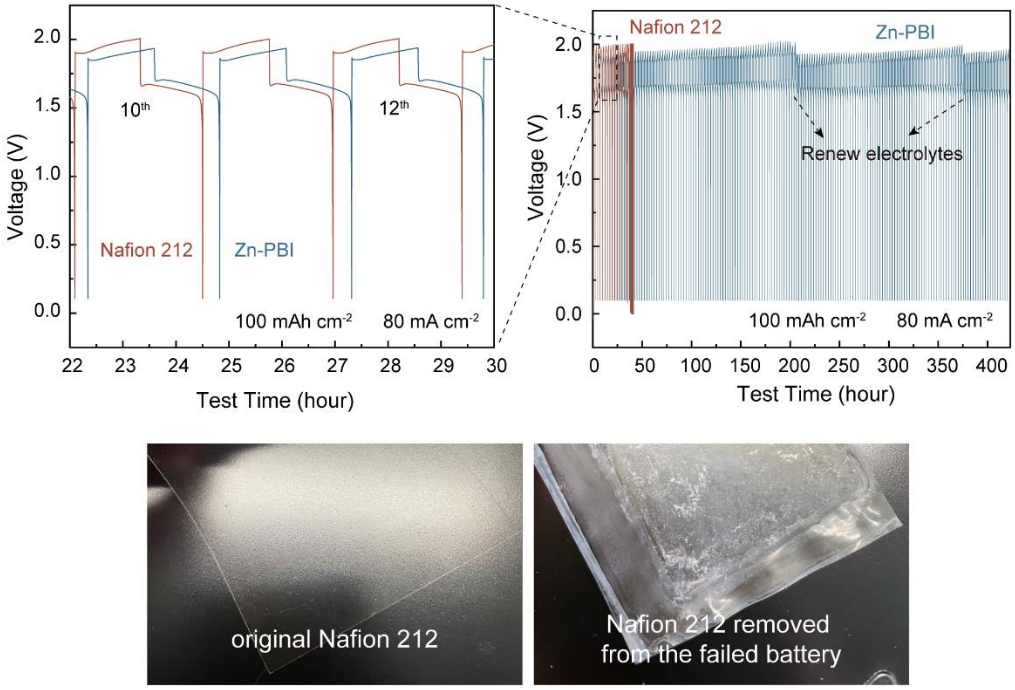

Supplementary Fig. 27 Compare the charge/discharge curves of AZIFB when using the commercial cation exchange membrane (Nafion 212) and Zn-PBI.

Assembled with Nafion 212, the AZIFB ran for less than 50 hours at the same operation condition, while the battery can stably run for more than 340 when using Zn-PBI. Referring to the previous report[17], it speculated that Nafion 212 was unable to withstand the zinc dendrite at a high areal capacity of 100 mAh cm^{-2} and a high current density of 80 mA cm^{-2}.

Enlarge charge/discharge curves of battery, it can find that the polarization of the battery is larger when Nafion 212 is used, which leads to the gradual increase of battery polarization and eventually resulting in battery failure.

Supplementary References

1. De Oliveira OA, Chagas AP, Airoldi C. Synthesis, characterization, and thermochemistry of adducts of zinc, cadmium and mercury halides with N,N-dimethylformamide. *Inorg. Chem.* **22**, 136-140 (1983).
2. Menghrajani KS, Nash GR, Barnes WL. Strong Coupling of Vibrational Modes: an Investigation using Infrared and Raman Spectroscopy. In: *13th International Conference on Fiber Optics and Photonics*). Optical Society of America (2016).
3. Kislina IS, Librovich NB, Maiorov VD. Raman spectra of complexes of HCl with DMF with a strong quasisymmetric H-bond in solutions. *Russ. Chem. Bull.* **43**, 1505-1507 (1994).
4. Umebayashi Y, Matsumoto K, Watanabe M, Ishiguro S-i. Individual solvation number of first-row transition metal(II) ions in solvent mixtures of N,N-dimethylformamide and N,N-dimethylacetamide—Solvation steric effect. *PCCP* **3**, 5475-5481 (2001).
5. Wu H, Ang JM, Kong J, Zhao C, Du Y, Lu X. One-pot synthesis of polydopamine–Zn complex antifouling coatings on membranes for ultrafiltration under harsh conditions. *RSC Adv.* **6**, 103390-103398 (2016).
6. Brown AJ*, et al.* Interfacial microfluidic processing of metal-organic framework hollow fiber membranes. *Science* **345**, 72 (2014).
7. Cheng Z, Chen C, Huang J, Chen T, Liu Y, Liu X. Nondestructive grafting of PEI on aramid fiber surface through the coordination of Fe (III) to enhance composite interfacial properties. *Appl. Surf. Sci.* **401**, 323-332 (2017).
8. Cheng Z, Yin Q, Wu H, He T, Luo L, Liu X. Regulating Cu(II)-benzimidazole coordination structure in rigid-rod aramid fiber and its composites enhancement effects. *Compos. Sci. Technol.* **184**, 107837 (2019).
9. Gold DH, Gregor HP. METAL—POLYELECTROLYTE COMPLEXES. VII. THE POLY-N-VINYLIMIDAZOLE SILVER(I) COMPLEX AND THE IMIDAZOLE—SILVER(I) COMPLEX. *The Journal of Physical Chemistry* **64**, 1461-1463 (1960).
10. Pearson RG. Hard and Soft Acids and Bases. *J. Am. Chem. Soc.* **85**, 3533-3539 (1963).
11. Villalobos LF, Karunakaran M, Peinemann K-V. Complexation-Induced Phase Separation: Preparation of Composite Membranes with a Nanometer-Thin Dense Skin Loaded with Metal Ions. *Nano Lett.* **15**, 3166-3171 (2015).
12. Lu W, Yuan Z, Zhao Y, Zhang H, Zhang H, Li X. Porous membranes in secondary battery technologies. *Chem. Soc. Rev.* **46**, 2199-2236 (2017).
13. Rathod D*, et al.* Design of an "all solid-state" supercapacitor based on phosphoric acid doped polybenzimidazole (PBI) electrolyte. *J. Appl. Electrochem.* **39**, 1097-1103 (2009).
14. Zhu S, Yan L, Zhang D, Feng Q. Molecular dynamics simulation of microscopic structure and hydrogen bond network of the pristine and phosphoric acid doped polybenzimidazole. *Polymer* **52**, 881-892 (2011).
15. Haisch T, Ji H, Weidlich C. Monitoring the state of charge of all-vanadium redox flow batteries to identify crossover of electrolyte. *Electrochim. Acta* **336**, 135573 (2020).
16. Yeh H-C, Wang M, Chang C-C, Yang R-J. Fundamentals and Modeling of Electrokinetic Transport in Nanochannels. *Isr. J. Chem.* **54**, 1533-1555 (2014).
17. Chen D, Kang C, Duan W, Yuan Z, Li X. A non-ionic membrane with high performance for alkaline zinc-iron flow battery. *J. Membr. Sci.* **618**, 118585 (2021).
