# Supplementary material for: Metal-coordinated polybenzimidazole membranes with preferential K+ transport
Source: Nat Commun. 2023 Mar 1;14:1149. doi: 10.1038/s41467-023-36711-w (PMC9975182; doi:10.1038/s41467-023-36711-w)
Supplement: Supplementary file 4 — Description of Additional Supplementary Files [file 41467_2023_36711_MOESM4_ESM.docx]

**Description of Additional Supplementary Files**

Supplementary Movie 1

Description: The forming of surface Turing patterns for Zn-PBI.
