# Supplementary material for: Metal-coordinated polybenzimidazole membranes with preferential K+ transport
Source: Nat Commun. 2023 Mar 1;14:1149. doi: 10.1038/s41467-023-36711-w (PMC9975182; doi:10.1038/s41467-023-36711-w)
Supplement: Supplementary file 2 — Peer Review File [file 41467_2023_36711_MOESM2_ESM.pdf]

nature portfolio

Peer Review File

Metal-coordinated polybenzimidazole membranes with preferential K+ transport

Open Access This file is licensed under a Creative Commons Attribution 4.0 International License, which permits use, sharing, adaptation, distribution and reproduction in any medium or format, as long as you give appropriate credit to the original author(s) and the source, provide a link to the Creative Commons license, and indicate if changes were made. In the cases where the authors are anonymous, such as is the case for the reports of anonymous peer reviewers, author attribution should be to 'Anonymous Referee' followed by a clear attribution to the source work. The images or other third party material in this file are included in the article's Creative Commons license, unless indicated otherwise in a credit line to the material. If material is not included in the article's Creative Commons license and your intended use is not permitted by statutory regulation or exceeds the permitted use, you will need to obtain permission directly from the copyright holder. To view a copy of this license, visit http://creativecommons.org/licenses/by/4.0/.

REVIEWER COMMENTS

Reviewer #1 (Remarks to the Author):

This manuscript presents the fabrication of highly K+ selective PBI membranes by accumulating PBI chains using metal ions and demonstrates its high performance in an alkaline zinc-iron flow battery. The authors demonstrated that the Zn-PBI membrane effectively controls K+ transport and blocks active species crossover, thus enhancing coulombic efficiency (CE) and voltage efficiency (VE). They also tried to explain the membrane porous structure and ion transport mechanism using molecular dynamics simulation and spectroscopy studies. However, to publish this manuscript, the following points should be seriously considered by the authors.

1) The authors only showed the conceptual illustration of the tunable ion-transport channels in Fig. 1, Fig. 2, and Fig. 5. Although the authors provided data for the metal ion-PBI interaction and suggested the ion channel structure of the membranes, the experimental data and analysis for the ion channel dimension are critical to explaining ion transport and selective mechanism of the membrane. In particular, the monovalent ion exclusion test indicates that the membrane follows not only the Donnan exclusion mechanism but also the size exclusion mechanism. The authors should provide comprehensive experimental data such as SAXS, PALS, N2 adsorption for pore size analysis, and HRTEM.
2) The authors stated several times about polar groups or charges inside channels but failed to provide any data. What are the Zn+ and imidazole unit density on the ion channel surface and the surface charge density of the channel? I also suggest that the authors should provide the zeta potential value of the membrane as a function of pH.
3) To better explain the effect of the electric double layer, the authors should address ion concentration, ionic strength, and Debye length.
4) I wonder why the authors tested the cell performance only at 80 mA/cm2. To explain better the correlation between CE, VE, and EE, I recommend that the authors provide efficiencies of the flow battery cell at several different current densities (e.g., 40 mA/cm2 – 150 mA/cm2).
5) I would recommend that the authors compare the battery performance of the Zn-PBI membrane to reference membranes (e.g., commercial cation exchange membrane, PBI membrane).
6) The authors also should provide charge/discharge curves of the battery cell.

Minor: the authors should carefully check the figure captions and figure numbers in the main text. For example,
1) Fig 2. (d) -> (c)

Reviewer #2 (Remarks to the Author):

The manuscript reports on membranes with controllable K+ transport developed via tunable chain accumulation induced by metal ions that coordinate with the polymer chains at various rates to reshape the original randomly-accumulated segments. In particular, the reaction of metal ions with polybenzimidazole at slower rate allows improved packing of the chains, expanding the inner accessible surface and exposing more polar groups, leading to ultrahigh K+ transport number.

The high K+-transport performance suppress water transmembrane migration in an alkaline zinc-iron flow battery, improving its lifespan and stably.

The work is based on the concept of regulating ions transport behavior by tunning polymer chains accumulation induced by metal ions coordination, which is not novel but effectively demonstrated in the present case.

The experimental part is well described, allowing reproducibility of the results and theoretical calculations are a suitable and needed complement well integrated in to the discussion of the results. Further, the battery application confirms the suitability of the approach.

On the other hand:

a) The state of the art basically is missing with respect to the alternative strategies to develop controllable ion transport channels. This is important to further properly discuss the advantages and drawbacks with respect to existing strategies;
b) The membrane forming strategy based on coordination-induced phase transformation depends on a variety of parameters that have been not explained properly. Please, discuss deeply the processing-morphological characteristics of the membranes, in particular the solvent-nonsolvent exchange, that is not enough supported by the data;
c) The paper has no error bars or uncertainties, even when many results seem to be within experimental error;
d) The interpretation of the variations in the thermal degradation are speculative. Please support properly;
e) Finally, the high K+-transport performance in Zn-PBI is convincingly demonstrated but how it balance ionic strength during charging and suppress water transmembrane migration is not clear. Please, discuss in a more convincing and less speculative way.

After those issues are considered properly, the work can be evaluated for publication.

Reviewer #3 (Remarks to the Author):

This manuscript reports the metal ion coordinated polybenzimidazole membranes and their application as ion-selective membranes in flow batteries. The authors performed extensive materials characterization and computational simulation to study the polymer chain packing and structural transformation as a result of metal ion coordination. The metal ion coordination changed the packing of polymer chains and distribution of free volume elements, therefore leading to membranes with ion channels of different shapes and geometries. The resulting membranes show selective ion transport, especially high K+ transport number. The membranes were integrated in alkaline Zn-Fe flow batteries. The strategy of using metal ion coordination to tune the polymer structure and ion transport is innovative. The results are certainly important and suitable for publication in a high-profile multidisciplinary journal like Nature Communications, but the authors should address the following major concerns.

The major problem is the poor logic flow of presentation of results. There are also concerns about computational simulation and data presentation (see detailed comments below). The authors performed modelling of the metal ion-polymer interactions, as well as ion transport in the metal-ion coordinated polymer network, the explanation of results is difficult to follow. Another major problem of this manuscript is the poor English language throughout the paper and supporting information. There are grammar mistakes or poor use of words in almost every sentence, making it really difficult to read. In my opinion, the manuscript needs to be rewritten. Proper proof-reading and carefully check of language are required.
Major comments:
1. Polybenzimidazole is known to have dense packing due to the strong hydrogen bonding between imidazole groups of linear chains, leading to low intrinsic ionic conductivity. Metal ion coordination of polybenzimidazole has been reported recently, including the authors’ own work published in JACS (I was one reviewer of that paper). These previous studies generally suggest that the metal ion coordination of polybenzimidazole introduces crosslinking between polymer chains, increases the rigidity of the polymer network, and possibly increased the free volume elements. Similarly, Cu2+-coordination of densely packed polymers such as cellulose has been demonstrated to expand the spacing between polymer chains, forming nanosized ionic channels (Nature, 2021). These previous studies should be cited and critically evaluated in the introduction, and the novelty of this work should be justified.
References
Liu, T., Huang, H., Wang, Y., Yu, J. and Hu, Z. (2022), Super Strong and Tough Polybenzimidazole/Metal Ions Coordination Networks: Reinforcing Mechanism, Recyclability, and Anti-Counterfeiting Applications. Macromol. Rapid Commun., 43: 2100643.

https://doi.org/10.1002/marc.202100643
J. Mater. Chem. A, 2021,9, 14400-14410; https://doi.org/10.1039/D1TA02601A
J Am Chem Soc, 2021, 143 (33), 13135-13144.

Yang, C., Wu, Q., Xie, W. et al. Copper-coordinated cellulose ion conductors for solid-state batteries. Nature 598, 590–596 (2021). https://doi.org/10.1038/s41586-021-03885-6

2. The explanation of metal ion coordination and formation of K ion channels within the metal-ion coordinated polymer network are difficult to follow. The authors are recommended to re-organize the figures and results so the logic flow can be improved. At the moment, diagrams and simulation results are mixed but they do not match each other well, and the simulation figures are usually of poor quality, so it is not clear how the metal ions and chains interactions change the packing of polymer chains. I found that the ion channel diagrams shown in Fig. 1 are not so convincing because the authors do not have direct evidence to prove these hypotheses. The simulation data of polymer network with metal ions are not very clear, so it is difficult to tell the difference on a molecular level. The diagram shown in Fig. 1b is also misleading, it suggests the large interchain spacing between two independent PBI chains. In reality, the metal-ion coordinated polymer network is disordered and the K^+ ions might transport through hoping through the disordered channels.
As zinc ion coordination is more effective compared to Cr and Fe ions, I suggest the authors to focus on presenting the results and mechanism on Zinc-ion coordination. For example, the metal ion coordination mechanism can be presented in one figure, Fig. 3 can be combined with Figure 2 and 1.
The ion transport number in Fig.1 can be combined with the ion transport data shown in Fig. 4 and Fig. 5.
3. The molecular dynamics simulation on the ion transport is flawed. As shown in Figure 4, only K^+ and Cl^- are shown in the polymer network. What about water molecules?
4. The authors considered the reaction rates of metal ion coordination as a critical parameter but quantitative analysis of the reaction rate was not provided. Are you able to provide some quantitative data, such as reaction constants?
5. The ion transport number measured in KCl and KOH electrolytes should be carefully checked. The authors attributed the high K^+ selectivity to interaction with polar groups, e.g. oxygen in PBI. What about PBI membrane without the ether-linkages, such as m-PBI?
6. It is known that high concentration KOH swells PBI membranes and the ion conductivity can be significantly enhanced. In swollen membranes, the transport of OH^- ions may become dominant. Reference (Energy Environ. Sci., 2019, 12, 3313-3318).
7. The unit of resistance is wrong. It should be Ω cm^2.
8. The permeation data shown in Fig. 6a is confusing. The permeation should follow Fick’s law and there should be a slope of concentration increase from zero. But the $Fe(CN)_6^{4-}$ concentration are constant for PBI-D and Zn-PBI.
9. The membranes are demonstrated in an alkaline Zinc-$Fe(CN)_6$ battery. Such battery system uses alkaline KOH electrolyte as a supporting electrolyte. According to the authors’ previous work, OH^- conductive membranes also work well in such battery system. It would be good to explore if the K^+-selective membrane works in a battery system that relies on K-ion transport, such as an anthraquinone-based organic redox flow battery operated in neutral pH electrolytes (e.g. KCl).
10. The English language needs to be improved significantly.

Some examples in the abstract:
“Polymer chain accumulation”, this is very difficult to understand from polymer science perspective. I assume the authors mean interchain association (or aggregation) due to metal ion coordination. Aggregation might be not very accurate. It should be noted that PBI polymer chains are usually considered densely packed due to strong hydrogen bonding between the imidazole functional groups. To some extent, the polymer chains are originally densely aggregated. The metal ion coordination works like crosslinking and changes the packing of polymer chains.
“inherent random kink of polymer segments”. Do you mean entanglement of polymer chains? Kink is not very accurate.
“triggering chains contorting to varying degrees”: do you mean chain rigidity?
“packed effectively and evenly”, this seems to disagree with your point. If you mean the PBI chains pack efficiently, then you would not get chain rigidity. I assume that you mean the polymer

chain coordination is more uniform for Zn-PBI.
"expand the inner accessible surface": the accessible surface was not quantitively defined. Could you provide some quantitative analysis of the surface area probed by small molecules?
"superiority of K+ transport", do you mean preferential K+ transport?
"Unwind solvent (N, N-Dimethylformamide)"– do you mean desolvation?
"Notably the slow is relatively speaking."
"Slow the RW, the segments packed more effectively and evenly." , many sentences like this one should be rewritten.
11. References are poorly cited and formatted. The authors should cite references that are more relevant to the work, especially metal-ion coordinated polymers. References 1-4 are not directly relevant (reference 2 and 4 are related to organic solvent nanofiltration, instead of ion transport). Similarly, many other papers cited in the references are not so relevant. Note that some references are repeated. Reference 26 and 32 are the same.
12. The title can be changed to: Metal-ion coordinated polybenzimidazole membranes with preferential K+ transport for alkaline zinc-iron flow battery

More detailed comments on simulation:
13. The authors have reported a large set of very computationally expensive simulations, but they have not taken full advantage of these by extracting meaningful and quantitative results from them. Most of the simulation data reported is either images or comments which should be supported by detailed numerical data. For example:

a. Extracting the ion residence times or mean squared displacements across the MD trajectory would much better support their arguments towards ion transport. Figure 4f is not useful to fully describe the K+ transport.
b. It might also be interesting to see comparisons of the density distributions (Figure 4b) at different time intervals during the simulation.
c. The authors make a lot of statements about the membrane structure in the simulations such as "increased inner water accessible surface" or "reduces the local aggregation of chains and produces numerous fine and dense channels". I cannot see evidence to support these comments on the internal pore architecture from the simulations.
14. The authors should carefully consider some interpretations of their simulation data. For example:
a. Figure 4c shows that the K+ ions are more strongly bound to oxygen in the Zn-PBI system, shown by a higher intensity of the first rdf peak. Why does this promote K+ transport? More strongly bound cations seems like it would lead to slower transport.
b. Figure 4d shows that K+ is more strongly bound to nitrogen atoms in Fe-PBI. Similarly, 4e shows that Cl- is more strongly bound to nitrogen atoms in Zn-PBI. This does not necessarily mean they are sterically blocked.

15. There are a range of simulations reported in the ESI, but it is very difficult to follow which simulation the authors are referring to in the main text. It is also unclear what the motivation is for producing so many different simulations set ups.

16. The computational methods might be hard to follow and reproduce. For example:

a. "CrCl3, FeCl3, ZnCl2, KCl, and counter ions were added according to the experimental condition." What were the counter ions and how many?
b. The authors describe that the double layer systems "went through a series of ion-water swapping and MD relaxing processes". There are no details of this protocol.
c. Table S6. Shows details of the different simulation set-ups. Could the authors elaborate why there were different production times for the different systems?
d. They should also report the number of water molecules in each system.

17. Overall, I would suggest rearranging the computational results and discussion to make it easier to follow. For example, a standalone computational results section might work better.

RESPONSE TO REVIEWERS' COMMENTS

Reviewer #1 (Remarks to the Author):

This manuscript presents the fabrication of highly K^+ selective PBI membranes by accumulating PBI chains using metal ions and demonstrates its high performance in an alkaline zinc-iron flow battery. The authors demonstrated that the Zn-PBI membrane effectively controls K^+ transport and blocks active species crossover, thus enhancing coulombic efficiency (CE) and voltage efficiency (VE). They also tried to explain the membrane porous structure and ion transport mechanism using molecular dynamics simulation and spectroscopy studies. However, to publish this manuscript, the following points should be seriously considered by the authors.

1) The authors only showed the conceptual illustration of the tunable ion-transport channels in Fig. 1, Fig. 2, and Fig. 5. Although the authors provided data for the metal ion-PBI interaction and suggested the ion channel structure of the membranes, the experimental data and analysis for the ion channel dimension are critical to explaining ion transport and selective mechanism of the membrane. In particular, the monovalent ion exclusion test indicates that the membrane follows not only the Donnan exclusion mechanism but also the size exclusion mechanism. The authors should provide comprehensive experimental data such as SAXS, PALS, N_2 adsorption for pore size analysis, and HRTEM.

Response: We appreciate the reviewer's constructive suggestion. Indeed, the ions channel dimension of the membrane is essential to understand the ion transport mechanism. To better calrify the porous structure of prepared membranes. The positron annihilation lifetime (PAL) spectroscopy was further carried out and provided to describe the pore size in M-PBI (Fig.2a in the revised version).

As shown in (a), the average free volume cavity radius (R) was calculated using the *o*-Ps lifetime τ_3, which indicates the average aperture of the M-PBI shows little difference. Relatively, the R of Zn-PBI is the smallest (0.332 nm) and cavity number density in the bulk is significantly higher than Cr-PBI and Fe-PBI (b). It implies the PBI chains can be packed effectively and evenly for Zn-PBI as discussed in the manuscript.

To clairfy this, more discussion was added in the revised version. (Line 1 to 5 on page 5)

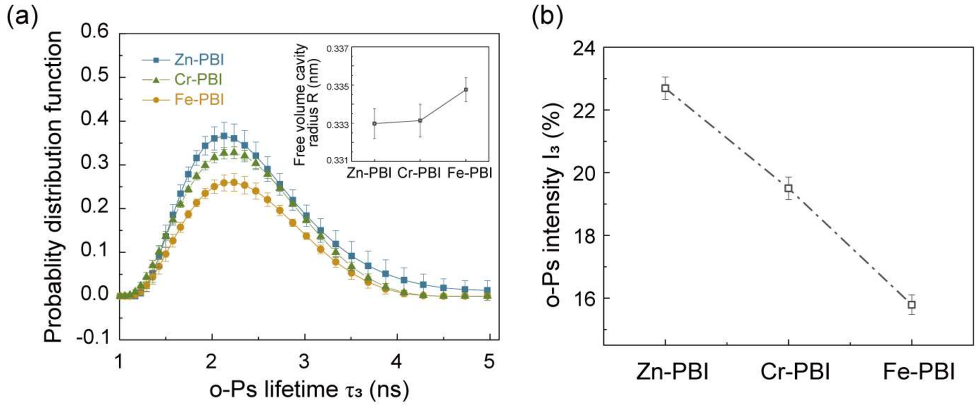

2) The authors stated several times about polar groups or charges inside channels but failed to provide any data. What are the Zn^{+} and imidazole unit density on the ion channel surface and the surface charge density of the channel? I also suggest that the authors should provide the zeta potential value of the membrane as a function of pH.

Response: We appreciate the reviewer's very constructive comments. The bulk chemical compositions of Zn-PBI were obtained from the ICP and ONH elemental analyzer (Supplementary Table 2). We quantitively evaluate the Zn^{2+} and imidazole unit, demonstrating that the approximate one Zn^{2+} attached to two PBI segment repetitive units (four imidazole units).
Indeed, the inner ion channel properties should be further studied. As shown in (a), the zeta potential value of Zn-PBI was characterized in 1mM KCl solution (PH varies from 3 to 10). As the increase of PH, Zn-PBI becomes more negatively charged, due to de-protonation of the imidazole groups at a higher PH.

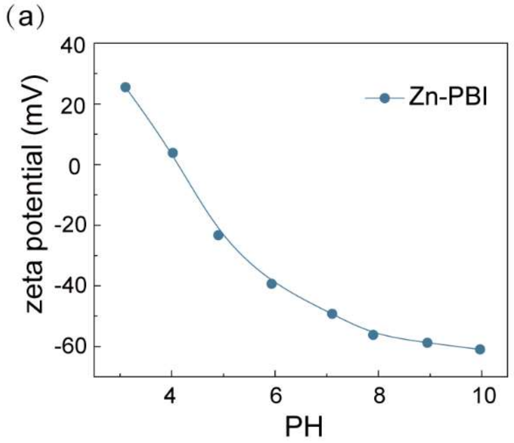

According to the Gouy-Chapman equation, the surface charge density (σ, mC m^{-2}) of the membrane surface was calculated and shown in (b) (doi.org/10.1016/S0376-7388(97)00278-0, doi.org/10.1016/j.isci.2021.102369)

$$|\sigma| = \varepsilon\kappa\xi\frac{sinh(\frac{F\xi}{2RT})}{\frac{F\xi}{2RT}}$$

where ε is permittivity (6.933×10^{-10} F m^{-1}), $\boldsymbol{\kappa}^{-1} = (\frac{\varepsilon RT}{2F^2c})^{1/2}$ is Debye length (nm), ξ is surface zeta potential (mV), F is Faraday constant (96485 C mol^{-1}), R is gas constant (8.314 J mol^{-1} K^{-1}), T is the absolute temperature (298 K), c is the bulk concentration.

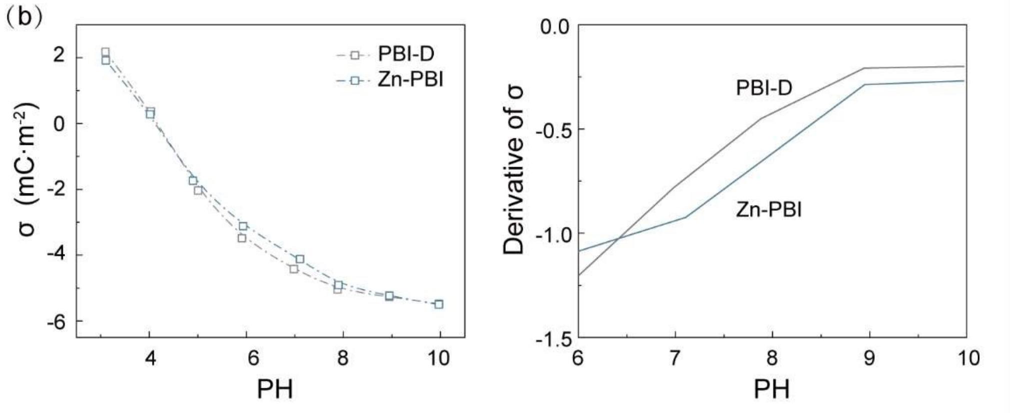

The surface charge density of Zn-PBI increases at a faster rate than PBI-D, because the absolute value of the first derivative of σ is larger. This trend implies that Zn^{2+} coordination intensifies the charge property (https://doi.org/10.1021/acs.nanolett.1c02919). That means the electrons of Zn^{2+} can delocalize to the whole PBI segments, raising the negative charge of the main chains, as we discussed in the manuscript.

To clarify this, more discussion was added in the revised version (Line 8 to 13 from the bottom on page 8).

3) To better explain the effect of the electric double layer, the authors should address ion concentration, ionic strength, and Debye length.

Response: Refer to the response to question (2), the Debye length can be obtained as follows:

$$\lambda_D = \left(\frac{\varepsilon RT}{2F^2 c}\right)^{1/2}$$

Where the concentration (c) is the bulk concentration. When the KCl solution increased from 0.33 M to 3 M, the Debye length (λ_D) decreases from 0.529 nm to 0.176 nm. To clarify this, more discussion was added in the revised version. (Line 9 to 11 on page 9).

4) I wonder why the authors tested the cell performance only at 80 mA/cm^2. To explain better the correlation between CE, VE, and EE, I recommend that the authors provide efficiencies of the flow battery cell at several different current densities (e.g., 40 mA/cm^2 – 150 mA/cm^2).

Response: Thanks for the reviewer's advice. To better compared the battery performance, the performance of AZIFB assembled with Zn-PBI at the different current densities (40, 80, 120, 160 mA cm^{-2}) was added in Supplementary Fig. 14.
When increasing the current efficiencies, the CE barely diminished, while the VE decreased. Because the ohmic polarization of the battery increases at high current densities.

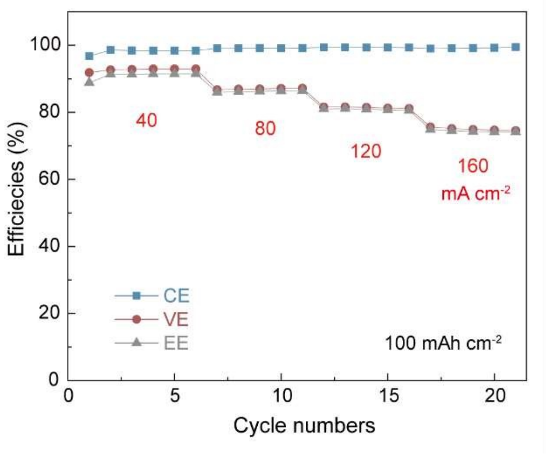

5) I would recommend that the authors compare the battery performance of the Zn-PBI membrane to reference membranes (e.g., commercial cation exchange membrane, PBI membrane).

Response: We appreciate the reviewer's advice. As suggested, the commercial cation exchange membrane (Nafion 212) was used as the reference. The charge/discharge curves of batteries when using Nafion 212 and Zn-PBI were given in Supplementary Fig. 15, which was attached below:

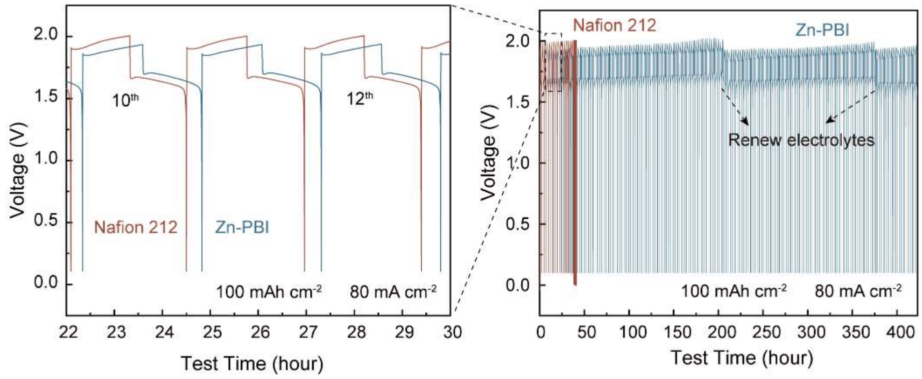

The AZIFB with Nafion 212 ran for less than 50 hours at the same operation condition, while the battery can stably run for more than 340 when using Zn-PBI. Referring to the previous report (doi.org/10.1016/j.memsci.2020.118585), it speculated that Nafion 212 was unable to withstand the zinc dendrite at a high areal capacity of 100 mAh cm^{-2} and a high current density of 80 mA cm^{-2}.

Enlarge charge/discharge curves of the battery, it can find that the polarization of the battery with Nafion 212 is larger than that of Zn-PBI, which leads to the gradual increase in battery polarization and eventually results in battery failure.

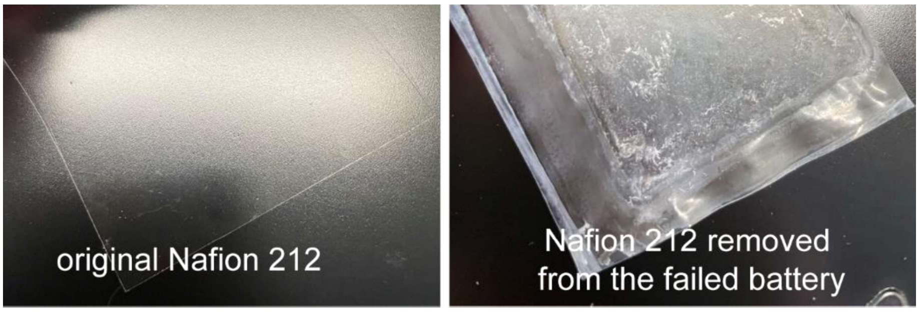

6) The authors also should provide charge/discharge curves of the battery cell.

Response: As suggested, we have added the charge/discharge curves of the battery.
The charge/discharge curves of the batteries using Zn-PBI at the 20th, 60th and 120th cycle were selected and magnified in Fig 5e, which was attached below:

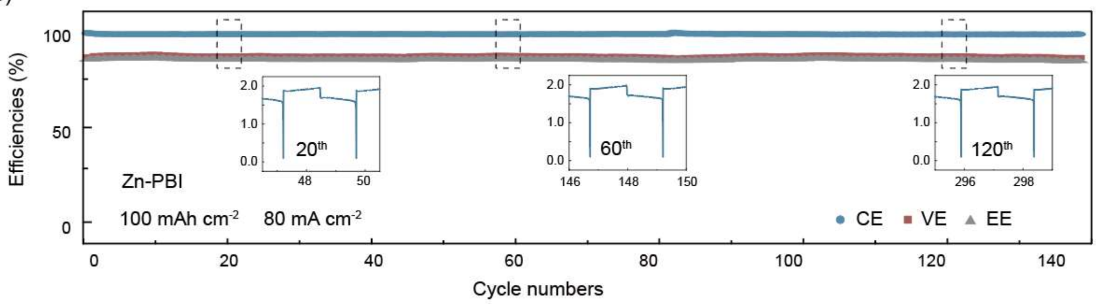

The complete charge/discharge curves were provided in Supplementary Fig. 17. In Supplementary Fig. 17, we further compared the charge/discharge curves of batteries when using the commercial cation exchange membrane (Nafion212) and Zn-PBI.

Minor: the authors should carefully check the figure captions and figure numbers in the main text. For example,
1) Fig 2. (d) -> (c)

Response: Sorry for the confusion. We carefully checked the figure caption and numbers and made the corresponding corrections.

Reviewer #2 (Remarks to the Author):

The manuscript reports on membranes with controllable K^+ transport developed via tunable chain accumulation induced by metal ions that coordinate with the polymer chains at various rates to reshape the original randomly-accumulated segments. In particular, the reaction of metal ions with polybenzimidazole at slower rate allows improved packing of the chains, expanding the inner accessible surface and exposing more polar groups, leading to ultrahigh K^+ transport number.

The high K^+-transport performance suppress water transmembrane migration in an alkaline zinc-iron flow battery, improving its lifespan and stably.

The work is based on the concept of regulating ions transport behavior by tunning polymer chains accumulation induced by metal ions coordination, which is not novel but effectively demonstrated in the present case.

The experimental part is well described, allowing reproducibility of the results and theoretical calculations are a suitable and needed complement well integrated in to the discussion of the results. Further, the battery application confirms the suitability of the approach.

Firstly, we appreciate the reviewer's positive comments on our paper.

On the other hand:

a) The state of the art basically is missing with respect to the alternative strategies to develop controllable ion transport channels. This is important to further properly discuss the advantages and drawbacks with respect to existing strategies;

Response: We appreciate the reviewer's very valuable comments. As suggested, more discussion about the alternative strategies for controllable ion transport channels has been supplemented in the Introduction. Relevant works were cited and objectively evaluated in the Introduction to justify the novelty of this work. We think the revised version help readers to know about existing strategies.

b) The membrane forming strategy based on coordination-induced phase transformation depends on a variety of parameters that have been not explained properly. Please, discuss deeply the processing-morphological characteristics of the membranes, in particular the solvent-nonsolvent exchange, that is not enough supported by the data;

Response: We appreciate the reviewer's advice. As the reviewer said, it is crucial to study the processing-morphological characteristics of the membranes. We have carried out a series of experiments to research the membrane-forming parameters. We tried our best to do it but unfortunately, it is really difficult to explain and cover all the issues systematically and comprehensively in the present work, as this is a complex process. In unpublished experiments, several parameters were proved to affect the membrane morphology, including the types and concentration of metal ions, the reaction time, the polymer concentration, and

the coating thickness. Since the mentioned parameters affected the overall coordination reaction.

In a certain reaction time, increase the concentration of metal ions and polymer, or reduce the coating thickness, the surface patterns space of membranes becomes narrower, as follows:

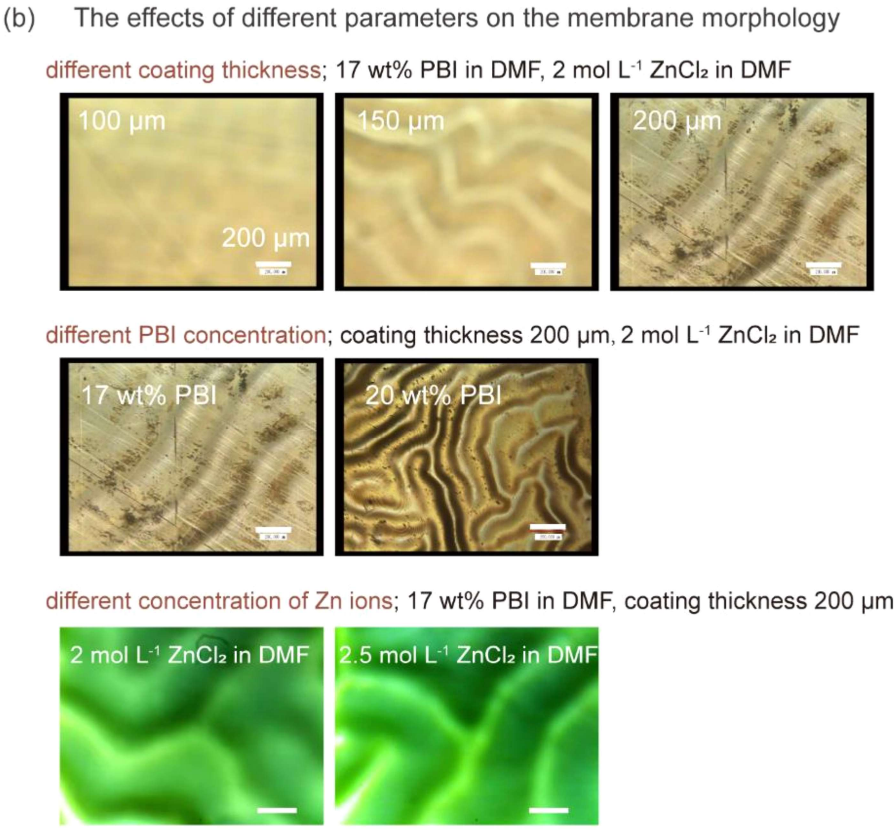

Our experiments demonstrated the influence of reaction time on membrane morphology. The below figure shows the development of cross-section morphology for Co-PBI when PBI gel immerses in 2 mol L^{-1} $CoCl_2$/DMF for 1min, 2min, 3min, and 4min. Co^{2+} with a slower reaction rate than Zn^{2+} was selected to better analyze the coordination process. As we can see, metal ions react with PBI from the top (the side near the MClx-DMF) to the bottom in the coordination-induced phase transformation and the unreacted PBI is transformed into macroporous which is the representative morphology formed in water. Within a sufficient reaction time, M-PBI is fully coordinated and will show complete dense cross-section morphology.

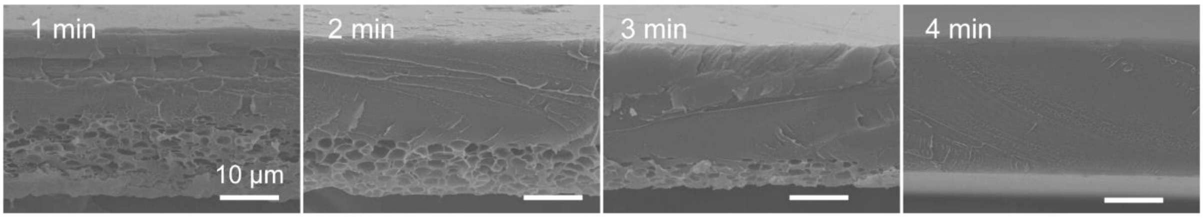

We tried to capture the intermediate state of membrane formation. However, in this work, the reaction between PBI and metal ions (Zn^{2+}, Fe^{3+}, Cr^{3+}) is too fast to do that. As mentioned above, in another of our ongoing experiments, we have found that Co^{2+} can achieve a slower reaction, which is expected to further deeply understand processing-morphological characteristics, especially the solvent-nonsolvent exchange. The research is still in progress and it's a pity that we can't present our work completely in the current work.

To better clarify it, we added the discussion about the morphology of membranes prepared with different concentrations of Zn ions, the polymer concentration, and coating thickness in the revised version (Line 16-18 on page 6) and revised SI (Supplementary Fig. 7).

c) The paper has no error bars or uncertainties, even when many results seem to be within experimental error;

Response: Sorry for the confusion. Actually, all the experiments were carried out in at least 2 parrel experiments. And as suggested, all point value data have been added with error bars in the revised version.

d) The interpretation of the variations in the thermal degradation are speculative. Please support properly;

Response: From the TGA curves, M-PBI exhibited better thermal stability than PBI-D. However, referring to the reference, it can only confirm the decomposition temperature of the PBI backbone (~520 °C). (DOI 10.1007/s10800-008-9764-3**)**

To avoid misunderstanding, we delete the relevant descriptions about the shedding of various metal ions in Supplementary Fig. 10.

e) Finally, the high K^+-transport performance in Zn-PBI is convincingly demonstrated but how it balance ionic strength during charging and suppress water transmembrane migration is not clear. Please, discuss in a more convincing and less speculative way.
Response: We appreciate the reviewer's constructive advice. We refer to relevant reports and tried to explain more deeply how the membrane affects the water transmembrane migration.

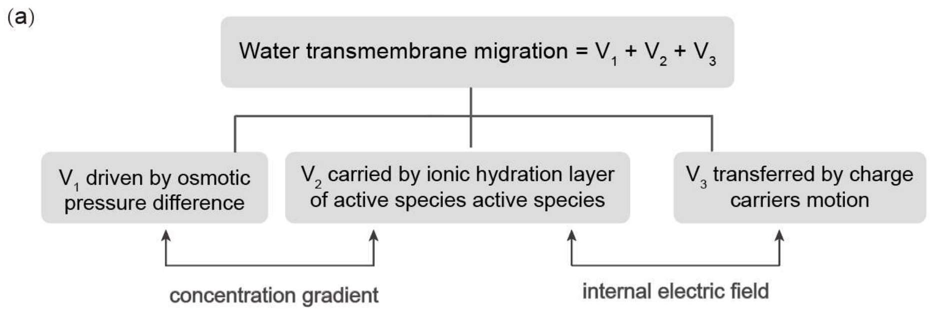

As shown in (a), the direction and amount of water migration are commonly jointly driven by three processes, including osmotic pressure difference in two half-cells (V_1), water carried by ionic hydration layer of active species (V_2) in tandem with water transferred by charge carriers motion (V_3). The fundamental driving force can be considered as concentration gradient and internal electric field. (References: https://doi.org/10.1016/S0378-7753(96)02463-9, https://doi.org/10.1016/S0376-7388(03)00309-0, https://doi.org/10.3390/membranes2020275**)**

(b)

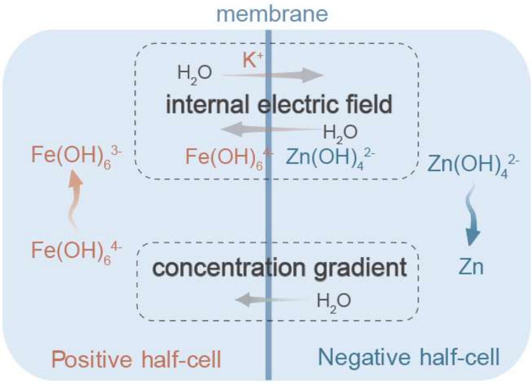

Charge Process in AZIFB

As for AZIFB, water generally moves towards the positive half-cell (PHC), increasing the volume of that side. As shown in (b), when an AZIFB is assembled with a cationic conductive membrane, during the charging process, charge carriers transport from the positive half-cell (PHC) to the negative half-cell (NHC) driven by the internal electric field. However, negative-charged active species in both PHC and NHC, that is $[Fe(CN)_6]^{4-}$and $Zn(OH)_4^{2-}$, carry water and migrate toward PHC driven by the electric field. Besides, $Zn(OH)_4^{2-}$ was reduced to Zn when charging, producing an imbalance of ionic strength (also concentration gradient), which further causes water to migrate. As a result, the volume of PHC increases.
In this regard, Zn-PBI is conducive to reducing the carried water in two ways. On the one hand, its tightly-stacked chains can effectively impede the crossover of negative-charge active species, on the other hand, during the battery operation, Zn-PBI is conducive to K^+ transport, which moves opposite to the way of active species. The water carried by the K^+ can partially counteract the water transferred by active species. That means Zn-PBI which encourages cations transport can thus reduce water migration volume more effectively. To clarify this, more discussion was added in the revised version. (Line 22 to 28 on page 11 in the revised version)

After those issues are considered properly, the work can be evaluated for publication.

Reviewer #3 (Remarks to the Author):

This manuscript reports the metal ion coordinated polybenzimidazole membranes and their application as ion-selective membranes in flow batteries. The authors performed extensive materials characterization and computational simulation to study the polymer chain packing and structural transformation as a result of metal ion coordination. The metal ion coordination changed the packing of polymer chains and distribution of free volume elements, therefore leading to membranes with ion channels of different shapes and geometries. The resulting membranes show selective ion transport, especially high K^+ transport number. The membranes were integrated in alkaline Zn-Fe flow batteries. The strategy of using metal ion coordination to tune the polymer structure and ion transport is innovative. The results are certainly important and suitable for publication in a high-profile multidisciplinary journal like Nature Communications, but the authors should address the following major concerns.

The major problem is the poor logic flow of presentation of results. There are also concerns about computational simulation and data presentation (see detailed comments below). The authors performed modelling of the metal ion-polymer interactions, as well as ion transport in the metal-ion coordinated polymer network, the explanation of results is difficult to follow. Another major problem of this manuscript is the poor English language throughout the paper and supporting information. There are grammar mistakes or poor use of words in almost every sentence, making it really difficult to read. In my opinion, the manuscript needs to be rewritten. Proper proof-reading and carefully check of language are required.

Firstly, we appreciate the reviewer's very positive comments on our paper. Sorry for the confusion on the grammer mistakes. As suggested, the manuscript has been carefully reorganized and checked including data and language, and hope this will help readers to follow. To meet the high standard of Nature Communications, our paper was further editored by Language Editing from Springer Nature Author Services.

Major comments:
1. Polybenzimidazole is known to have dense packing due to the strong hydrogen bonding between imidazole groups of linear chains, leading to low intrinsic ionic conductivity. Metal ion coordination of polybenzimidazole has been reported recently, including the authors' own work published in JACS (I was one reviewer of that paper). These previous studies generally suggest that the metal ion coordination of polybenzimidazole introduces crosslinking between polymer chains, increases the rigidity of the polymer network, and possibly increased the free volume elements. Similarly, Cu^{2+}-coordination of densely packed polymers such as cellulose has been demonstrated to expand the spacing between polymer chains, forming nanosized ionic channels (Nature, 2021). These previous studies should be cited and critically evaluated in the introduction, and the novelty of this work should be justified.

References
Liu, T., Huang, H., Wang, Y., Yu, J. and Hu, Z. (2022), Super Strong and Tough Polybenzimidazole/Metal Ions Coordination Networks: Reinforcing Mechanism, Recyclability, and Anti-Counterfeiting Applications. Macromol. Rapid Commun., 43:

2100643. https://doi.org/10.1002/marc.202100643
J. Mater. Chem. A, 2021,9, 14400-14410; https://doi.org/10.1039/D1TA02601A
J Am Chem Soc, 2021, 143 (33), 13135-13144.

Yang, C., Wu, Q., Xie, W. et al. Copper-coordinated cellulose ion conductors for solid-state batteries. Nature 598, 590–596 (2021). https://doi.org/10.1038/s41586-021-03885-6

Response: Thanks for the reviewer's advice. As suggested, we cited these references and supplemented the relevant discussion in the Introduction to confirm the novelty of our paper. The novelty of our paper is to show the structure and ion transport changes of PBI after coordination with metal ions.

2. The explanation of metal ion coordination and formation of K ion channels within the metal-ion coordinated polymer network are difficult to follow. The authors are recommended to re-organize the figures and results so the logic flow can be improved. At the moment, diagrams and simulation results are mixed but they do not match each other well, and the simulation figures are usually of poor quality, so it is not clear how the metal ions and chains interactions change the packing of polymer chains. I found that the ion channel diagrams shown in Fig. 1 are not so convincing because the authors do not have direct evidence to prove these hypotheses. The simulation data of polymer network with metal ions are not very clear, so it is difficult to tell the difference on a molecular level. The diagram shown in Fig. 1b is also misleading, it suggests the large interchain spacing between two independent PBI chains. In reality, the metal-ion coordinated polymer network is disordered and the K^+ ions might transport through hoping through the disordered channels.
As zinc ion coordination is more effective compared to Cr and Fe ions, I suggest the authors to focus on presenting the results and mechanism on Zinc-ion coordination. For example, the metal ion coordination mechanism can be presented in one figure, Fig. 3 can be combined with Figure 2 and 1. The ion transport number in Fig.1 can be combined with the ion transport data shown in Fig. 4 and Fig. 5.
Response: We appreciate the reviewer's very constructive comments. Actually, PBI is indeed a disordered chain segment. To better illustrate our idea, the Fig. 1b was reorganized to illustrate the transport mechanism. Considering the opinions of other reviewers, we retained the simplified structure in Fig. 1a to illustrate the different chains stacking of M-PBI, which is the focus of this paper.

3. The molecular dynamics simulation on the ion transport is flawed. As shown in Figure 4, only K^+ and Cl^- are shown in the polymer network. What about water molecules?

Response: Sorry for the confusion. Actually, the simulation was carried out in an aqueous environment. Following the suggestion, we studied the sectional and top views of water accessible surface in Zn^{2+}, Cr^{3+}, and Fe^{3+} restrained PBI systems. The sectional view in Supplementary Fig. 25 shows that inside the Zn-PBI, the uniform Zn^{2+} coordination results in more dispersed water among polymer chains, which may form constinous water channels for ion diffusion. While the Fe^{3+} cause different degrees of local constriction between imidazole units, which displays uneven water distribution in different region of polymer chains and makes chains over-aggregate to form larger channels.
More detailed descriptions were added on page 7 of the revised manuscript. Supplementary Fig. 25 is shown below:

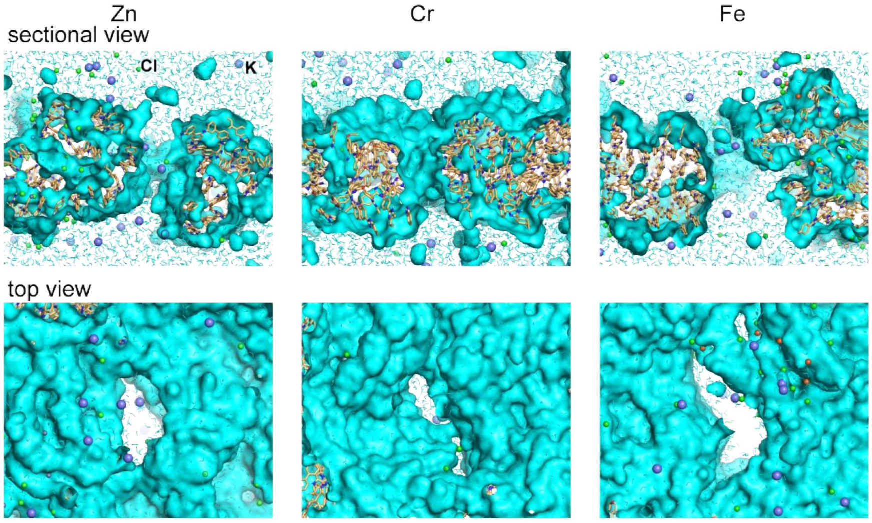

4. The authors considered the reaction rates of metal ion coordination as a critical parameter but quantitative analysis of the reaction rate was not provided. Are you able to provide some quantitative data, such as reaction constants?

Response: Thanks for the reviewer's advice. As the reviewer said, the reaction rate is a critical parameter to evaluate the reaction. However, it is difficult to obtain reasonable reaction constants by current experimental methods.

To calculate the rate constant of the reaction, more parameters are needed, including the consumed concentration of the reactants and products over a certain amount of time.

It is quite difficult to accurately count the above parameters through experiment because the M-PBI formed within 3s as shown in Supplementary Movie 1, which is relatively fast for experimental observation. As for current simulation methods, the time scale is not very suitable for our reaction. The current DFT simulation gives a trend of the reaction energy, but not the reaction rate. While the timescales for molecular simulation are too short, usually at the nanometer-scale. There is no way to reasonably give a period to get the product consumption.

5. The ion transport number measured in KCl and KOH electrolytes should be carefully checked. The authors attributed the high K^+ selectivity to interaction with polar groups, e.g. oxygen in PBI. What about PBI membrane without the ether-linkages, such as m-PBI?

Response: We appreciate the reviewer's advice. The PBI used in the experiment contains ether bonds. If it is PBI without an ether bond, the coordination situation between PBI and metal ions may be different, also affecting the pore structure of the metal-coordinated membrane, which may need further experimental proof.

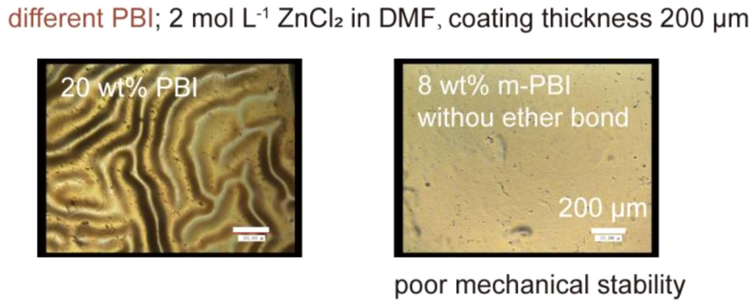

To better clarify it, we studied the coordination between Zn^{2+} and PBI without an ether bond (m-PBI). Due to the absence of ether bonds, the flexibility of m-PBI chains is reduced and its solubility in DMF is decreased. In our paper, the mass fraction of PBI with ether bond in DMF was prepared up to 20 wt%, but the m-PBI only reached 8 wt%.

When cross-linked with 2M $ZnCl_2$, PBI with ether bonds in the original paper formed a light yellow transparent membrane with surface patterns together with good mechanical stability. However, the membrane prepared by m-PBI is white opaque and showed a flat surface. A low mass fraction of m-PBI results in poor mechanical stability and is gently pulled to pieces, which can not be assembled with a device and tested.

6. It is known that high concentration KOH swells PBI membranes and the ion conductivity can be significantly enhanced. In swollen membranes, the transport of OH- ions may become dominant. Reference (Energy Environ. Sci., 2019, 12, 3313-3318).

Response: Thanks the reviewer for providing this idea. As the reviewer said, the PBI membrane is swollen in a high concentration of KOH, and the transport of OH^- may become dominant. Additionally, in a certain solution, the electrostatic interaction range (Debye length, λ_D) is defined. The swelling of the chain segment will make it difficult for Debye length to cover some passing ions, also weakening the Donnan effect.

The two reasons may lead the OH^- to be more competitive with K^+ through the membrane. While, in a high concentration KOH solution, the K^+ selectivity of Zn-PBI is still better than that of PBI-D, which verified the superiority of K^+ transport for Zn-PBI.

We have added this part of the discussion on page 9 of the revised manuscript and also cited the reference.

7. The unit of resistance is wrong. It should be Ω cm^2.

Response: Sorry for the mistakes. The unit of resistance has been revised as Ω cm^2.

8. The permeation data shown in Fig. 6a is confusing. The permeation should follow Fick's law and there should be a slope of concentration increase from zero. But the $Fe(CN)_6^{4-}$ concentration are constant for PBI-D and Zn-PBI.

Response: Sorry for the misunderstanding. We repeated the test and found that when the penetration time exceeded 2h, the absorbance on the penetration side of PBI-D would exceed

the detection limit. Thus at the first sampling point (24h) in the original Fig 6a, the absorbance of PBI-D permeation sample has exceeded the detection limit of the UV-vis spectrometer, resulting in inaccurate concentration calculation.

To avoid misunderstandings, the duration of the permeation experiment was shortened from 6 days to 1.25 hours. It can be seen from the following figure that Zn-PBI has better barrier performance for $[Fe(CN)_6]^{4-}$.

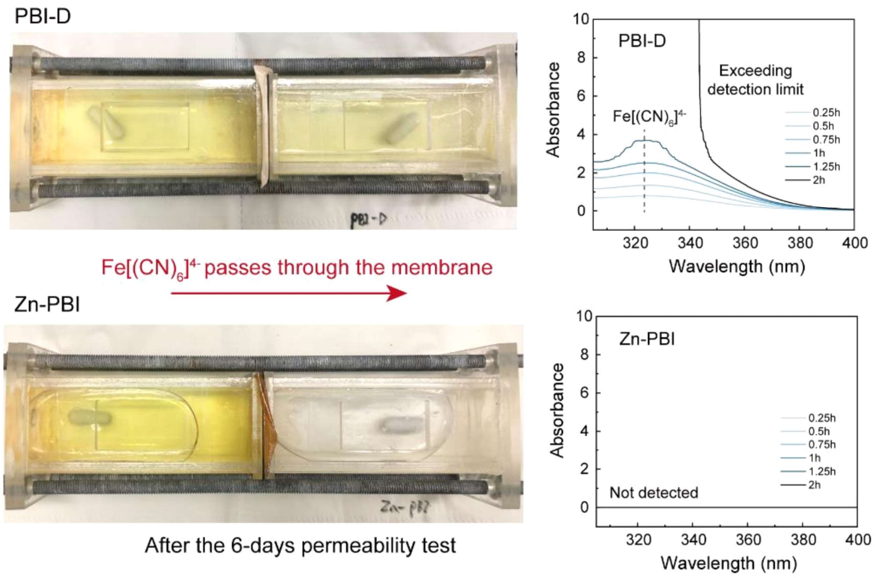

9. The membranes are demonstrated in an alkaline Zinc-Fe(CN)6 battery. Such battery system uses alkaline KOH electrolyte as a supporting electrolyte. According to the authors' previous work, OH^- conductive membranes also work well in such battery system. It would be good to explore if the K^+-selective membrane works in a battery system that relies on K-ion transport, such as an anthraquinone-based organic redox flow battery operated in neutral pH electrolytes (e.g. KCl).

Response: Thanks for the advice. We tried the neutral Zn-Fe flow battery before, but the electrolyte was not stable, so the performance of the membrane could not be well compared. We believe that the membrane properties can be better studied in a stable and mature battery system. Therefore, we finally chose the mature alkaline Zn-Fe flow battery as an illustration of membrane performance.

10. The English language needs to be improved significantly.
Some examples in the abstract:
"Polymer chain accumulation", this is very difficult to understand from polymer science perspective. I assume the authors mean interchain association (or aggregation) due to metal ion coordination. Aggregation might be not very accurate. It should be noted that PBI polymer chains are usually considered densely packed due to strong hydrogen bonding between the imidazole functional groups. To some extent, the polymer chains are originally densely aggregated. The metal ion coordination works like crosslinking and changes the packing of polymer chains.
"inherent random kink of polymer segments". Do you mean entanglement of polymer chains? Kink is not very accurate.

“triggering chains contorting to varying degrees”: do you mean chain rigidity?
“packed effectively and evenly”, this seems to disagree with your point. If you mean the PBI chains pack efficiently, then you would not get chain rigidity. I assume that you mean the polymer chain coordination is more uniform for Zn-PBI.
“expand the inner accessible surface”: the accessible surface was not quantitively defined. Could you provide some quantitative analysis of the surface area probed by small molecules?
“superiority of K+ transport”, do you mean preferential K+ transport?
“Unwind solvent (N, N-Dimethylformamide)”– do you mean desolvation?
“Notably the slow is relatively speaking.”
“Slow the RW, the segments packed more effectively and evenly.” , many sentences like this one should be rewritten.

Response: To improve the English language, the paper was polished by Language Editing from Springer Nature Author Services. Hopefully, the revised version can meet the publishing standard of Nature Communications.

11. References are poorly cited and formatted. The authors should cite references that are more relevant to the work, especially metal-ion coordinated polymers. References 1-4 are not directly relevant (reference 2 and 4 are related to organic solvent nanofiltration, instead of ion transport). Similarly, many other papers cited in the references are not so relevant. Note that some references are repeated. Reference 26 and 32 are the same.

Response: As the reviewer suggested, we checked and updated our references to make the references can reliably support the paper.

12. The title can be changed to: Metal-ion coordinated polybenzimidazole membranes with preferential K^+ transport for alkaline zinc-iron flow battery

Response: We appreciate the reviewer’s advice. The work is intended to show the structure and ion transport changes of PBI after coordination with metal ions, and the battery performance is used as a demonstration. So we would like to keep the title as it is.

More detailed comments on simulation:
13. The authors have reported a large set of very computationally expensive simulations, but they have not taken full advantage of these by extracting meaningful and quantitative results from them. Most of the simulation data reported is either images or comments which should be supported by detailed numerical data.

Response: We thank the reviewer for the helpful suggestions. Following the suggestion, we have made a few revisions to improve the clarity and accuracy of simulations on Page 7-8 of the revised manuscript and simulation section of Support Information (Supplementary Fig.16-S29).

For example:

a. Extracting the ion residence times or mean squared displacements across the MD trajectory would much better support their arguments towards ion transport. Figure 4f is not useful to fully describe the K^+ transport.

Response: We thank the reviewer for the helpful suggestion. Following the suggestion, we extracted the ion residence time as shown in Fig 3c of the revised manuscript and displayed the ion transport trajectories across the membrane in Supplementary Fig.27 of the revised SI. We also revise the presentation of the original Figure 3f for the description of Cl^- regulated K^+ transport as Fig 3e in the revised manuscript.

b. It might also be interesting to see comparisons of the density distributions (Figure 4b) at different time intervals during the simulation.

Response: We thank the reviewer for the helpful suggestion. Following the suggestion, we have displayed the density distributions at the beginning and after 40 ns as Supplementary Fig.24b of the revised SI for double-layer Zn^{2+}, Cr^{3+}, and Fe^{3+} restrained PBI systems. The density slightly decreased after 40 ns.

c. The authors make a lot of statements about the membrane structure in the simulations such as "increased inner water accessible surface" or "reduces the local aggregation of chains and produces numerous fine and dense channels". I cannot see evidence to support these comments on the internal pore architecture from the simulations.

Response: We thank the reviewer for the helpful suggestion. Following the suggestion, we have made a few modifications to improve the clarity and accuracy as follows,

For the "increased inner water accessible surface" statement, the original Fig S9a (as Supplementary Fig.23c in the revised SI) displays the hydrogen bonds formed between PBI (at N1, N2, and O) and water molecules to indicate that Zn-PBI system tends to expose more polar groups to solvent. In addition, we displayed sectional and top views of the water accessible surface in Zn^-, Cr^-, and Fe- PBI systems as the Supplementary Fig.25 in the revised SI. The sectional view shows that, inside the Zn-PBI, the uniform Zn^{2+} coordination results in more dispersion of water among polymer chains. While the Fe^{3+} cause different degrees of local constriction between imidazole units, which displays uneven water distribution in different region of polymer chains and makes chains over-aggregate to form larger channels.

For the "reduces the local aggregation of chains and produces numerous fine and dense channels" statement, we replace the original Fig 2d by snapshots of coordination domains from Zn-PBI and Fe-PBI simulations to illustrate that the Zn^{2+} coordination results in more uniform-distributed coordination domains in comparison with Fe^{3+} coordination domains, which reduces the local aggregation of chains and produces numerous fine and dense channels.

14. The authors should carefully consider some interpretations of their simulation data.

Response: We appreicate the reviewer's valualbe comemnts. To clarify this, we have made some modifications to better interpretate the simulation data. (See page 7 to 8 and Supplementary Fig.16-29).

For example:
a. Figure 4c shows that the K^+ ions are more strongly bound to oxygen in the Zn-PBI system, shown by a higher intensity of the first rdf peak. Why does this promote K^+ transport? More strongly bound cations seems like it would lead to slower transport.

Response: We thank the reviewer for the helpful suggestion. Following the suggestion, we have made a few modifications to improve the clarity for the role of O of chains in regulation of K^+ transport. As indicated in Supplementary Fig.29a (the original Fig 4c and d), the K^+ adsorption from O is much weaker than from –N= (N1), thus the K^+-O interactions are not strong enough to trap a K^+.

Modifications have been made on Page 8 of the revised manuscript as: MD simulation reveals that, although K^+ adsorption from O is much weaker than from –N= (N1), Zn-PBI attracts more K^+ around O than neither Fe- or Cr- PBI along the radial distribution (Supplementary Fig.29a). Also, the probability of K^+ interacting with O in Zn-PBI is greater than that in other membranes (Supplementary Fig.29b), which significantly helps to expedite K^+ transport.

To clarify this, more discussion was added in the revised version (See Line 8 to 15 on page 8).

b. Figure 4d shows that K^+ is more strongly bound to nitrogen atoms in Fe-PBI. Similarly, 4e shows that Cl- is more strongly bound to nitrogen atoms in Zn-PBI. This does not necessarily mean they are sterically blocked.

Response: We thank the reviewer for the helpful suggestion.

First, the K^+-N1 interaction plays as a control step for K^+ transport as indicated by the original Fig 4d (or Supplementary Fig.24b and 29a in the revised SI), thus stronger K^+ -N1 interactions in Fe-PBI (the original Figure 4d) tend to trap K^+ and lead to longer K^+ residence time in Fe-PBI (Fig 4c in the revised manuscript).

Second, the Cl^--N2 interactions plays as a control step for Cl^- transport as indicated by the original Fig 4e (or Supplementary Fig.29c in the revised SI), thus stronger Cl^--N2 interactions in Zn-PBI (the original Figure 4e) tend to trap Cl^- and lead to longer Cl^- residence time in Zn-PBI (Fig 3c in the revised manuscript). The trapped Cl^- further plays positive role in promoting the transport of K^+ as demonstrated in Fig 3e in the revised manuscript. Thus, the different preference for K^+ and Cl^- in Zn- and Fe- PBI lead to different ion residence time and high K^+ selectivity in Zn-PBI.

To clarify this, we have made the corresponding modifications on Page 7-8 of the revised manuscript.

15. There are a range of simulations reported in the ESI, but it is very difficult to follow which simulation the authors are referring to in the main text. It is also unclear what the motivation is for producing so many different simulations set ups.

Response: We thank the reviewer for the helpful suggestion. Following the suggestion, we have provided detailed descriptions regarding the motivation and major findings (particularly structure and interactions features) for different simulations in Supplementary Table 5 in the simulation section of the revised SI.

16. The computational methods might be hard to follow and reproduce. For example:

Response: We thank the reviewer for the helpful suggestion. To clarify this, we have provided more details regarding the computational methods in the simulation section of the revised SI.

a. "CrCl3, FeCl3, ZnCl2, KCl, and counter ions were added according to the experimental condition." What were the counter ions and how many?

Response: We thank the reviewer for the helpful suggestion. Following the suggestion, we have modified the description regarding counter ions in the simulation section of revised SI as follows:

For systems #2-4, 0.1 M $ZnCl_2$, $CrCl_3$, or $FeCl_3$ were added to the system. The lower concentration (0.02~0.03 M) of $CrCl_3$ was used in later systems like #6 and #10, since nucleation tendency was observed in 0.1 M $CrCl_3$ (Supplementary Fig.17). Addition of 0.15 M KCl were added to explore ion conduction in double-layer systems #13-15.

b. The authors describe that the double layer systems "went through a series of ion-water swapping and MD relaxing processes". There are no details of this protocol.

Response: As suggested, we have modified the description regarding the ions-water swapping protocol in the simulation section of revised SI as follows,

The double-layer systems were relaxed in ~100 ns MD simulation to reach equilibrated states, then went through a series of ion-water swapping and MD relaxing processes to study the ion transport in Zn-, Cr-, and Fe- PBI systems. In the ion-water swapping protocol, we exchange the coordinates of K^+ or Cl^- in one aqueous compartment with water molecules in the other compartment. We defined the exchange regions as the water regions 5~10 Å away from the PBI membrane. The swapping of ions and water generates ion concentration difference across the membrane. After the swapping, we relaxed the double-layer system in 80-ns MD simulation, during which we record the K^+ or Cl^- trajectories across the membrane. We simulated the ion-water swapping and MD relaxing process 20 times.

c. Table S6. Shows details of the different simulation set-ups. Could the authors elaborate why there were different production times for the different systems?

Response: We thank the reviewer for the helpful suggestion.

We first carried out a series of microsecond molecular dynamic (MD) simulations to study the structures of the PBI under different set-ups and PBI-metal ion interactions on molecular level. Since the structures and PBI-metal ion interactions reached relatively stable states (did not change much) after microsecond MD simulations, the simulations time for #1-9 and #16-18 were not restricted to exact the same simulation time. However, same production time are used for metal-restrained systems for data analysis. In addition, we increased the ion-water swapping and MD relaxing process to 20 times for all double-layer systems in Supplementary Table 5 in the revised SI.

To clarify this, more details were added in the revised SI. (See Supplementary Discussion on page 8 of SI).

d. They should also report the number of water molecules in each system.

Response: As suggested, we have provided the number of water molecules in each system in Supplementary Table 5 in the revised SI.

17. Overall, I would suggest rearranging the computational results and discussion to make it easier to follow. For example, a standalone computational results section might work better.

Response: As suggested, we have reorganized the computational results and hope to help readers to follow.

REVIEWER COMMENTS

Reviewer #1 (Remarks to the Author):

The authors have responded to all comments reasonably and revised the manuscripts by addressing all my comments. The manuscript has an improved quality, so I recommend it for publication after addressing the following minor comment.

Minor:
The difference in the surface charge calculation of PBI-D and Zn-PBI is small, which could be in the error range and could affect the derivative of the surface charge density. I recommend that the authors should include an error bar and revise the result and explanation.

Reviewer #2 (Remarks to the Author):

The authors have properly taken into consideration all comments from the referees and have addressed all relevant issues.

This referee supports publication of the work in the present form.

Reviewer #3 (Remarks to the Author):

I have reviewed this revised manuscript and their responses to comments given by all reviewers. The authors certainly have made efforts to address some of the concerns raised by all reviewers. The quality of the manuscript is improved, however, there are still many problems.
It is quite important to provide more experimental data to study the ion channel structures. As suggested by Reviewer 1, SAXS/WAXS, HRTEM, and gas sorption might be useful. The authors performed additional experiments including PALS to study the pore size or free volume elements. However, the PALS results are insufficient. WAXS might be useful to study the variation of inter/intrachain spacings.
As requested by reviewer 1, the authors updated typical charge/discharge curves as insets in the cycling data. But this is very difficult to read. It would be better to plot the charge/discharge data as voltage versus capacity and compare them in one plot.
Reviewer 2 raised one important question on water migration. The authors' response and discussion on ion transport and water migration seem to be reasonable, however, is it possible to provide any experimental evidence to support the claims? More quantitative analysis of the water diffusion coefficient through the membranes would also be valuable. Similarly in the discussion on Page 11, more quantitative data should be provided, for example, what were the crossover rates of Zn and iron species? Note that there are mistakes in the diagram, Fe(CN)6 instead of Fe(OH)6.
Reviewer 2 also made a good comment on the detailed parameters that influence the structural transformation. The authors did provide some great preliminary results to show the effect of metal ion concentration, reaction time, and polymer concentration. It is good to see that the authors included some of these results in the supporting information.
The authors updated the introduction to recent work on metal ion coordinated polymers including the Cu-Cellulose and Cu-PBI polymers (JACS). However, the novelty of this work over the previous work still needs to be emphasized. In my opinion, the novelty of this work lies in more in-depth understanding of the polymer chain packing by molecular modelling and characterization of the membranes coordinated by different metal ions as well as ion transport, which was not carefully studied in their previous JACs work.
The references cited by the authors are still poorly organised.
As requested by reviewer 2, it is good to include the latest development of membranes with ion channels. However, the authors did not really follow the suggestion. The additional sentence in Paragraph 2: To construct controllable ion transport channels, a significant amount of work has been focused on attaching ion-exchange groups to the polymer moiety14 or introducing functional fillers into polymer matrices. References 14 and 15 are referred to a really old textbook on ion-exchange membranes in 2004 and an old paper on ion-exchange membranes loaded with fillers for

desalination. These references are out of dated! The authors should cite recent work on synthetic membranes with ordered ion channels, COF membranes, or high-free-volume polymers with intrinsic micropores.
Examples: E. B. Trigg, T. W. Gaines, M. Marechal, D. E. Moed, P. Rannou, K. B. Wagener, M. J. Stevens, K. I. Winey, Nat. Mater. 2018, 17, 725.

For example, in paragraph 2: These sorts of elaborate synthetic processes increase the cost of design and manufacture, and even some common commercial ion-exchange membranes, such as perfluorinated membranes (e.g., Nafion®), are expensive16. Reference 16 is actually a development of low-cost hydrocarbon membranes with ordered subnanometer ion channels. The authors should cite appropriate references at appropriate places.
The authors performed new experiments to study the crossover of redox species through membranes. What about the crossover through Zn-PBI over long duration? More quantitative analysis of the results needs to be provided, for example, values of crossover rates.
The presentation of figures can be improved.
Figure 1 shows the design concept of metal ion coordination, it is a bit strange to include the experimental data of ion transport number in this figure (perhaps show it in Fig. 3).
Figure 2 is a mixture of experimental data and modelling. I think it is better to focus on the modelling of metal ion coordination.
Figure 4, the effect of pH on surface charge and ion transport number. More experimental data can be presented in this figure.
Figure 5: The figure contains too much information. Crossover, water migration, and dendrite growth. I suggest presenting the most key information relevant to the central idea of the work.
The paper is still very difficult to read. The English Language needs to be improved. A lot of words used in the text can be replaced with more accurate expressions. The logic flow can also be improved.
Examples: However, these practically important technologies – which technologies? Do you mean separation, purification and energy storage devices in the first sentence? There are two additional sentences after the first sentence, making it difficult to understand.
A common dilemma – perhaps change it to challenge?
The difficult working conditions – change it to harsh?
microporous structures- you need to define the structures. According to IUPAC, microporous means porosity less than 2 nm.
The "polymer chain accumulation" is still difficult to understand. I just searched it again in Google, I found very few papers (almost none) using "polymer chain accumulation". As explained by this reviewer in the first-round review, I think this expression is not accurate to explain the chain packing induced by metal ion coordination. Actually, the authors suggest that "Zn2+ coordination resulted in more uniformly distributed coordination domains, which helped reduce the local aggregation of chains", this is contradictory to the meaning of accumulation. The title can be changed to "Metal-ion coordinated polybenzimidazole membranes with preferential K+ transport (for alkaline zinc-iron flow battery, can be removed)". This will make the title easier to understand.
Comments on the modelling part will be provided by an additional reviewer.

Reviewer #4 (Remarks to the Author):

The authors have performed extensive molecular dynamics simulations of the PBI polymers to understand the different PBI-metal ion interactions. The authors have modelled many different variations of the PBI-metal ion systems. Firstly, there are 3 variations of the PBI systems solvated in water; two with varying chain lengths, and one further 'net' PBI (which I presume comes from the 'intersected' PBI model). The PBI systems were modelled again with restrained metal-ions to study the effect of metal doping on the polymer, firstly in a single layer configuration and then again in a double layer configuration. Finally, the PBI systems were also modelled solvated in DMF.

There are so many systems it is still difficult to keep track in the main text which simulations are being referred to at times. For example, in figure 3, I can only tell that 3b comes from simulations 13-15. There is no way to tell where the other results are coming from (Are they short chains or

long chains? Water or DMF? Metal-ion restrained or not? Single or double layer?).

In figure 3e, it is not immediately obvious that the authors are showing the trajectory of one single K+ ion. The K+ ions could be labelled (or better yet colour coded) by timestep.

In the computational methods, the equilibration schemes are still unclear at times. Some examples:

• "The double-layer systems were relaxed in ~100 ns MD simulation to reach equilibrated states" – NVT, NPT, NVE? What temperature / pressure?
• "After the swapping, we relaxed the double-layer system in 80-ns MD simulation" – same questions as above.

What are the authors motivations towards modelling the two 'aligned' and 'intersected' PBI structures? Supplementary Fig.16 shows two different starting configurations of the polymer chains for the simulations, but it is unclear what these two configurations are trying to represent with regards to the experimental materials? Can these two configurations be realised experimentally?

What was the motivation behind modelling the doped PBI systems in both a single layer and then a double layer configuration? Did you compare the two? Did the authors compare the transport properties in the metal-ion doped simulations with the un-doped simulations?

My overall suggestion would be that the authors look critically at the extent of the simulations included and decide which ones add best to the understanding of their system. Are 18 different simulations all necessary for the story the authors want to tell? Quality over quantity might be more useful for readers.

RESPONSE TO REVIEWERS' COMMENTS

Reviewer #1 (Remarks to the Author):

The authors have responded to all comments reasonably and revised the manuscripts by addressing all my comments. The manuscript has an improved quality, so I recommend it for publication after addressing the following minor comment.

Minor:
The difference in the surface charge calculation of PBI-D and Zn-PBI is small, which could be in the error range and could affect the derivative of the surface charge density. I recommend that the authors should include an error bar and revise the result and explanation.

Response: As suggested, the error bars of zeta potential and surface charge density were included in Supplementary Fig. 25 in the revised version (each data point has been repeated three times).

The revised version in Supplementary Fig. 25 is shown below:

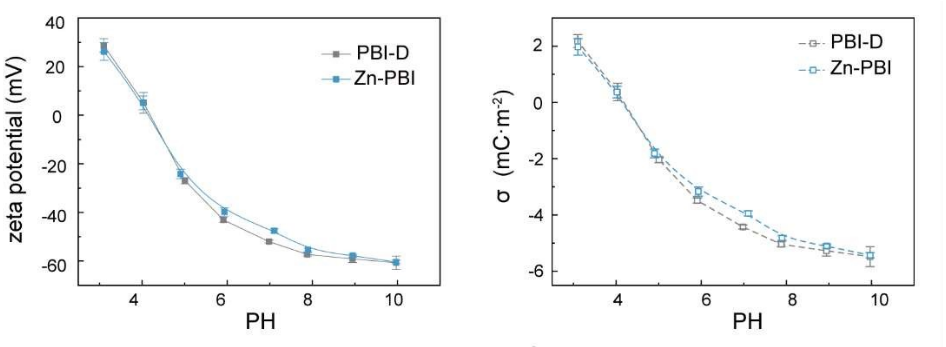

As the reviewer mentioned, the difference in the surface charge calculation of PBI-D and Zn-PBI is small. To avoid misunderstanding, we revised the explanation about the surface charge density instead of taking the derivative of the surface charge density. It was revised as "When the pH increased from 3 to 10, Zn-PBI became more negatively charged due to deprotonation of the imidazole groups at high pH." (Line 8 to 9 on page 15).

Reviewer #2 (Remarks to the Author):
The authors have properly taken into consideration all comments from the referees and have addressed all relevant issues.

This referee supports publication of the work in the present form.

Response: We appreciate the reviewer's suggestions and it helps us to improve our paper.

Reviewer #3 (Remarks to the Author):

I have reviewed this revised manuscript and their responses to comments given by all reviewers. The authors certainly have made efforts to address some of the concerns raised by all reviewers. The quality of the manuscript is improved, however, there are still many problems.

It is quite important to provide more experimental data to study the ion channel structures. As suggested by Reviewer 1, SAXS/WAXS, HRTEM, and gas sorption might be useful. The authors performed additional experiments including PALS to study the pore size or free volume elements. However, the PALS results are insufficient. WAXS might be useful to study the variation of inter/intrachain spacings.

Response: As suggested, Wide Angle X-ray scattering (WAXS) and Small Angle X-ray scattering (SAXS) were carried out to study the variation of inter/intrachain spacings, which was attached below:

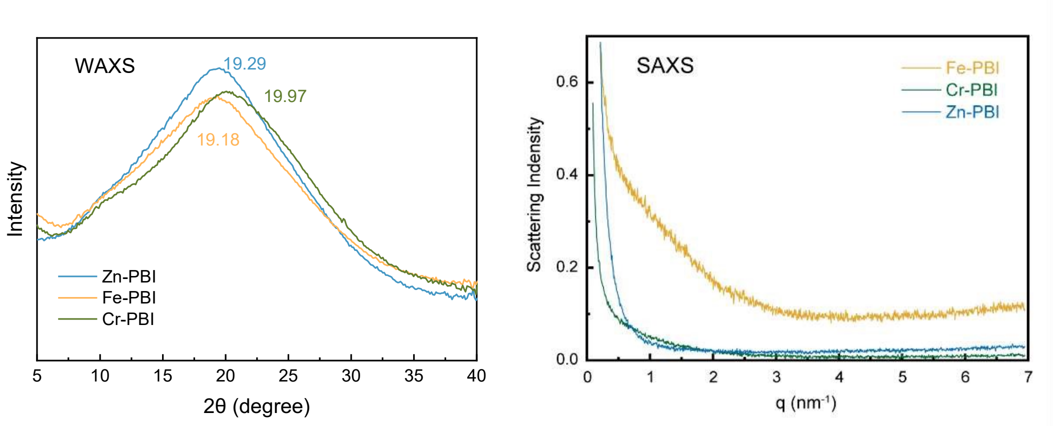

However, it seems that the WAXS/SAXS can not well reflect the difference of these three membranes. The broad peaks in WAXS are at 2θ ≈ 19° and specifically, Zn-PBI (2θ=19.29°), Fe-PBI (2θ=19.18°), and Cr-PBI (2θ=19.97°).
Actually, in our opinion, the transmembrane H_2 flux and positron annihilation lifetime (PAL) spectroscopy can show the different pore structures and give the quantitative pore size.

As requested by reviewer 1, the authors updated typical charge/discharge curves as insets in the cycling data. But this is very difficult to read. It would be better to plot the charge/discharge data as voltage versus capacity and compare them in one plot.

Response: We appreciate the reviewer's suggestion. As suggested, the charge/discharge data has been changed to voltage versus capacity in one plot as Fig. 5f in the revised version (on page 13).

Reviewer 2 raised one important question on water migration. The authors' response and discussion on ion transport and water migration seem to be reasonable, however, is it possible to provide any experimental evidence to support the claims? More quantitative analysis of the water diffusion coefficient through the membranes would also be valuable. Similarly in the discussion on Page 11, more quantitative data should be provided, for example, what were the crossover rates of Zn and iron species? Note that there are mistakes in the diagram, Fe(CN)6 instead of Fe(OH)6.

Response: We appreciate the reviewer's comments. To support our conclusion, more quantitative analysis of crossover experiments has been carried out, demonstrating that Zn-PBI can effectively block active species to mitigate the water carried by active species. Details are as follows:

The permeation coefficient (P) of zinc species $[Zn(OH)_4]^{2-}$ was calculated by measuring the concentration of zinc species through the membrane with inductively coupled plasma mass spectrometry (ICP-MS). The P of PBI-D and Zn-PBI to zinc species is 1.98×10^{-3} cm^2/h, and 4.27×10^{-7} cm^2/h respectively, confirming the above results.

We further tested the permeability of iron species $[Fe(CN)_6]^{4-}$ by UV-vis spectrometer. The P of Zn-PBI is 6.45×10^{-8} cm^2/h, which is much lower than that of PBI-D (1.44×10^{-3} cm^2/h). Note that permeability was obtained by a 120 hours test). According to the experimental results, compared with PBI-D, Zn-PBI can effectively inhibit active species impelled by the concentration gradient, thus Zn-PBI can suppress the water migration carried by the ionic hydration layer of active species.

Unfortunately, when measuring the diffusion coefficient of water through the membrane, unilateral pressure was required. There is no such high pressure on one side during the operation of the battery. The diffusion coefficient thus measured is not suitable for explaining the performance of the membrane in the battery.

In our opinion, it is difficult to precisely separate the contribution of water migration. Because when a battery works, the actual environment of the ion migration is more complex.

Overall, when we discussed water migration, we discussed the effects of osmotic pressure, electric field, and concentration gradient on water migration as much as possible to logically explain why Zn-PBI can alleviate water migration.

To clarify this, all the quantitative analysis data was added in the revised version. (Line 12 to 17 on page 14). The correct $[Fe(CN)_6]^{4-}$ and $[Fe(CN)_6]^{3-}$ has been provided in the revised version. The permeability of $[Fe(CN)_6]^{4-}$ were given in Supplementary Fig. 23c, which was attached below:

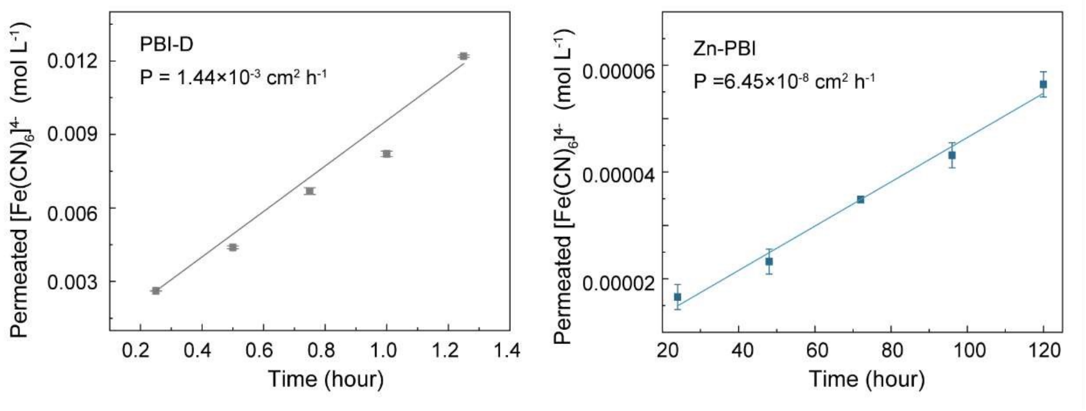

The permeability of $[Zn(OH)_4]^{2-}$ were given in Supplementary Fig. 23d, which was attached below:

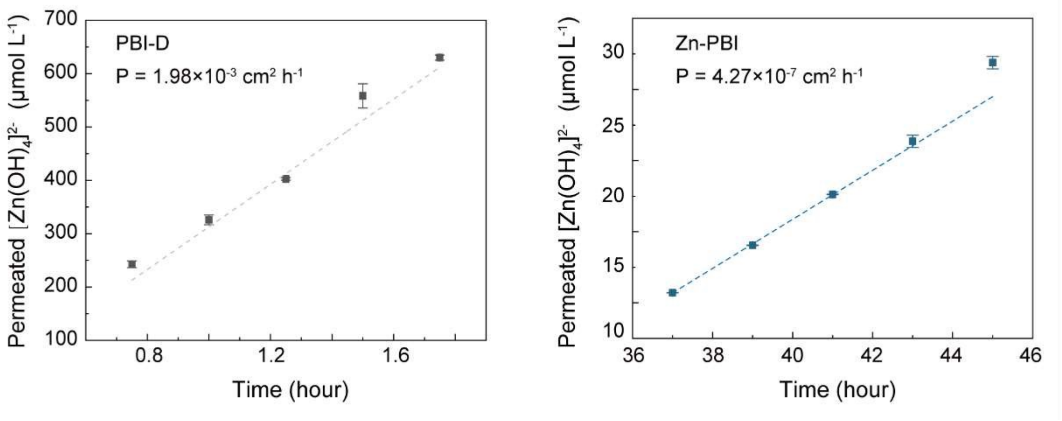

Reviewer 2 also made a good comment on the detailed parameters that influence the structural transformation. The authors did provide some great preliminary results to show the effect of metal ion concentration, reaction time, and polymer concentration. It is good to see that the authors included some of these results in the supporting information.

The authors updated the introduction to recent work on metal ion coordinated polymers including the Cu-Cellulose and Cu-PBI polymers (JACS). However, the novelty of this work over the previous work still needs to be emphasized. In my opinion, the novelty of this work lies in more in-depth understanding of the polymer chain packing by molecular modelling and characterization of the membranes coordinated by different metal ions as well as ion transport, which was not carefully studied in their previous JACs work.

Response: We appreciate the reviewer's comments. We carefully reorganized the Introduction. As suggested, the novelty of this work has been emphasized further in the revised version. (Line 6 to 27 from on page 2)

The references cited by the authors are still poorly organised.
As requested by reviewer 2, it is good to include the latest development of membranes with ion channels. However, the authors did not really follow the suggestion. The additional sentence in Paragraph 2: To construct controllable ion transport channels, a significant amount of work has been focused on attaching ion-exchange groups to the polymer moiety14 or introducing functional fillers into polymer matrices. References 14 and 15 are referred to a really old textbook on ion-exchange membranes in 2004 and an old paper on ion-exchange membranes loaded with fillers for desalination. These references are out of dated! The authors should cite recent work on synthetic membranes with ordered ion channels, COF membranes, or high-free-volume polymers with intrinsic micropores.
Examples: E. B. Trigg, T. W. Gaines, M. Marechal, D. E. Moed, P. Rannou, K. B. Wagener, M. J. Stevens, K. I. Winey, Nat. Mater. 2018, 17, 725.
For example, in paragraph 2: These sorts of elaborate synthetic processes increase the cost of design and manufacture, and even some common commercial ion-exchange membranes, such as perfluorinated membranes (e.g., Nafion®), are expensive16. Reference 16 is actually a development of low-cost hydrocarbon membranes with ordered subnanometer ion channels. The authors should cite appropriate references at appropriate places.

Response: Sorry for the confusion. As suggested, the references were updated in the reorganized Introduction of the revised version.

The authors performed new experiments to study the crossover of redox species through membranes. What about the crossover through Zn-PBI over long duration? More quantitative analysis of the results needs to be provided, for example, values of crossover rates.

Response: We appreciate the reviewer's advice. The permeability of $Fe(CN)_6^{4-}$ over a longer duration has been studied, and the permeation coefficient of $Fe(CN)_6^{4-}$ for Zn-PBI was thus calculated.

The permeability of $[Fe(CN)_6]^{4-}$ were given in Supplementary Fig. 23c, which was attached below:

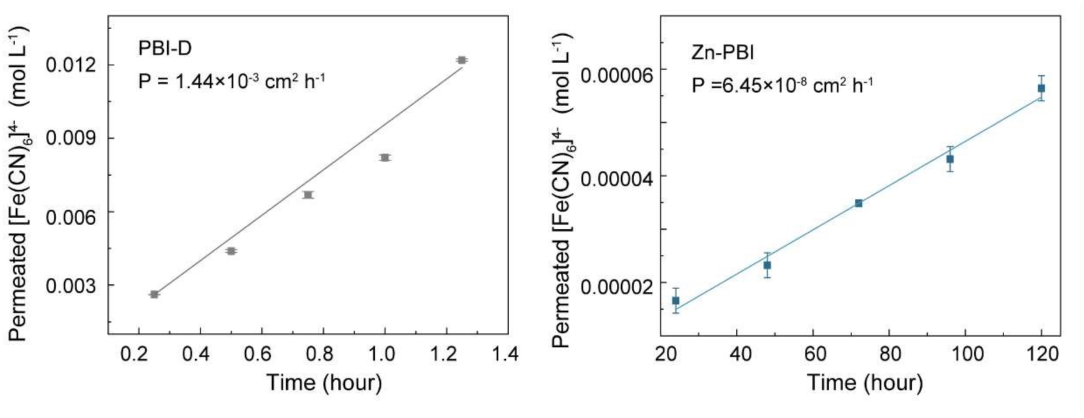

The presentation of figures can be improved.
Figure 1 shows the design concept of metal ion coordination, it is a bit strange to include the experimental data of ion transport number in this figure (perhaps show it in Fig. 3).
Figure 2 is a mixture of experimental data and modelling. I think it is better to focus on the modelling of metal ion coordination.
Figure 4, the effect of pH on surface charge and ion transport number. More experimental data can be presented in this figure.
Figure 5: The figure contains too much information. Crossover, water migration, and dendrite growth. I suggest presenting the most key information relevant to the central idea of the work.

Response: We appreciate the reviewer's advice, we follow the suggestion to reorganized the all the figures.

Fig. 1 showed the concenption, and Fig. 2 showed the modelling data.

The the results of Molecular dynamics (MD) simulations have been presented in Fig. 3 and Fig. 4.

Fig.5 showed the appplication data.

The paper is still very difficult to read. The English Language needs to be improved. A lot of words used in the text can be replaced with more accurate expressions. The logic flow can also be improved.
Examples: However, these practically important technologies – which technologies? Do you mean separation, purification and energy storage devices in the first sentence? There are two additional sentences after the first sentence, making it difficult to understand.
A common dilemma – perhaps change it to challenge?
The difficult working conditions – change it to harsh?
microporous structures- you need to define the structures. According to IUPAC, microporous means porosity less than 2 nm.
The "polymer chain accumulation" is still difficult to understand. I just searched it again in Google, I found very few papers (almost none) using "polymer chain accumulation". As explained by this reviewer in the first-round review, I think this expression is not accurate to explain the chain packing induced by metal ion coordination. Actually, the authors suggest that "Zn2+ coordination resulted in more uniformly distributed coordination domains, which helped reduce the local aggregation of chains", this is contradictory to the meaning of accumulation.
The title can be changed to "Metal-ion coordinated polybenzimidazole membranes with preferential K+ transport (for alkaline zinc-iron flow battery, can be removed)". This will make the title easier to understand.
Comments on the modelling part will be provided by an additional reviewer.
Response: We try to polish our English further. Our paper has been edited by Language Editing from Springer Nature Author Services. Additionally, with remaining the content and science of the article unchanged, we carefully reorganized the language and figures of the revised version to improve the quality of the article. The language problems raised

by reviewers have been modified in the revised version.

For example:

microporous structures → macroporous structures (~0.8 μm) (Line 2 on page 6)

To avoid misunderstanding, the title has been revised to "Metal-coordinated polybenzimidazole membranes with preferential K^+ transport".

Reviewer #4 (Remarks to the Author):

The authors have performed extensive molecular dynamics simulations of the PBI polymers to understand the different PBI-metal ion interactions. The authors have modelled many different variations of the PBI-metal ion systems. Firstly, there are 3 variations of the PBI systems solvated in water; two with varying chain lengths, and one further 'net' PBI (which I presume comes from the 'intersected' PBI model). The PBI systems were modelled again with restrained metal-ions to study the effect of metal doping on the polymer, firstly in a single layer configuration and then again in a double layer configuration. Finally, the PBI systems were also modelled solvated in DMF.

There are so many systems it is still difficult to keep track in the main text which simulations are being referred to at times. For example, in figure 3, I can only tell that 3b comes from simulations 13-15. There is no way to tell where the other results are coming from (Are they short chains or long chains? Water or DMF? Metal-ion restrained or not? Single or double layer?).

Response: Sorry for the confusion. To clarify this, we have made a few modifications to better understand the simulation. In general, we have removed the 'net' PBI (also as 'intersected' PBI), long chains systems, and DMF systems in the original #2, #6-9, #16-18 in Supplementary Table 5 and Supplementary Figs.12b-c, 14, and 15 since they do not affect the primary results and conclusions. All simulations in the revised manuscript then would focus on PBI membranes of the same chain length (short chains) in water. Metal ions without restraint and with restraint are both investigated in a single-layer model. All double-layer systems were made up of two replicas of single-layer systems (with metal-restraint) to study the K+ transport in different metal-doped PBI membranes.
We have made a few modifications to improve the clarity of simulations in the simulation section of Supporting Information as well as Fig. 3b-f, Fig. 4 and Supplementary Fig. 17-20.

In figure 3e, it is not immediately obvious that the authors are showing the trajectory of one single K+ ion. The K+ ions could be labelled (or better yet colour coded) by timestep.

Response: We thank the reviewer for the helpful suggestion. As suggested, we have labeled the K^+ ions by timestep as displayed in Fig. 4e of the revised manuscript.

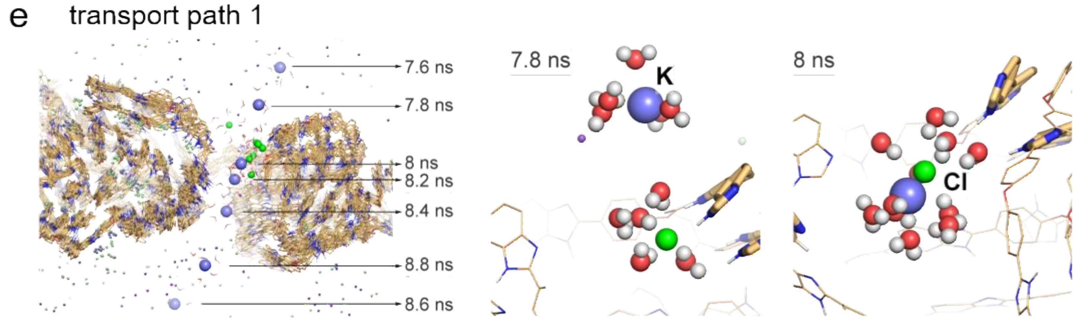

Figure 4e Snapshots of one K^+ (in blue) passing through the Zn-PBI membrane under the

regulation of Cl^- (in green). Magnified sequential snapshots at 7.8 ns and 8 ns are displayed for K^+ with water in the first solvation shell.

In the computational methods, the equilibration schemes are still unclear at times. Some examples:

• "The double-layer systems were relaxed in ~100 ns MD simulation to reach equilibrated states" – NVT, NPT, NVE? What temperature / pressure?

Response: We thank the reviewer for the valuable suggestion.

Following the suggestion, we have added the details for the double-layer systems in the simulation section of Supporting Information as follows:

The double-layer systems were relaxed in ~100 ns MD simulation under NVT ensemble (298 Kelvin, 1 bar, Langevin dynamics thermostat) to reach equilibrated states.

• "After the swapping, we relaxed the double-layer system in 80-ns MD simulation" – same questions as above.

Response:

Following the suggestion, we have added the details for the double-layer systems in the simulation section of Support Information as follows:

After the swapping, we relaxed the double-layer system in an 80-ns MD simulation with the same NVT ensemble.

What are the authors motivations towards modelling the two 'aligned' and 'intersected' PBI structures? Supplementary Fig.16 shows two different starting configurations of the polymer chains for the simulations, but it is unclear what these two configurations are trying to represent with regards to the experimental materials? Can these two configurations be realised experimentally?

Response: We appreciate the reviewer's helpful suggestions.

In the revised simulation section of Supporting Information, we have removed the 'net' PBI (also as 'intersected' PBI) (the original #2 in Supplementary Table 5 and Supplementary Fig. 12c) since they do not affect the primary results and conclusions. We focus on the effect of metal ions on the aligned PBI structure to better understand the present study.

What was the motivation behind modelling the doped PBI systems in both a single layer and then a double layer configuration? Did you compare the two? Did the authors compare the transport properties in the metal-ion doped simulations with the un-doped simulations?

Response: We thank the reviewer for the helpful suggestions.

Single-layer systems are simulated to study the structures of the PBI membranes and PBI-metal ion interactions. Because in a periodic box, where the solvent above the membrane is together with the solvent below the membrane, the regular single-layer systems do not provide the condition to generate ion concentration differences across a membrane. Thus, double-layer systems were constructed to study the K^+ transport, where two aqueous compartments are used to generate ion concentration differences across a membrane.

The double-layer system was made up of two single-layer systems of the same. Structurally, it would be equivalent to two replicas of one single-layer system. Thus, the membranes are the same in single-layer and double-layer systems. Since we did not observe continuous water channel in un-doped simulations, the un-doped PBI was not further used to study K^+ transport.

My overall suggestion would be that the authors look critically at the extent of the simulations included and decide which ones add best to the understanding of their system. Are 18 different simulations all necessary for the story the authors want to tell? Quality over quantity might be more useful for readers.

Response: We appreciate the reviewer's helpful suggestions.

As mentioned above, to improve the clarity and readability of simulations, we have removed the 'net' PBI (also as 'intersected' PBI), long chains systems, and DMF systems since they do not affect the primary results and conclusions. Simulations in the revised manuscript would focus on structures, interactions, and K^+ transport in metal-doped PBI membranes in water. More details are also added regarding the double-layer systems.

REVIEWERS' COMMENTS

Reviewer #3 (Remarks to the Author):

The authors have addressed most of my concerns and significantly improved the manuscript. I am pleased to see that the authors followed my suggestions and rearranged the main figures, which are much better to present the concept and the findings. The logical flow of the introduction is much easier to follow and the English language has also been improved. The revised manuscript is now suitable for publication.

Minor changes:
The authors have also updated references with more key relevant work cited. However, there are some minor mistakes with the format of references.

References: high-free-volume polymers with intrinsic micropores13,14,15
The authors should also cite this work on ion-selective PIM membranes:
Tan, R., Wang, A., Malpass-Evans, R. et al. Hydrophilic microporous membranes for selective ion separation and flow-battery energy storage. Nat. Mater. 19, 195–202 (2020). https://doi.org/10.1038/s41563-019-0536-8

Reference:15: Zuo P, et al. Sulfonated microporous polymer membranes with fast and selective ion transport for
electrochemical energy conversion and storage. Angew. Chem. Int. Ed. n/a, (2020). The page number is missing.

Reference Case D, et al. Amber 2018 (2018). This may be incomplete?

Reviewer #4 (Remarks to the Author):

The authors have addressed all of my concerns with regards to the simulation methods and results. Everything looks good, thank you.

REVIEWER COMMENTS

Reviewer #3 (Remarks to the Author):

The authors have addressed most of my concerns and significantly improved the manuscript. I am pleased to see that the authors followed my suggestions and rearranged the main figures, which are much better to present the concept and the findings. The logical flow of the introduction is much easier to follow and the English language has also been improved. The revised manuscript is now suitable for publication.

Minor changes:

The authors have also updated references with more key relevant work cited. However, there are some minor mistakes with the format of references.

References: high-free-volume polymers with intrinsic micropores13,14,15

The authors should also cite this work on ion-selective PIM membranes:

Tan, R., Wang, A., Malpass-Evans, R. et al. Hydrophilic microporous membranes for selective ion separation and flow-battery energy storage. Nat. Mater. 19, 195–202 (2020). https://doi.org/10.1038/s41563-019-0536-8

Response: As suggested, the reference has been cited as reference 16 in the revised manuscript.

Reference:15: Zuo P, et al. Sulfonated microporous polymer membranes with fast and selective ion transport for electrochemical energy conversion and storage. Angew. Chem. Int. Ed. n/a, (2020). The page number is missing.

Response: As suggested, the reference has been updated as reference 15 in the revised manuscript.

Reference Case D, et al. Amber 2018 (2018). This may be incomplete?

Response: The cited book has been revised in the revised manuscript: Case D, et al. Amber 2018. (University of California, San Francisco, 2018).

Reviewer #4 (Remarks to the Author):

The authors have addressed all of my concerns with regards to the simulation methods and results. Everything looks good, thank you.

Response: We appreciate the reviewer's suggestions and it helps us to improve our paper.
